# Supplementary material for: Human-iPSC-Derived Cardiac Stromal Cells Enhance Maturation in 3D Cardiac Microtissues and Reveal Non-cardiomyocyte Contributions to Heart Disease
Source: Cell Stem Cell. 2020 Jun 4;26(6):862–879.e11. doi: 10.1016/j.stem.2020.05.004 (PMC7284308; doi:10.1016/j.stem.2020.05.004)
Supplement: Document S1. Figures S1–S7 and Table S6 [file mmc1.pdf]

Cell Stem Cell, Volume *26*

Supplemental Information

Human-iPSC-Derived Cardiac Stromal Cells Enhance Maturation in 3D Cardiac Microtissues and Reveal Non-cardiomyocyte Contributions to Heart Disease

Elisa Giacomelli, Viviana Meraviglia, Giulia Campostrini, Amy Cochrane, Xu Cao, Ruben W.J. van Helden, Ana Krotenberg Garcia, Maria Mircea, Sarantos Kostidis, Richard P. Davis, Berend J. van Meer, Carolina R. Jost, Abraham J. Koster, Hailiang Mei, David G. Míguez, Aat A. Mulder, Mario Ledesma-Terrón, Giulio Pompilio, Luca Sala, Daniela C.F. Salvatori, Roderick C. Slieker, Elena Sommariva, Antoine A.F. de Vries, Martin Giera, Stefan Semrau, Leon G.J. Tertoolen, Valeria V. Orlova, Milena Bellin, and Christine L. Mummery

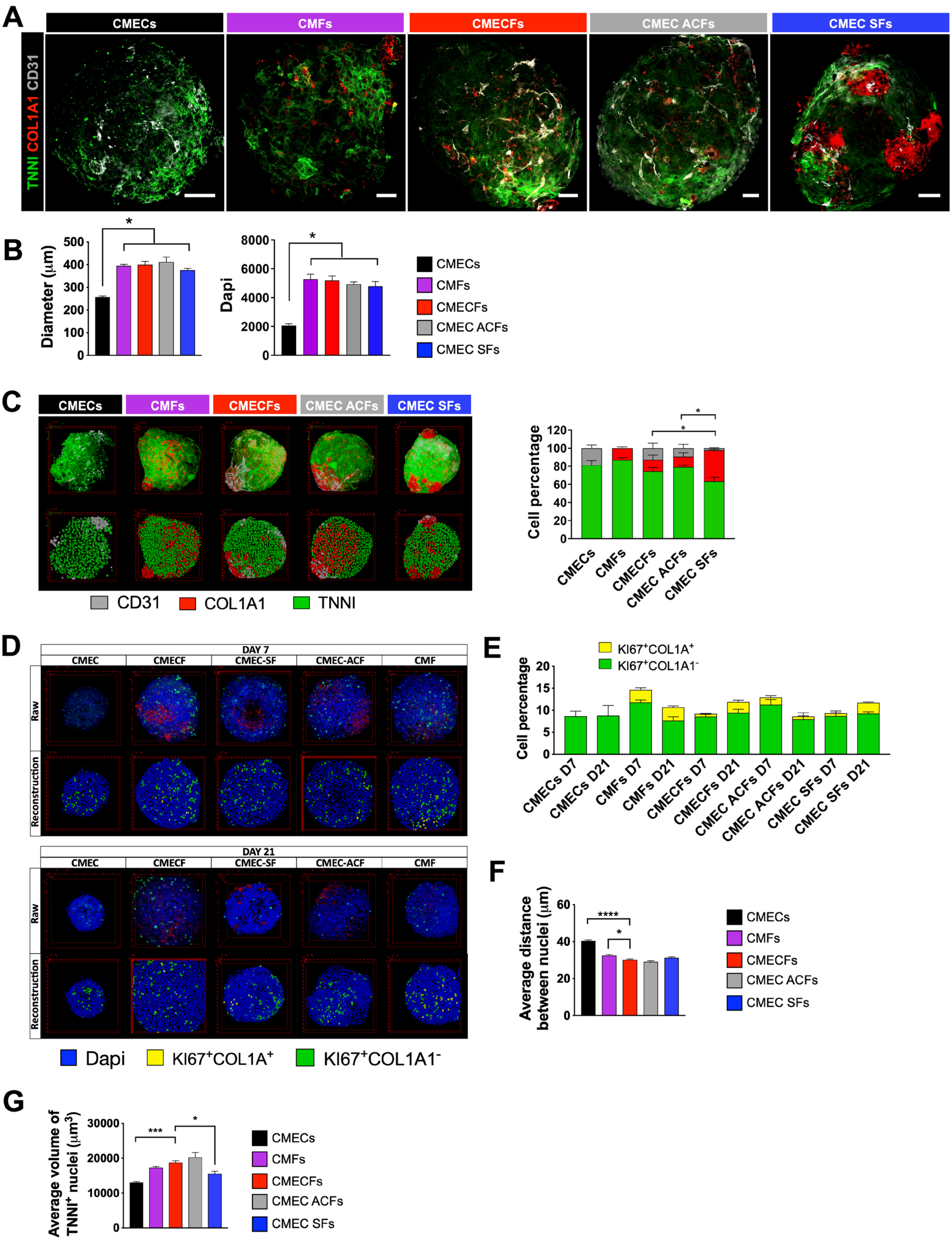
A
CMECs
CMFs
CMECFs
CMEC ACFs
CMEC SFs
TNNI COL1A1 CD31
B
Diameter (µm)
Dapi
CMECs
CMFs
CMECFs
CMEC ACFs
CMEC SFs
C
CD31
COL1A1
TNNI
Cell percentage
D
DAY 7
DAY 21
CMEC
CMECF
CMEC-SF
CMEC-ACF
CMF
Raw
Reconstruction
Dapi
KI67+COL1A+
KI67+COL1A1-
E
Cell percentage
CMECs D7
CMECs D21
CMFs D7
CMFs D21
CMECFs D7
CMECFs D21
CMEC ACFs D7
CMEC ACFs D21
CMEC SFs D7
CMEC SFs D21
F
Average distance between nuclei (µm)
G
Average volume of TNNI+ nuclei (µm3)

Figure S1 (related to Figure 2). Characterization of 3D microtissues. (A) Representative immunofluorescence images of CM- (TNNI, green), EC- (CD31, grey) and fibroblast- (COL1A1, red) markers in MTs, as indicated. Scale bar: 50 µm. (B) Bar graphs showing MT diameter measured in MTs mounted on glass coverslips (left, N>9) and total number of $DAPI^{+}$ cells measured by 3D semi-automated image processing (right, N=4). *P<0.0001. One-way ANOVA with Dunnett's multiple comparisons test. (C) Representative immunofluorescence images (top) and digital images (bottom) showing $TNN1^{+}$ (green), $COL1A1^{+}$ (red), and $CD31^{+}$ (grey) cells in MTs (left). Bar graph quantification of the same cell populations (right). N=4, *P<0.001 for COL1A1+ cells. Two-way ANOVA with Tukey's multiple comparisons test. (D) Representative immunofluorescence (top, raw) and digital (bottom, reconstruction) images for $KI67{+}COL1A1^{+}$ (yellow) and $KI67^{+}COL1A1^{-}$ (green) cells. $DAPI^{+}$ cells in blue. MTs at day 7 (upper panel) and day 21 (lower panel) were analysed. (E) Bar graph showing the percentage of $KI67^{+}COL1A1^{-}$ and (green) and $KI67^{+}COL1A^{+}$ (yellow) cells in MTs at the indicated time points (D=day). N>3. (F, G) Bar graph showing average distance between nuclei (F) and average volume of $TNNI^{+}$ nuclei (G) in MTs. N=4 MTs, ****P<0.0001, ***P<0.0005, *P<0.05. One-way ANOVA with Dunnett's multiple comparisons test. All data are shown as mean ± SEM.

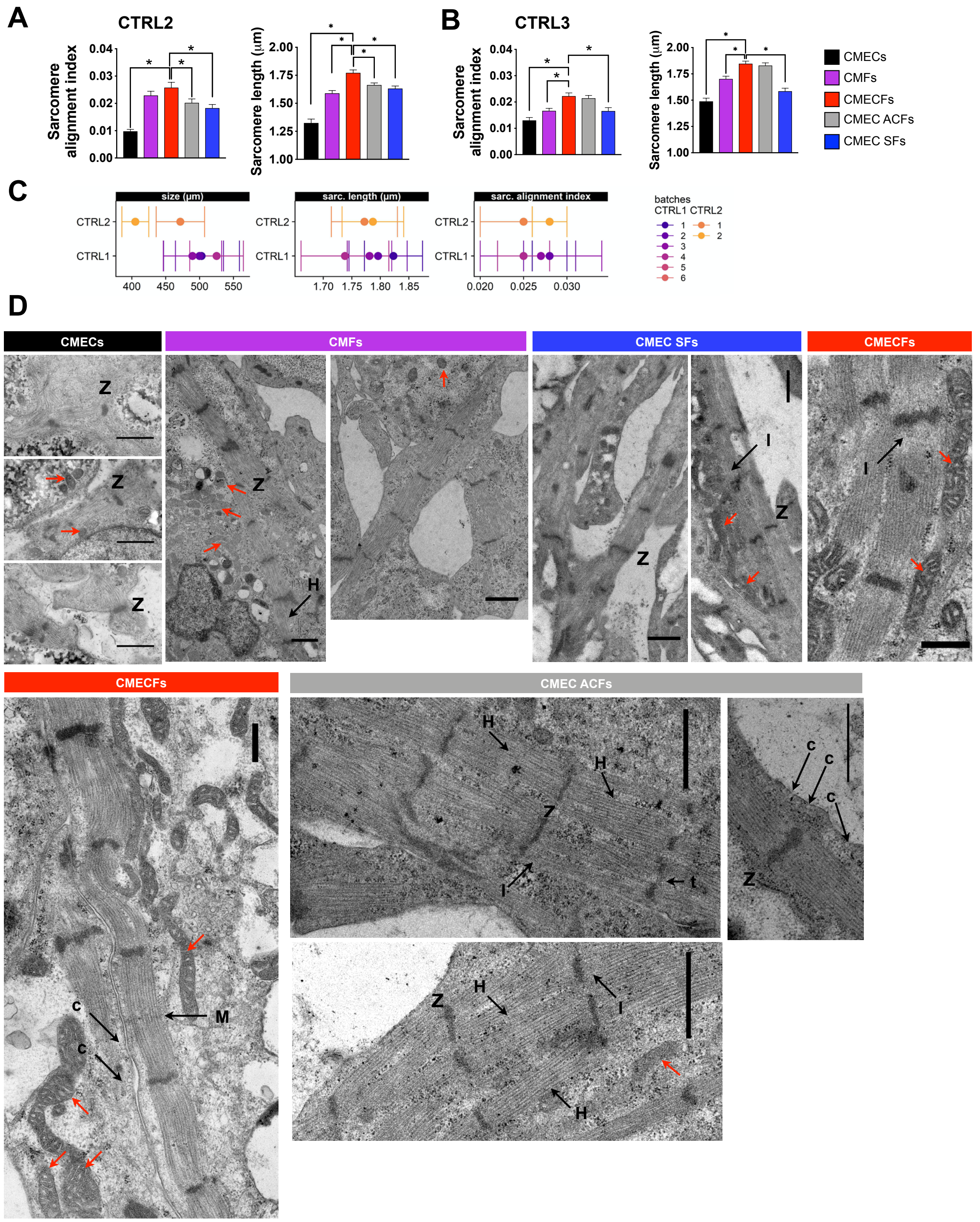

Figure S2 (related to Figure 2). Structural maturation and ultrastructure of 3D microtissues. (A, B) Quantification of sarcomere organization (sarcomere alignment index, left) and sarcomere length (right) in CTRL2 hiPSCs (N>30, *P<0.05) (A) and CTRL3 hiPSCs (N=30, *P<0.005) (B) MTs from immunofluorescence analysis. Data are shown as mean ± SEM. N indicates different areas from 3 independent MTs per group. One-way ANOVA with Dunnett's multiple comparisons test. (C) Graphs showing variability of morphological parameters, i.e. size, sarcomere length, and sarcomere alignment index. Mean values (dots) and SD (error bars) for different MT batches from CTRL1 and CTRL2 hiPSCs are shown. (D) Representative TEM images showing presence or absence of ultrastructural features typical of mature cardiomyocytes in CMECs, CMFs, CMEC SFs, CMECFs and CMEC ACFs: caveolae (c), Z-lines (Z), M-lines (M), I-bands (I), H-zones (H) and mitochondria (red arrows). CMECs, CMFs, CMEC SFs and CMEC ACFs, scale bar: 1 µm. CMECFs, scale bar: 0.5 µm.

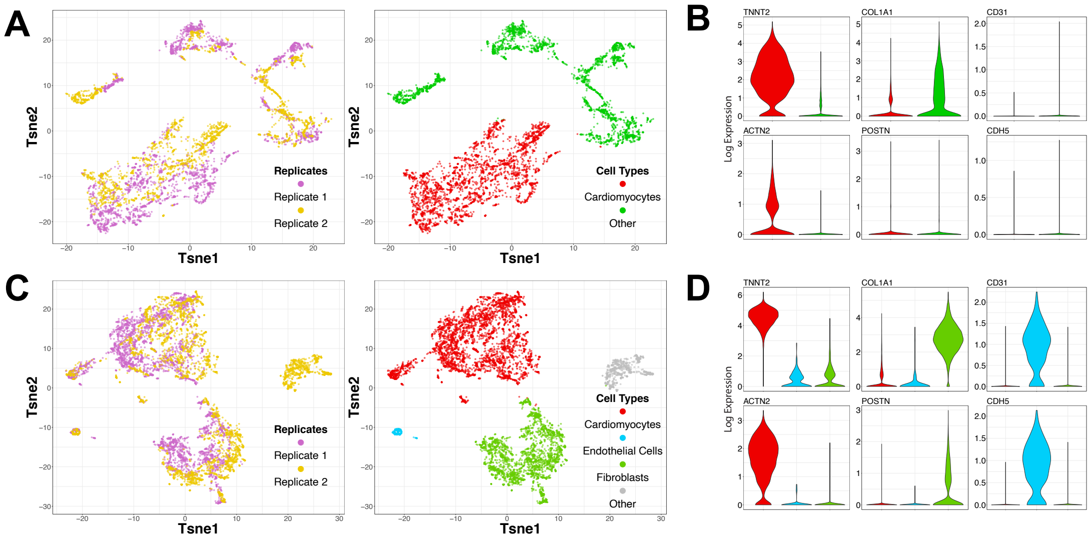

E

Comparison model		Number of upregulated DEGs	
Group1	Group2	Group 1	Group 2
hiPSC-CMs	CMECs	3891	6038
hiPSC-CMs	CMECFs	4488	4600
CMECs	CMECFs	4192	2537
CMECFs	CMEC-SFs	2349	1313
CMECFs	CMEC-ACFs	92	116

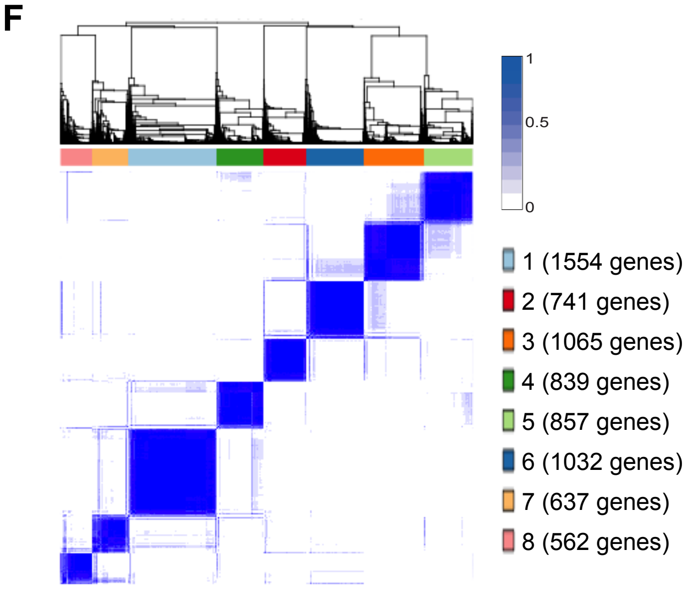

Figure S3 (related to Figure 3). Single-cell and bulk transcriptome profiling of microtissues. (A) Tsne plots of hiPSC-CMs based on scRNA-seq profiles. In the left panel, colors represent different replicate samples (*Replicate 1; Replicate 2*); in the right panel, colors represent different cell type clusters (*Cardiomyocytes; Other*). (B) Violin plots showing expression (log transformed) of selected CM- (*TNNT2*, *ACTN2*), fibroblast- (*COL1A1, POSTN*), and EC- (*CD31, CDH5)* markers in hiPSC-CMs based on their scRNA-seq profiles. (C) Tsne plots of CMECFs based on their scRNA-seq profiles. In the left panel, colors represent different replicate samples (*Replicate 1; Replicate 2*); in the right panel, colors represent different cell type clusters (*Cardiomyocytes; Endothelial cells; Fibroblasts; Other*). (D) Violin plots showing expression (log transformed) of selected CM- (*TNNT2*, *ACTN2*), fibroblast- (*COL1A1, POSTN*), and EC- (*CD31, CDH5)* markers in CMECFs based on their single-cell RNA-seq profile. (E) Total number of DEGs identified across hiPSC-CMs, CMECs, CMECFs, CMEC SFs and CMEC ACFs in bulk RNA-seq analysis. (F) Consensus matrix: DEGs from (E) grouped into eight gene clusters with ConsensusCluster, each row and column represent a unique gene and the color scale represents consensus values, which indicate how often two items occupied the same cluster out of 500 iterations. The color key indicates cluster definitions and the number of genes in each cluster.

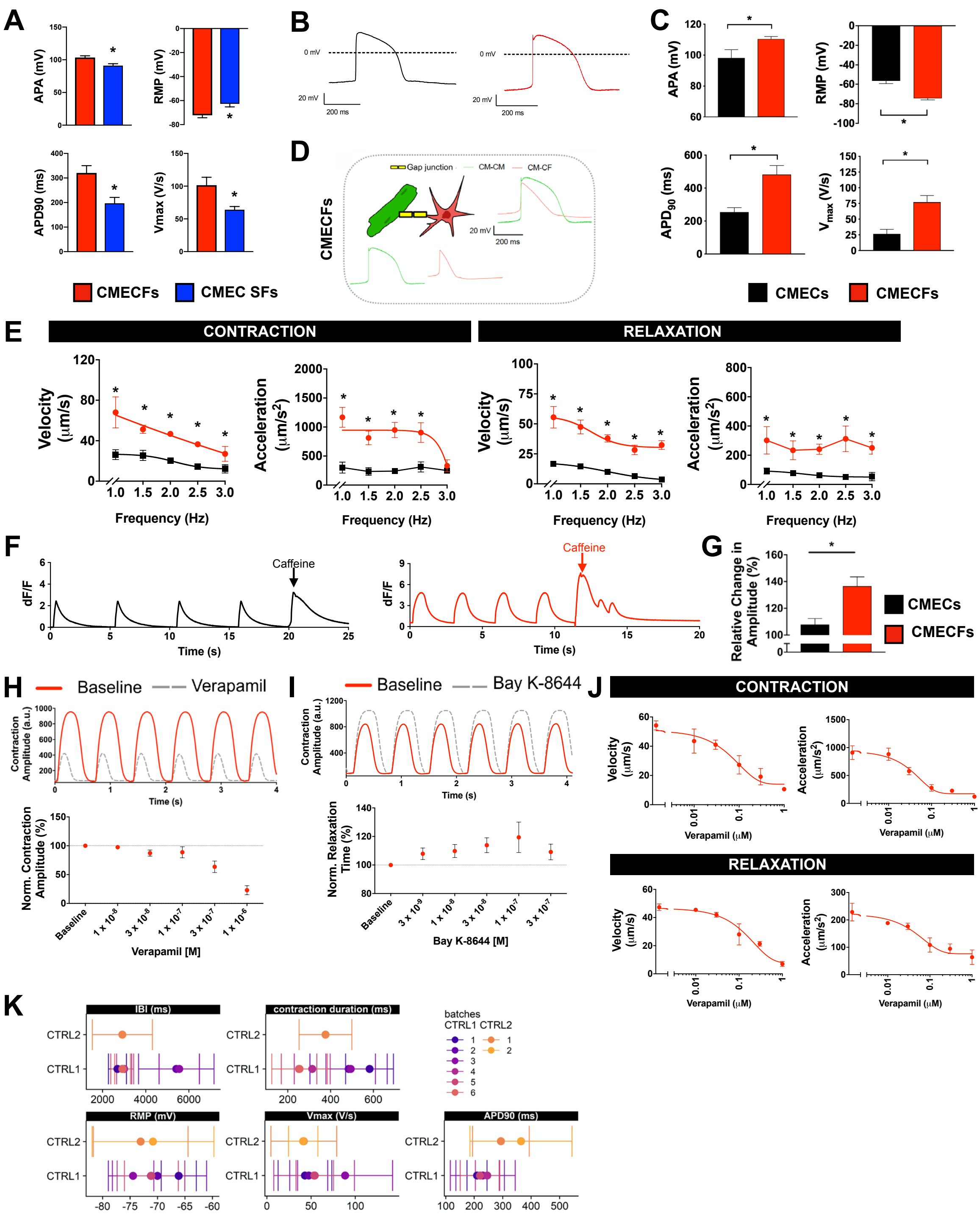
A
APA (mV)
RMP (mV)
APD90 (ms)
Vmax (V/s)
CMECFs
CMEC SFs
B
0 mV
20 mV
200 ms
C
APA (mV)
RMP (mV)
APD90 (ms)
Vmax (V/s)
CMECs
CMECFs
D
CMECFs
Gap junction
CM-CM
CM-CF
E
CONTRACTION
RELAXATION
Velocity (µm/s)
Acceleration (µm/s2)
Frequency (Hz)
F
Caffeine
dF/F
Time (s)
G
Relative Change in Amplitude (%)
CMECs
CMECFs
H
Baseline
Verapamil
Contraction Amplitude (a.u.)
Time (s)
Norm. Contraction Amplitude (%)
Verapamil [M]
I
Baseline
Bay K-8644
Contraction Amplitude (a.u.)
Time (s)
Norm. Relaxation Time (%)
Bay K-8644 [M]
J
CONTRACTION
RELAXATION
Velocity (µm/s)
Acceleration (µm/s2)
Verapamil (µM)
K
IBI (ms)
contraction duration (ms)
RMP (mV)
Vmax (V/s)
APD90 (ms)
CTRL1
CTRL2
batches
CTRL1 CTRL2

Figure S4 (related to Figure 4). Cardiac fibroblasts promote electrical maturation and enhance contractility of hiPSC-CMs in microtissues. (A) Bar graph showing parameters of APs recorded from single hiPSC-CMs (CTRL2) dissociated from CMECFs and CMEC SFs: APA, RMP, APD_{90}; V_{max} (in APs measured with dynamic clamp). N>18, single CMs dissociated from 2 independent MT batches per group, *P>0.05. Student's t-test. Data are shown as mean ± SEM. (B, C) Representative AP traces measured by sharp electrode electrophysiology in CMECs (black) and in CMECFs (red) generated from CTRL1 (B) and AP parameters (C). N>11, MTs per group, *P<0.05. Student's t-test. Data are shown as mean ± SEM. (D) Diagram and representative examples of APs measured in CMECFs by sharp electrode electrophysiology documenting direct coupling between CMs (CM-CM) or CM and CF (CM-CF), together with APs overlay. (E) Rate-dependence analysis for velocity and acceleration of contraction (left panel) together with velocity and acceleration of relaxation (right panel) in CMECs (black) and CMECFs (red). N>22, MTs per group, *P<0.0001. Two-way ANOVA with Sidak's multiple comparison test. Data are shown as mean ± SD. (F) Representative Ca^{2+} transients before and after addition of 50 mM Caffeine in CMECs (black) and CMECFs (red). (G) Change in Ca^{2+} amplitude (percentage, relative to amplitude at baseline condition) measured in CMECs (black) and CMECFs (red). N=8, MTs per group. *P<0.005. Student's t-test. Data are shown as mean ± SEM. (H, I) Representative contraction traces from CMECFs stimulated at 1.5 Hz under baseline condition (red lines) and upon addition of 1 x 10^{-6} M verapamil (H, top) or 1 x 10^{-7} M Bay K-8644 (I, top) (dashed grey lines). Concentration-response curves (contraction amplitude was normalized to baseline) in CMECFs under baseline condition and upon increasing concentrations of Verapamil (H, bottom, N=10) or Bay K-8644 (I, bottom, N=6). Data are shown as mean ± SEM. (J) Concentration-response curves for velocity and acceleration of contraction (top) and relaxation (bottom) of CMECFs under baseline condition and upon increasing concentrations of Verapamil. MTs were stimulated at 1.5 Hz. N>14. Data are shown as mean ± SEM. (K) Graphs showing variability of contraction parameters, i.e. IBI and contraction duration (top panels) and single-cell electrophysiology parameters, i.e. RMP, Vmax, and APD_{90} in CMECFs. Mean values (dots) and SD (error bars) for different MT batches from CTRL1 and CTRL2 hiPSCs are shown.

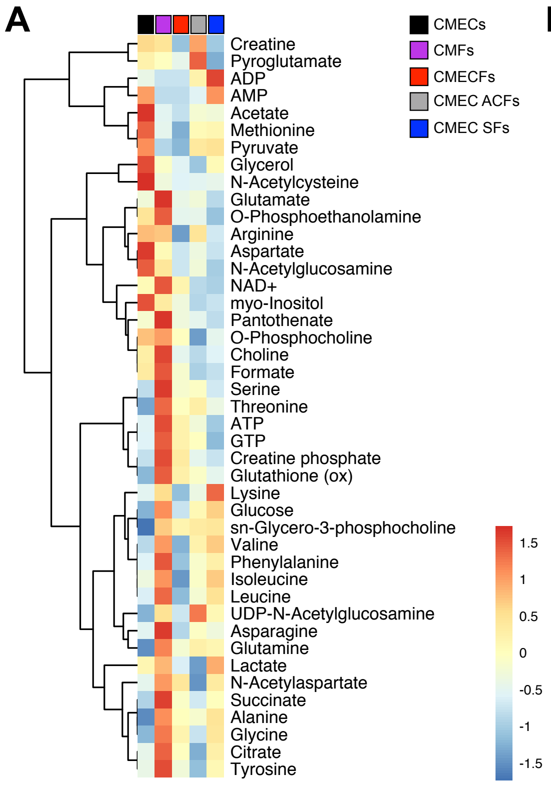

B

	CMECs	CMFs	CMECFs	CMEC ACFs	CMEC SFs
**Lactate**	0.00522	0.01161	0.01235	0.00788	0.01347
**Alanine**	0.00183	0.00182	0.00162	0.00132	0.00249
**Pyro-glutamate**	0.00212	0.00097	0.00090	0.00108	0.00110
**D-glucose**	-0.00038	-0.00898	-0.00803	-0.00764	-0.01375
**Pyruvate**	-0.00094	-0.00080	-0.00056	-0.00121	-0.00090
**Arginine**	-0.00118	-0.00011	-0.00012	0.00000	0.00023
**Glutamine**	0.00142	-0.00004	-0.00020	-0.00030	0.00017
**Proline**	-0.00057	-0.00042	0.00000	-0.00016	-0.00007
**Acetate**	0.00110	-0.00011	0.00027	0.00008	0.00009

Figure S5 (related to Figure 5). Tri-cellular crosstalk between cardiac cell types is essential for metabolic maturation of hiPSC-CMs in microtissues. (A) Heat-map of intracellular metabolites (standardized values) measured in the 5 MT groups. (B) Net release (positive) or uptake (negative) of metabolites to- or from the medium, respectively. Quantities are expressed as µmol, corrected for the total protein mass per sample (µg/ml).

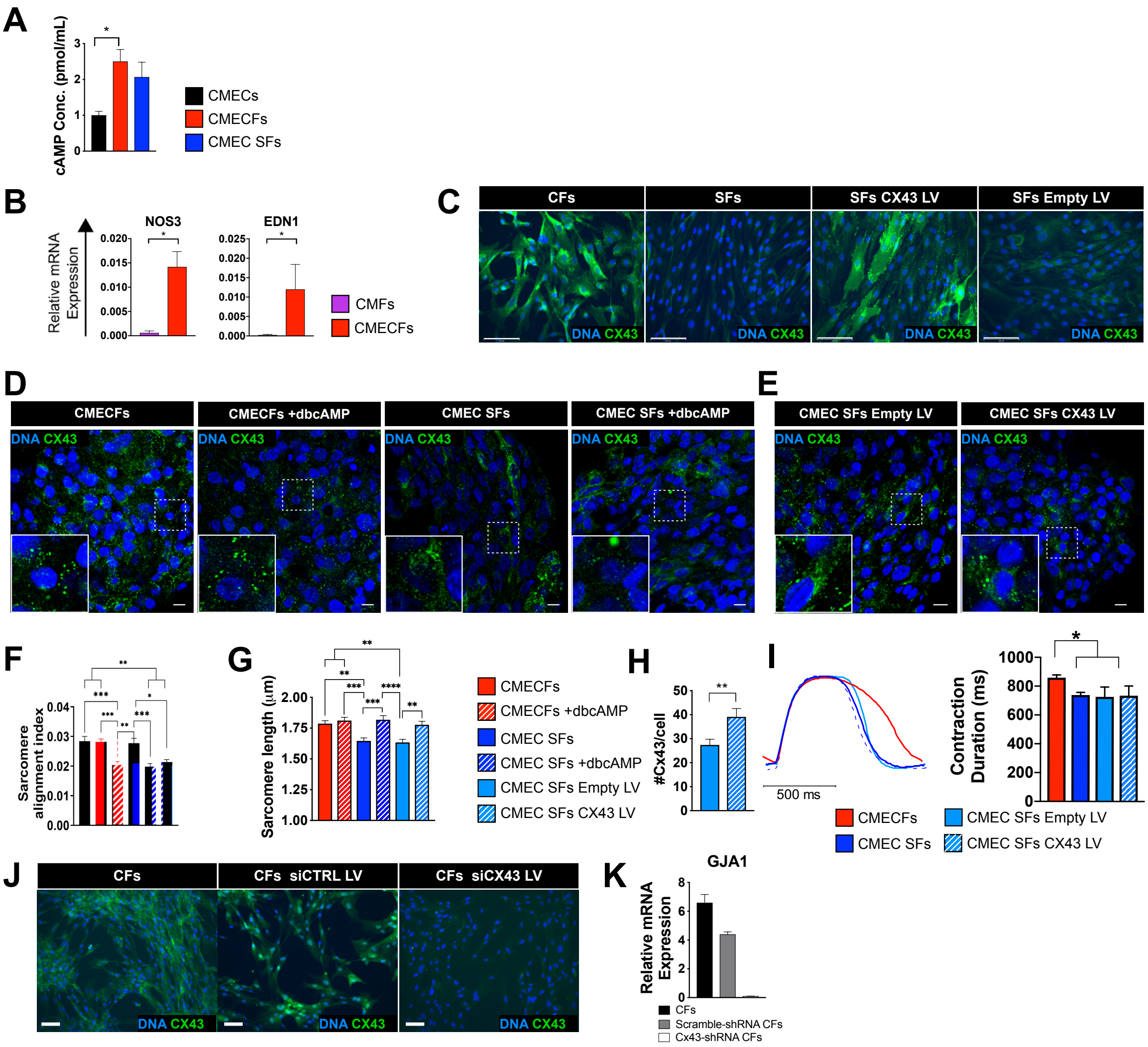

Figure S6 (related to Figure 6). Mechanisms underlying hiPSC-CM maturation in microtissues with cardiac fibroblasts. (A) cAMP concentration (pmol/mL) measured in MTs. N=4, independent MT batches per group, *P<0.05. Friedman test with Dunn's multiple comparisons test. Data are shown as mean ± SEM. (B) qPCR analysis for *NOS3* and *EDN1* genes in CMFs and CMECFs. N>3, independent MT batches; *P<0.05. Student's t-test. Data were normalized to *RPL37A* and are shown as mean ± SEM. (C) Immunofluorescence analysis of CX43 (green) in non-transduced CTRL2 hiPSC-CFs (CFs), CTRL2 primary dermal/skin fibroblasts (SFs), CTRL2 CX43 LV transduced SFs (SFs CX43 LV), CTRL2 Control LV transduced SFs (SFs Empty LV). Nuclei stained with DAPI (blue). Scale bar: 100 µm. (D, E) Representative immunofluorescence images corresponding to those in Figure 6F and 6G) of CX43 (green) alone, to illustrate CX43 distribution in MTs from CTRL1 hiPSCs, either untreated (CMECFs, CMEC SFs) or treated for 7-days with dbcAMP (CMECFs +dbcAMP, CMEC SFs +dbcAMP) (D), and MTs from CTRL1 hiPSCs containing either SFs transduced with control lentivirus (LV) (CMEC SFs Empty LV) or SFs transduced with CX43 lentivirus (LV) (CMEC SFs CX43 LV) (E). Nuclei stained with DAPI (blue). Scale bar: 10 µm. Insets are magnifications of the framed areas and highlight CX43 distribution. (F, G) Quantification of sarcomere organization (sarcomere alignment index) (F) and sarcomere length (G) from immunofluorescence analysis of MTs from CTRL2 hiPSCs (CMECFs and CMEC SFs) and treated for 7-days with dbcAMP (CMECFs +dbcAMP and CMEC SFs +dbcAMP), MTs from CTRL2 hiPSC-CMs and ECs with CTRL2 primary dermal/skin fibroblasts transduced with control lentivirus (LV) (CMEC SFs Empty LV) or CTRL2 primary dermal/skin fibroblasts transduced with CX43 lentivirus (LV) (CMEC SFs CX43 LV). N=30, different areas from 3 MTs per group, *P<0.01, **P<0.005, ***<P=0.0005, ****P<0.0001. One-way ANOVA with Tukey's multiple comparisons test. All data shown as mean ± SEM. (H) Quantification of CX43 per cell in MTs. **P<0.01. Data shown as mean ± SEM. N=15. Student's t-test. (I) Representative contraction traces (left, normalized amplitude is shown) and bar graph of mean contraction duration (right panel) in MTs paced at 1Hz. N=17, MTs per group. *P<0.0001. One-way ANOVA with Fisher's LSD test. Data shown as mean ± SEM. (J) Immunofluorescence analysis of CX43 (green) in non-transduced CTRL1 hiPSC-CFs (CFs), scrambled shRNA CTRL1 hiPSC-CFs and CX43-shRNA CTRL1 hiPSC-CFs. Nuclei stained with DAPI (blue). Scale bar: 100 µm. (K) qPCR analysis for *GJA1* (encoding for CX43) in non-transduced CTRL1 hiPSC-CFs (CFs), scrambled shRNA CTRL1 hiPSC-CFs and CX43-shRNA CTRL1 hiPSC-CFs. Values are normalized to *HARP* and *RPL37A*. N=2, independent transductions. Data shown as mean ± SD.

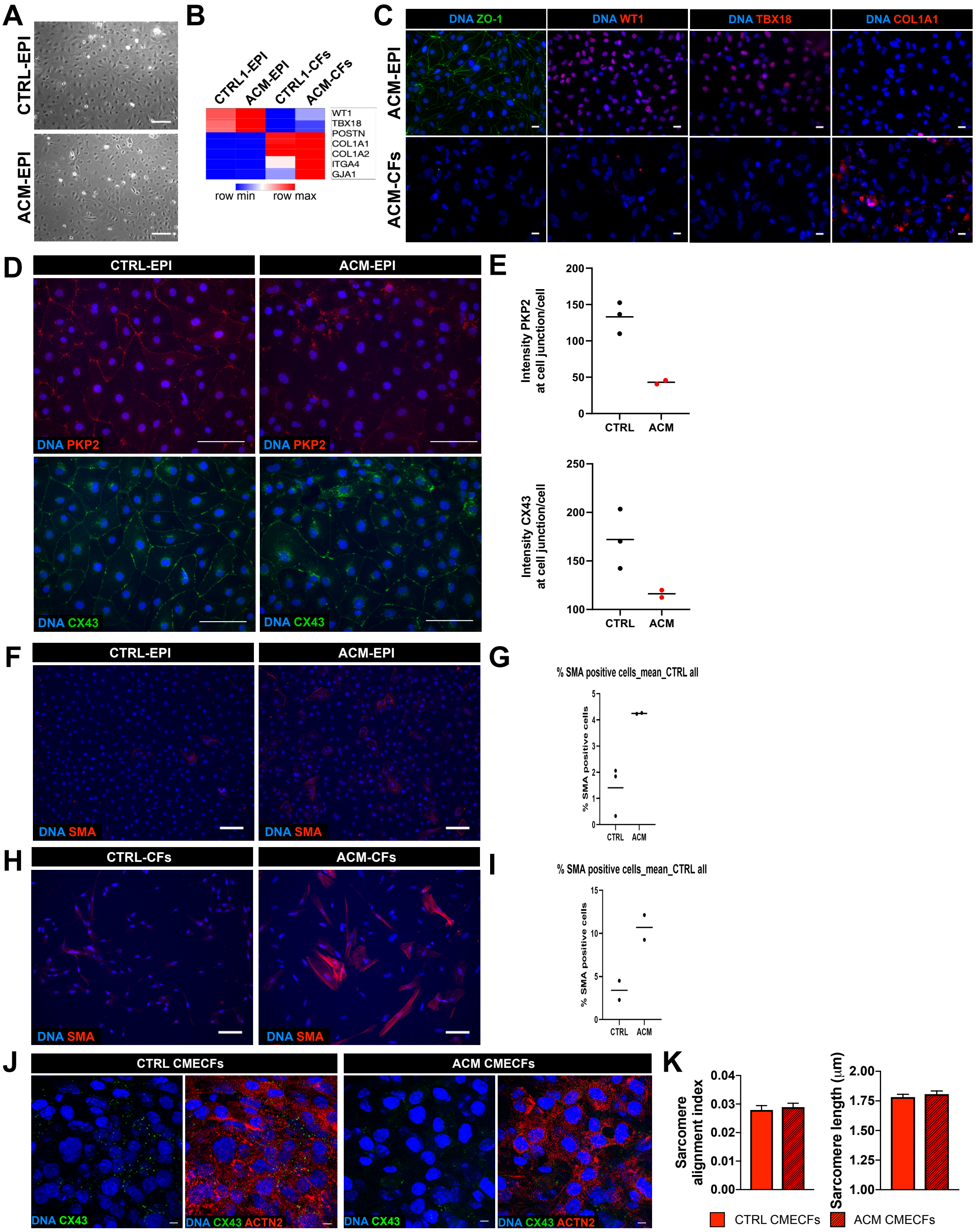

A
CTRL-EPI
ACM-EPI
B
CTRL1-EPI
ACM-EPI
CTRL1-CFs
ACM-CFs
WT1
TBX18
POSTN
COL1A1
COL1A2
ITGA4
GJA1
row min
row max
C
DNA ZO-1
DNA WT1
DNA TBX18
DNA COL1A1
ACM-EPI
ACM-CFs
D
CTRL-EPI
ACM-EPI
DNA PKP2
DNA CX43
E
Intensity PKP2 at cell junction/cell
Intensity CX43 at cell junction/cell
CTRL
ACM
F
CTRL-EPI
ACM-EPI
DNA SMA
G
% SMA positive cells_mean_CTRL all
% SMA positive cells
H
CTRL-CFs
ACM-CFs
DNA SMA
I
% SMA positive cells_mean_CTRL all
% SMA positive cells
J
CTRL CMECFs
ACM CMECFs
DNA CX43
DNA CX43 ACTN2
K
Sarcomere alignment index
Sarcomere length (μm)
CTRL CMECFs
ACM CMECFs

Figure S7 (related to Figure 7). Microtissues as model of Arrhythmogenic Cardiomyopathy (ACM). (A) Representative bright field images of CTRL- and ACM-EPI. Scale bar: 100 µm. (B) Heat-map showing qPCR expression for fibroblast- (*ITGA4, COL1A2, COL1A1, POSTN*) and EPI- (*WT1, TBX18*) genes in hiPSC-EPI and -CFs from CTRL1 and ACM lines. Values were normalized to *RPL37A*. N>3 independent differentiations. (C) Representative immunofluorescence images for ZO-1 (green) and WT1, TBX18, COL1A1 (red) in ACM hiPSC-EPI and ACM hiPSC-CFs. Nuclei stained with DAPI (blue). Scale bar: 20 µm. (D) Immunofluorescence analysis of PKP2 (red, upper panel) and CX43 (green, lower panel) in hiPSC-EPI from CTRL1 hiPSCs (CTRL-EPI) and ACM hiPSCs (ACM-EPI). Nuclei stained with DAPI (blue). Scale bar: 100 µm. (E) Quantification of PKP2 (upper panel) and CX43 (lower panel) intensity at the cell-cell junctions in CTRL-EPI and ACM-EPI. (F) Immunofluorescence analysis of SMA (red) in CTRL-EPI and ACM-EPI. Nuclei stained with DAPI (blue). Scale bar: 100 µm. (G) Quantification of SMA-positive cells in CTRL- and ACM-EPI. CTRL-EPI, N=3 (two independent differentiations from CTRL1 and one from CTRL2); ACM-EPI, N=2 (two independent differentiations). (H) Immunofluorescence analysis of SMA (red) in hiPSC-CFs from CTRL1 hiPSCs (CTRL-CFs) and ACM hiPSCs (ACM-CFs). Nuclei stained with DAPI (blue). Scale bar: 100 µm. (I) Quantification of SMA positive cells in CTRL- and ACM-CFs. CTRL-CFs, N=2 (two independent differentiations from CTRL1 and CTRL2 hiPSCs); ACM-CFs, N=2 (two independent differentiations from ACM hiPSCs). (J) Immunofluorescence analysis of CX43 (green) and cardiac sarcomeric protein ACTN2 (red) in CTRL CMECFs or ACM CMECFs. Nuclei stained with DAPI (blue). Scale bar: 10 µm. (K) Quantification of sarcomere organization (sarcomere alignment index, left) and sarcomere length (right) in CTRL CMECFs and ACM CMECFs. N=30, different areas from 3 MTs per group.

Table S6. List of oligonucleotides used for qPCR.

Oligonucleotides	SOURCE	IDENTIFIER
**Primer sequences for qPCR**		
RPL37A Forward: GTGGTTCCTGCATGAAGACAGTG	Zhang et al. 2014	N/A
RPL37A Reverse: TTCTGATGGCGGACTTTACCG	Zhang et al. 2014	N/A
HARP Forward: CACCATTGAAATCCTGAGTGATGT	Devalla et al. 2015	N/A
HARP Reverse: TGACCAGCCCAAAGGAGAAG	Devalla et al. 2015	N/A
WT1 Forward: TATTCTGTATTGGGCTCCGC	Guadix et al. 2017	N/A
WT1 Reverse: CAGCTTGAATGCATGACCTG	Guadix et al. 2017	N/A
TBX18 Forward: TTGCTAAAGGCTTCCGAGAC	Guadix et al. 2017	N/A
TBX18 Reverse: AGGTGGAGGAACTTGCATTG	Guadix et al. 2017	N/A
GJA1 Forward: TGGTAAGGTGAAAATGCGAGG	PrimerBank	N/A
GJA1 Reverse: GCACTCAAGCTGAATCCATAGAT	PrimerBank	N/A
ITGA4 Forward: AGCCCTAATGGAGAACCTTGT	PrimerBank	N/A
ITGA4 Reverse: CCAGTGGGGAGCTTATTTTCAT	PrimerBank	N/A
COL1A1 Forward: GAGGGCCAAGACGAAGACATC	PrimerBank	N/A
COL1A1 Reverse: CAGATCACGTCATCGCACAAC	PrimerBank	N/A
COL1A2 Forward: GAGCGGTAACAAGGGTGAGC	PrimerBank	N/A
COL1A2 Reverse: CTTCCCCATTAGGGCCTCTC	PrimerBank	N/A
POSTN Forward: CTCATAGTCGTATCAGGGGTCG	PrimerBank	N/A
POSTN Reverse: ACACAGTCGTTTTCTGTCCAC	PrimerBank	N/A
NOS3 Forward: TGATGGCGAAGCGAGTGAAG	PrimerBank	N/A
NOS3 Reverse: ACTCATCCATACACAGGACCC	PrimerBank	N/A
EDN1 Forward: AGAGTGTGTCTACTTCTGCCA	PrimerBank	N/A
EDN1 Reverse: CTTCCAAGTCCATACGGAACAA	PrimerBank	N/A

PrimerBank: https://pga.mgh.harvard.edu/primerbank/
