# Supplementary material for: Human-iPSC-Derived Cardiac Stromal Cells Enhance Maturation in 3D Cardiac Microtissues and Reveal Non-cardiomyocyte Contributions to Heart Disease
Source: Cell Stem Cell. 2020 Jun 4;26(6):862–879.e11. doi: 10.1016/j.stem.2020.05.004 (PMC7284308; doi:10.1016/j.stem.2020.05.004)
Supplement: Document S2. Article plus Supplemental Information [file mmc9.pdf]

Cell Stem Cell

Article

Human-iPSC-Derived Cardiac Stromal Cells Enhance Maturation in 3D Cardiac Microtissues and Reveal Non-cardiomyocyte Contributions to Heart Disease

Graphical Abstract

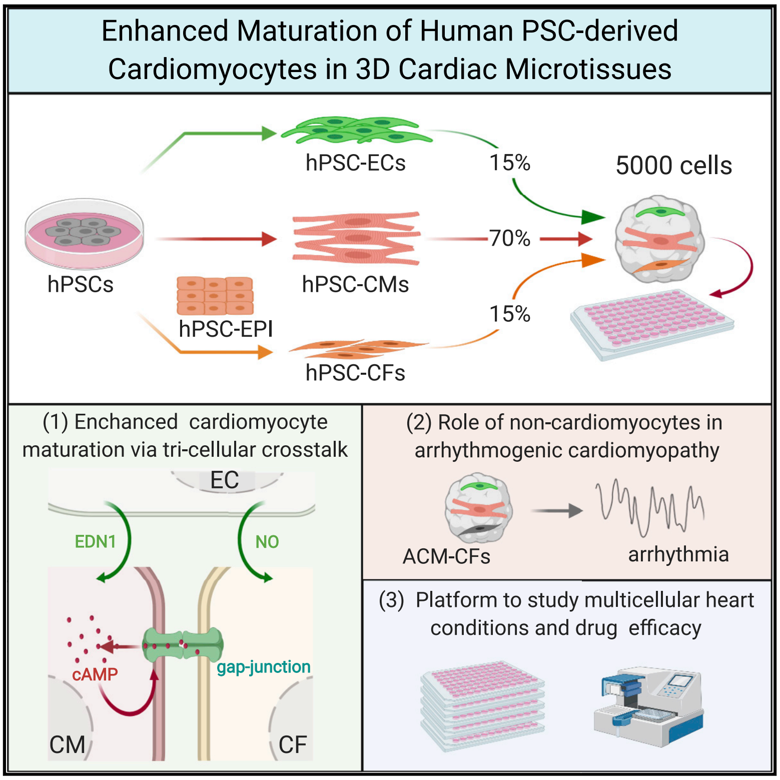

Highlights

- Cardiac fibroblasts and endothelial cells induce hiPSC-cardiomyocyte maturation
- CX43 gap junctions form between cardiac fibroblasts and cardiomyocytes
- cAMP-pathway activation contributes to hiPSC-cardiomyocyte maturation
- Patient-derived hiPSC-cardiac fibroblasts cause arrhythmia in microtissues

Authors

Elisa Giacomelli, Viviana Meraviglia, Giulia Campostrini, ..., Valeria V. Orlova, Milena Bellin, Christine L. Mummery

Correspondence

v.orlova@lumc.nl (V.V.O.),
m.bellin@lumc.nl (M.B.),
c.l.mummery@lumc.nl (C.L.M.)

In Brief

Orlova, Bellin, Mummery, and colleagues combined three hiPSC-derived cardiac cell types in 3D microtissues. Cardiomyocytes matured structurally and functionally. Replacing healthy hiPSC-cardiac fibroblasts with patient fibroblasts recapitulated aspects of arrhythmogenic cardiomyopathy. Single-cell transcriptomics, electrophysiology, metabolomics, and ultrastructural analysis revealed roles for CX43 gap junctions and cAMP signaling in the tri-cell-type dialog.

Giacomelli et al., 2020, Cell Stem Cell *26*, 862–879
June 4, 2020 © 2020 The Author(s). Published by Elsevier Inc.
https://doi.org/10.1016/j.stem.2020.05.004

CellPress

Cell Press OPEN ACCESS

Cell Stem Cell

Article

Human-iPSC-Derived Cardiac Stromal Cells Enhance Maturation in 3D Cardiac Microtissues and Reveal Non-cardiomyocyte Contributions to Heart Disease

Elisa Giacomelli,[1,17] Viviana Meraviglia,[1,17] Giulia Campostrini,[1,17] Amy Cochrane,[1] Xu Cao,[1] Ruben W.J. van Helden,[1] Ana Krotenberg Garcia,[1] Maria Mircea,[2] Sarantos Kostidis,[3] Richard P. Davis,[1] Berend J. van Meer,[1] Carolina R. Jost,[4] Abraham J. Koster,[4] Hailiang Mei,[5] David G. Míguez,[6] Aat A. Mulder,[4] Mario Ledesma-Terrón,[6] Giulio Pompilio,[7,8] Luca Sala,[1,15] Daniela C.F. Salvatori,[9,16] Roderick C. Slieker,[4,10] Elena Sommariva,[7] Antoine A.F. de Vries,[11] Martin Giera,[3] Stefan Semrau,[2] Leon G.J. Tertoolen,[1] Valeria V. Orlova,[1,18,*] Milena Bellin,[1,12,13,18,*] and Christine L. Mummery[1,14,18,19,*]

[1]Department of Anatomy and Embryology, Leiden University Medical Center, 2333 Leiden, the Netherlands
[2]Leiden Institute of Physics, Leiden University, 2333 Leiden, the Netherlands
[3]Center for Proteomics and Metabolomics, Leiden University Medical Center, 2333 Leiden, the Netherlands
[4]Department of Cell and Chemical Biology, Leiden University Medical Center, 2333 Leiden, the Netherlands
[5]Sequencing Analysis Support Core, Leiden University Medical Center, 2333 Leiden, the Netherlands
[6]Centro de Biologia Molecular Severo Ochoa, Departamento de Física de la Materia Condensada, Instituto Nicolas Cabrera and Condensed Matter Physics Center (IFIMAC), Universidad Autónoma de Madrid, 28049 Madrid, Spain
[7]Vascular Biology and Regenerative Medicine Unit, Centro Cardiologico Monzino IRCCS, 20138 Milan, Italy
[8]Department of Clinical Sciences and Community Health, Università degli Studi di Milano, 20122 Milan, Italy
[9]Central Laboratory Animal Facility, Leiden University Medical Center, 2333 Leiden, the Netherlands
[10]Department of Epidemiology and Biostatistics, Amsterdam Public Health Institute, VU University Medical Center, 1007 Amsterdam, the Netherlands
[11]Department of Cardiology, Leiden University Medical Center, 2333 Leiden, the Netherlands
[12]Department of Biology, University of Padua, 35121 Padua, Italy
[13]Veneto Institute of Molecular Medicine, 35129 Padua, Italy
[14]Department of Applied Stem Cell Technologies, University of Twente, 7500 Enschede, the Netherlands
[15]Present address: Istituto Auxologico Italiano, IRCCS, Center for Cardiac Arrhythmias of Genetic Origin and Laboratory of Cardiovascular Genetics, 20095 Cusano Milanino, Italy
[16]Present address: Department of Pathobiology, Anatomy and Physiology Division, Faculty of Veterinary Medicine, Utrecht University, 3584 Utrecht, the Netherlands
[17]These authors contributed equally
[18]Senior author
[19]Lead Contact
*Correspondence: v.orlova@lumc.nl (V.V.O.), m.bellin@lumc.nl (M.B.), c.l.mummery@lumc.nl (C.L.M.)
https://doi.org/10.1016/j.stem.2020.05.004

SUMMARY

Cardiomyocytes (CMs) from human induced pluripotent stem cells (hiPSCs) are functionally immature, but this is improved by incorporation into engineered tissues or forced contraction. Here, we showed that tri-cellular combinations of hiPSC-derived CMs, cardiac fibroblasts (CFs), and cardiac endothelial cells also enhance maturation in easily constructed, scaffold-free, three-dimensional microtissues (MTs). hiPSC-CMs in MTs with CFs showed improved sarcomeric structures with T-tubules, enhanced contractility, and mitochondrial respiration and were electrophysiologically more mature than MTs without CFs. Interactions mediating maturation included coupling between hiPSC-CMs and CFs through connexin 43 (CX43) gap junctions and increased intracellular cyclic AMP (cAMP). Scaled production of thousands of hiPSC-MTs was highly reproducible across lines and differentiated cell batches. MTs containing healthy-control hiPSC-CMs but hiPSC-CFs from patients with arrhythmogenic cardiomyopathy strikingly recapitulated features of the disease. Our MT model is thus a simple and versatile platform for modeling multicellular cardiac diseases that will facilitate industry and academic engagement in high-throughput molecular screening.

INTRODUCTION

Dialogue between stromal, vascular, and tissue-specific cells is essential for maintaining tissue homeostasis. Aside from providing nutrition, growth factors, extracellular matrix (ECM), and hormones, three-dimensional (3D) biophysical interactions with stromal cells are also necessary to ensure proper organ function. Human induced pluripotent stem cells (hiPSCs) can

 © 2020 The Author(s). Published by Elsevier Inc.
This is an open access article under the CC BY license (http://creativecommons.org/licenses/by/4.0/).

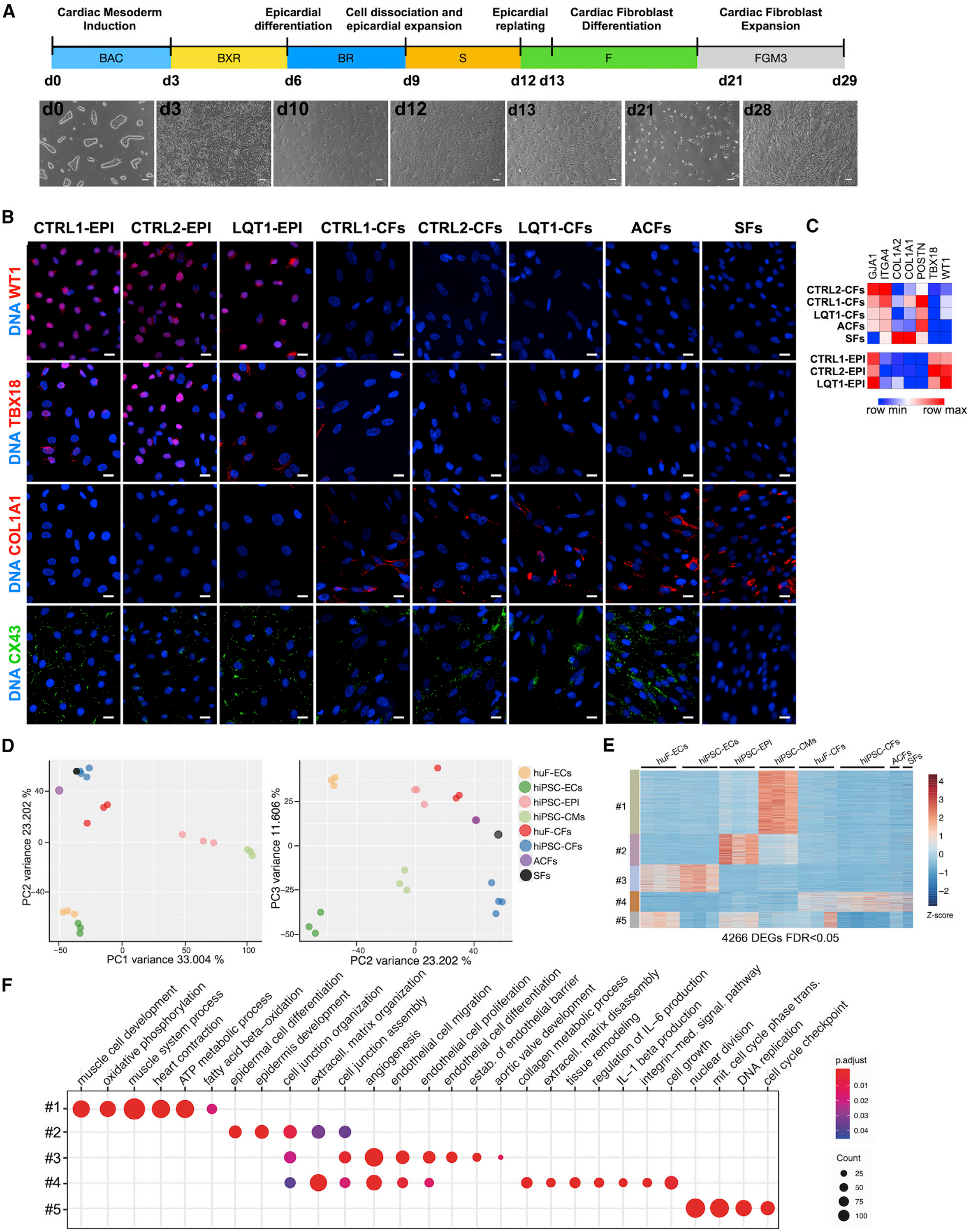

Figure 1. Differentiation, Expansion, and Characterization of hiPSC-Derived Cardiac Fibroblasts

(A) Protocol for hiPSC differentiation into cardiac fibroblasts with bright-field images at indicated times (d, days) for CTRL1-hiPSCs (LUMC0020iCTRL-06). BAC, BMP4 + activin-A + CHIR99021; BXR, BMP4 + XAV939 + retinoic acid; F, FGF2; S, SB431542. Scale bar: 100 μm.

(legend continued on next page)

differentiate into all cell types of the body (Takahashi et al., 2007), capturing the donor genome, but in most cases, differentiated derivatives are immature. We hypothesized that the absence of tissue-specific stromal and vascular cells may contribute to maturation failure and here used the heart as an exemplar to examine the effect of cardiac stromal cells on hiPSC-cardiomyocytes (hiPSC-CMs).

The adult heart contains 30% contractile CMs, the remaining non-CM fraction being cardiac endothelial cells (ECs), vascular stromal cells, and cardiac fibroblasts (CFs) (Pinto et al., 2016). Human embryonic stem cells (hESCs) (Kehat et al., 2001; Mummery et al., 2003) and hiPSCs (Denning et al., 2016) differentiate to CMs, which resemble human fetal rather than adult CMs in their structural, functional, and gene expression profiles (Gupta et al., 2010; van den Berg et al., 2015; Xu et al., 2009; Yang et al., 2014). Nevertheless, they can recapitulate phenotypical traits of many genetic cardiac disorders *in vitro* (Carvajal-Vergara et al., 2010; Caspi et al., 2013; Dell'Era et al., 2015; Dudek et al., 2013; Giacomelli et al., 2017c; Moretti et al., 2010; Te Riele et al., 2017; Siu et al., 2012; Wang et al., 2014) and to some extent predict cardiotoxicity of pharmacological compounds and key pathways in disease (Cross et al., 2015; Sala et al., 2017; van Meer et al., 2019). Relatively mature hiPSC-CMs have only been convincingly observed in 3D scaffold-based cultures or engineered heart tissues (EHTs) *in vitro* (Lemoine et al., 2017; Mannhardt et al., 2016; Ronaldson-Bouchard et al., 2018; Tiburcy et al., 2017) with escalating forced contraction enhancing maturation such that transverse (T-) tubule-like structures become evident (Ronaldson-Bouchard et al., 2018; Tiburcy et al., 2017). T-tubules normally develop postnatally to regulate Ca^{2+} homeostasis, excitation-contraction coupling, and electrical activity of the heart (Brette and Orchard, 2007). However, EHTs require specific expertise, specialized apparatus, gelation substrates, and analysis tools (Mathur et al., 2015) and are thus complex solutions for most academic laboratories and pharma applications. Moreover, monotypic cell configurations do not recapitulate how stromal or vascular cells might affect the behavior of CMs and mediate disease or drug-induced phenotypes.

Here, we addressed these issues by generating multicell-type 3D cardiac microtissues (MTs) starting with just 5,000 cells. We demonstrated previously that hiPSC-ECs derived from cardiac mesoderm affect hiPSC-CMs in 3D MTs (Giacomelli et al., 2017b) and found here that inclusion of hiPSC-CFs further enhanced structural, electrical, mechanical, and metabolic maturation. CFs mainly originate from the epicardium (Tallquist and Molkentin, 2017), the outer epithelium covering the heart. They play crucial roles in cardiac physiology and pathophysiology (Furtado et al., 2016; Kofron et al., 2017; Risebro et al., 2015), contributing to scar tissue formation after myocardial infarction (Rog-Zielinska et al., 2016). They maintain and remodel the ECM, contributing to the integrity and connectivity of the myocardial architecture (Dostal et al., 2015). Although non-excitable themselves, CFs modulate active and passive electrical properties of CMs (Klesen et al., 2018; Kofron et al., 2017; Mahoney et al., 2016; Ongstad and Kohl, 2016). CFs have also been implicated in contractility of hiPSC-CMs in 3D self-assembled (scaffold-free) MTs composed of hiPSC-CMs, primary human cardiac microvasculature ECs, and primary human CFs (Pointon et al., 2017). MTs have to date only been generated using primary stromal cells, which impacts reproducibility and supply. By replacing primary ECs and CFs with hiPSC counterparts, we generated thousands of scaffold-free miniaturized cardiac MTs (CMECFs) containing all cellular components in defined ratios and observed enhanced hiPSC-CM maturation. We demonstrated that CFs, expressing connexin 43 (CX43) gap junction protein, were most effective in supporting hiPSC-CM maturation, and this was mediated by cyclic AMP (cAMP). Skin fibroblasts (SFs), which do not express CX43, and CFs in which CX43 was knocked down were unable to couple to hiPSC-CMs and did not improve maturation. Single-cell (sc) RNA sequencing (RNA-seq) showed that signals from both cardiac ECs and CFs likely contributed to increasing intracellular cAMP in hiPSC-CMs and this was recapitulated by adding dibutyryl (db) cAMP, a cell-permeable analog of cAMP. MTs in which control CFs were replaced by hiPSC CFs carrying a mutation in the desmosomal protein PKP2 that causes arrhythmogenic cardiomyopathy (ACM) strikingly showed CX43 reduction and cardiac arrhythmic behavior despite the CMs being healthy. This illustrates that CFs are crucial in controlling adjacent CM behavior and that CFs were integral contributors to the ACM phenotype.

RESULTS

hiPSC-Derived Epicardial Cells Differentiate into CFs *In Vitro*

Epicardial cells (EPIs) contribute more than 80% of CFs in the heart (Tallquist and Molkentin, 2017). To generate CFs, we first differentiated hiPSC lines into EPIs, as previously described (Guadix et al., 2017; Figure 1A). EPIs emerged with typical epithelial cobblestone-like morphology reaching confluence on day 12 of differentiation (Figure 1A). Immunofluorescence (IF) confirmed nuclear expression of WT1 and TBX18 (Figure 1B). To induce CF differentiation, hiPSC-EPIs were dissociated on day 12 and re-plated in medium with basic fibroblast growth factor (FGF2) (10 ng/mL) for 8 days, refreshing on day 13 and every 2 days thereafter (Figure 1A). Cells became typically mesenchymal (Figure 1A). On day 21, hiPSC-CFs were expanded for an additional 8 days in FGM3 medium. IF confirmed the EPI-

(B) Representative immunofluorescence images of WT1, TBX18, COL1A1 (red), and CX43 (green) of hiPSC-EPIs and hiPSC-CFs from CTRL1, CTRL2, and LQT1 hiPSCs, ACFs, and SFs. Nuclei stained with DAPI (blue). Scale bar: 20 μm.

(C) Heatmap showing qPCR analysis of fibroblast (*GJA1*, *ITGA4*, *COL1A2*, *COL1A1*, and *POSTN*) and EPI (*GJA1*, *WT1*, and *TBX18*) genes in hiPSC-EPIs and hiPSC-CFs from hiPSC lines indicated and ACFs and SFs. Values normalized to *RPL37A*. n = 3.

(D) Principal-component (PC) analysis of hiPSC-CMs, primary human fetal- (huF-ECs) and hiPSC-cardiac ECs (hiPSC-ECs), primary human adult SFs, and hiPSC-EPIs and primary human adult (ACFs), fetal (huF-CFs) and hiPSC-derived CFs (hiPSC-CFs) based on RNA-seq profiles using all genes. Dots represent individual samples; colors different cell types.

(E) Heatmap showing hierarchical clustering of 4,266 DEGs ($P_{FDR} \leq 0.05$) across different cell types showing cell-lineage-specific gene clusters.

(F) GO analysis of cell-lineage-specific gene clusters.

CellPress
OPEN ACCESS

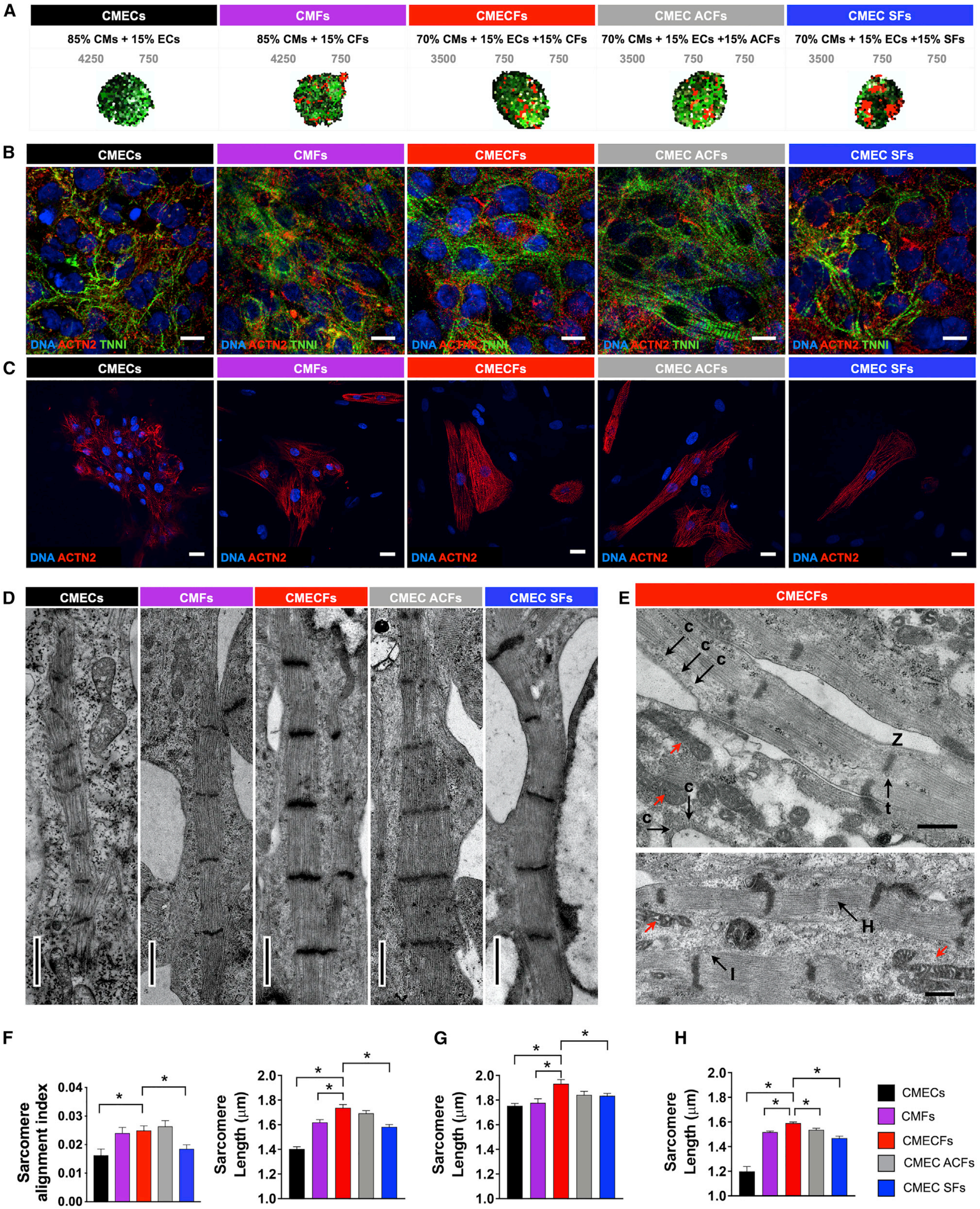

Figure 2. Cardiac Fibroblasts Promote Structural Maturation of hiPSC-CMs in Microtissues

(A) Schematic showing cellular composition of cardiac MT groups. Cell percentages (black) and numbers (gray) are indicated.

(B and C) Representative immunofluorescence images for (B) cardiac sarcomeric proteins TNNI (green) and ACTN2 (red) in MTs (scale bar: 10 μm) and (C) ACTN2 (red) in cells dissociated from MTs (scale bar: 20 μm). Nuclei are stained with DAPI (blue).

(legend continued on next page)

to-CF transition (Figure 1B), with downregulation of WT1 and TBX18 and expression of collagen type alpha1 (COL1A1). By day 29, hiPSC-CFs expressed multiple fibroblast genes (*GJA1*, *ITGA4*, *COL1A1*, *COL1A2*, and *POSTN*) and reduced *WT1* and *TBX18* (Figure 1C). The differentiation protocol was robust and reproducible in three independent hiPSC lines (Figures 1B and 1C); all hiPSC-CFs exhibited similar mRNA and protein marker expression and were more similar to adult CFs (ACFs) than to SFs, which were characterized by negligible expression of the gap junction protein CX43 (*GJA1* gene) and high expression of collagen markers (*COL1A1* and *COL1A2*; Figures 1B and 1C). Principal-component (PC) analysis of whole-transcriptome RNA-seq of hiPSC-CMs, primary human fetal-ECs (huF-ECs) and hiPSC-cardiac ECs (Giacomelli et al., 2017a), SFs, hiPSC-EPIs, and primary human ACFs, huF-CFs, and hiPSC-CFs confirmed striking genome-wide expression correspondence between primary human cardiac cells and their hiPSC-derived equivalents (Figure 1D). Hierarchical clustering of cell-lineage-specific signature genes identified gene clusters upregulated in each of the different cell types (Figure 1E). Gene Ontology (GO) terms for heart and vasculature development, cell junction organization, and collagen metabolic process were associated with hiPSC-CMs, ECs, EPIs, and CFs, respectively (Figure 1F; Table S1). The data thus showed shared cellular identities between primary cardiac cells and their hiPSC-derived equivalents and distinct differences between each cell subtype.

Establishment of 3D Microtissue Model Composed of hiPSC-CMs, hiPSC-ECs, and CFs or Dermal Fibroblasts

Given their distinct identities, we used hiPSC-derived cardiac cells to form 3D MTs containing hiPSC-CMs and hiPSC-cardiac ECs, derived as previously from a common cardiac mesoderm precursor (MT-CMEC; termed here CMECs; Giacomelli et al., 2017a, 2017b), hiPSC-CMs and hiPSC-CFs (CMFs), or, additionally, hiPSC-CMs, hiPSC-ECs, and various types of fibroblasts. These were hiPSC-CFs (CMECFs), ACFs (CMEC ACFs) and adult SFs (CMEC SFs; Figure 2A). MTs were aggregated as spheroids from 5,000 cells in V-bottom 96-well microplates, refreshed every 3 days with vascular endothelial growth factor (VEGF) (CMECs), FGF2 (CMFs), or VEGF and FGF2 (CMECFs, CMEC ACFs, and CMEC SFs) and cultured for 21 days.

We first examined overall morphology and cellular architecture by IF for CM (cardiac troponin I; TNNI), EC (CD31), and fibroblast (COL1A1) markers (Figure S1A) using a computational framework developed in house for 3D semi-automated image processing and segmentation. MTs containing fibroblasts were similar in size and total cell number (Figure S1B), whereas CMECs were smaller, containing fewer cells despite the same input cell number. The percentages of the different cell types (Figure S1C) were comparable among MT groups and reflected the input used for MT formation. Although relatively more $COL1A1^{+}$ cells were found in CMEC SFs (Figure S1C), the percentage of proliferating cells over time remained low in all MT groups with few or no $Ki\text{-}67^{+}COL1A1^{+}$ cells (Figures S1D and S1E). Time-lapse videos of CMEC and CMECF formation showed that CMECFs formed faster and were rapidly more compact than CMECs, suggesting that fibroblasts facilitated aggregation, likely through enhanced cell-cell adhesion (data not shown). Average distance between nuclei indicated that nuclei were indeed more densely packed in MTs containing CFs and ECs than in CMECs (Figure S1F). CM nuclei in CF-containing MTs were larger than in other MT groups (Figure S1G), suggesting that hypertrophy associated with maturation had been initiated. We next examined salient features of adult CM function, namely (ultra)structure, electrophysiology, and mechanical contraction.

CFs Promote Structural Maturation of hiPSC-CMs in Microtissues

Immunostaining for cardiac sarcomeric proteins TNNI and actinin alpha-2 (ACTN2) in MTs revealed better sarcomere development and organization in MTs containing CFs than in CMECs and CMEC SFs from three independent hiPSC lines, as indicated by sarcomere alignment index and length (Figures 2B, 2F, and S2A–S2C). This was confirmed by IF on CMs from dissociated MTs (Figures 2C and 2G) and transmission electron microscopy (TEM) (Figures 2D and 2H). TEM also showed more mature hiPSC-CM ultrastructure in CMECFs, including the presence of caveolae, elongated and enlarged mitochondria with complex cristae, and elongated tubular myofibrils consisting of well-organized sarcomeres with regular Z-lines, I-bands, and H-zones; M-lines and T-tubule-like structures (Eisner et al., 2017) were also visible (Figures 2E and S2D).

Comparison of bulk- and sc-RNA-seq with published datasets showed that CMs in CMECFs clustered closely to adult human CMs, whereas CMs in 2D and in CMECs clustered separately as immature cells (Figure 3A). scRNA-seq analysis of CMs in 2D versus CMECFs confirmed enhanced CM structural maturation in CMECFs, with increased expression of key cardiac sarcomeric genes *TNNT2*, *MYL2*, *MYL3*, *MYL4*, *TNNI1*, *TNNI3*, *DES*, and also *TCAP*, important for sarcomere assembly and T-tubule structure and function in the mammalian heart (Ibrahim et al., 2013; Valle et al., 1997; Figures 3B and S3A–S3D; Table S2).

Furthermore, bulk RNA-seq confirmed that MTs containing hiPSC-CFs were globally more similar to CMEC ACFs than CMEC SFs (Figure 3C). Of note, least biological variability was observed when MTs were entirely hiPSC derived (Figure 3C). Differentially expressed genes (DEGs) were identified between hiPSC-CMs, CMECs, CMECFs, CMEC ACFs, and CMEC SFs (Figure S3E). Unsupervised clustering of DEGs identified genes

(D) Representative transmission electron microscopy (TEM) images showing sarcomeres in different MTs. Scale bar: 1 μm.
(E) TEM images showing caveolae (c), T-tubule like structures (t), Z-lines (Z), I-bands (I), H-zones (H), and elongated mitochondria with complex cristae (red arrows) in CMECFs. Scale bar: 0.5 μm.
(F) Sarcomere organization (sarcomere alignment index; $n > 45$ areas from 3 MTs per group; $^{*}p < 0.05$) and sarcomere length ($n > 47$ areas from 3 MTs per group; $^{*}p < 0.001$) from immunofluorescence analysis in MTs from CTRL1 hiPSCs.
(G) Sarcomere length in hiPSC-CMs dissociated from MTs. $n > 28$ areas from at least 3 independent slides per MT group.
(H) Sarcomere length from TEM in MTs from CTRL1 ($n > 41$ areas from at least 2 independent stitches per group; $^{*}p < 0.05$). Data are mean ± SEM. One-way ANOVA with Dunnett's multiple comparisons test is shown.

CellPress
OPEN ACCESS

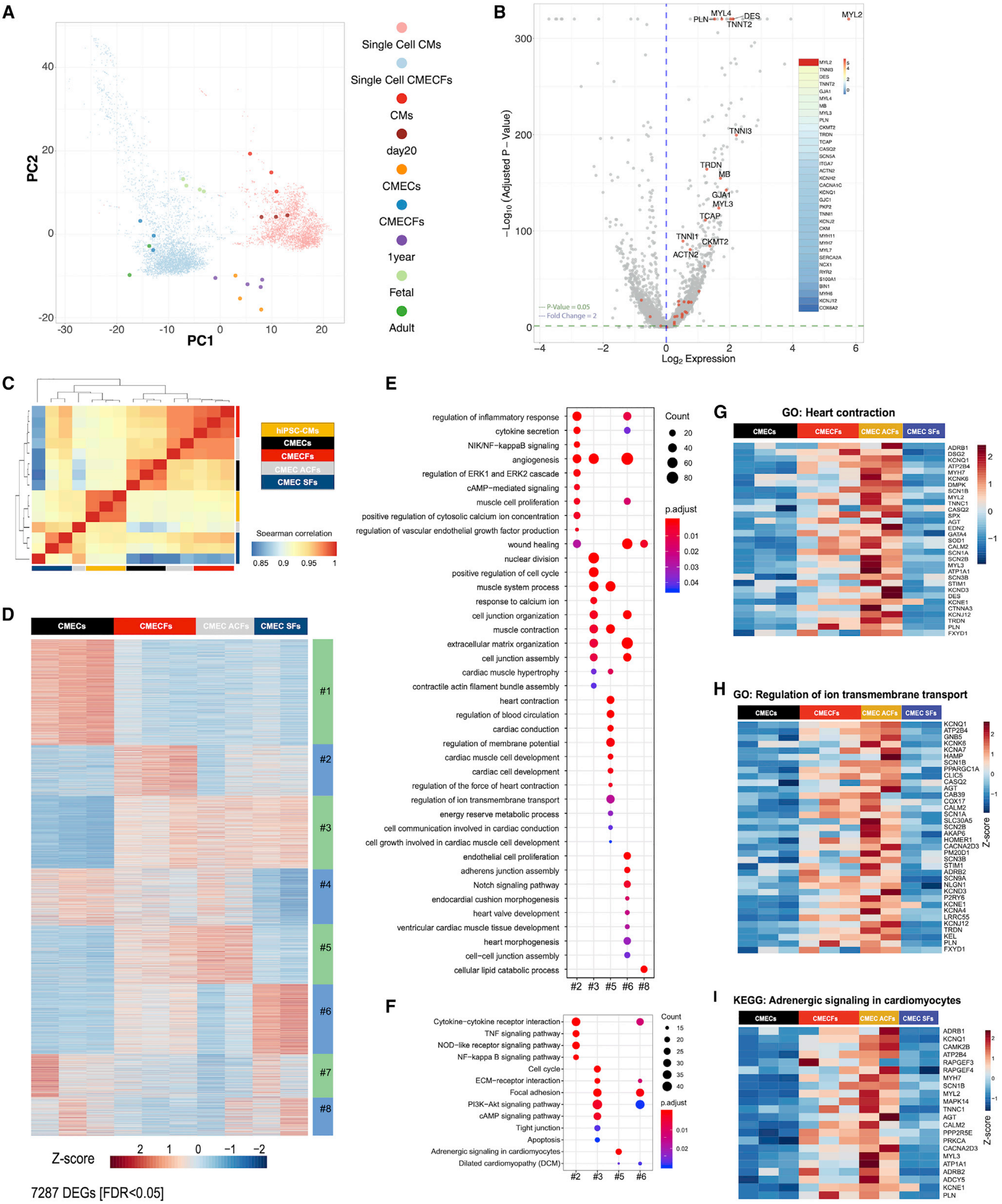

Figure 3. Single-Cell and Bulk Transcriptome Profiling of Microtissues

(A) PC analysis of single-cell (sc) and bulk RNA-seq of hiPSC-CMs at day 20 (single cell CMs; CMs), bulk CMECs (CMECs), and sc and bulk CMECFs (single cell CMECFs; CMECFs) from this study, with bulk hPSC-CMs (day 20), bulk primary human fetal heart (fetal), bulk hPSC-CMs (1 year), and primary human adult heart (adult) from RNA-seq in Kuppusamy et al. (2015); CM cluster). Colors represent different samples.

(legend continued on next page)

most similarly expressed; this resulted in 8 distinct gene clusters (Figure S3F). CMECFs and CMEC ACFs showed common molecular signatures characterized by genes upregulated in cluster 5 (Figure 3D; Table S3), which were not upregulated in CMEC SFs. GO analysis showed enrichment in terms for heart contraction, cardiac conduction, regulation of membrane potential, cardiac muscle cell development, regulation of the force of heart contraction, and regulation of ion transmembrane function (Figure 3E; Table S3). Kyoto Encyclopedia of Genes and Genomes (KEGG) analysis showed that the pathway "adrenergic signaling in cardiomyocytes" was enriched among genes commonly upregulated in CMECFs and CMEC ACFs (cluster 5; Figure 3F; Table S3). Heatmaps of genes associated with GO terms of heart contraction and regulation of ion transmembrane transport and KEGG pathway adrenergic signaling in cardiomyocytes (Figures 3G–3I) showed higher expression of sarcomere proteins, ion channels, and adrenergic receptors in CMECFs and CMEC ACFs compared to CMECs and CMEC SFs.

These findings suggested that both adult- and hiPSC-CFs promote maturation of hiPSC-CMs in MTs such that they display multiple postnatal features.

CFs Promote Electrical Maturation and Enhance Mechanical Contraction of hiPSC-CMs in Microtissues

To determine whether structural maturation was accompanied by electrical maturation, we measured action potentials (APs) in CMs from dissociated MTs using patch-clamp electrophysiology (Figures 4A–4D and S4A). hiPSC-CMs from CMECFs and CMEC ACFs clearly showed similar and improved electrophysiological maturity than hiPSC-CMs from CMECs, CMFs, and CMEC SFs, with many CMs exhibiting fast transient repolarization after the AP peak (referred to as an AP "notch"; Figures 4A and 4B), reflecting expression of the typical transient outward potassium current (I_{to}), in agreement with upregulation of I_{to} genes *KCND3* and *KCNA4* (Figure 3H); moreover, they had more negative resting membrane potentials (RMP), increased AP amplitudes and prolonged AP duration at 90% of the repolarization (APD_{90}) (Figure 4C), although upstroke velocity was increased in all fibroblast-containing MTs compared to CMECs (Figure 4D). These differences between CMECFs and CMECs were confirmed by sharp electrode electrophysiology on whole MTs (Figures S4B and S4C). Incidentally, sharp electrodes detected cells with AP profiles similar to those reported previously as resulting from heterocellular coupling between CMs and CFs (Klesen et al., 2018) through gap junctions in adult native heart tissue (Pellman et al., 2016; Stewart, 1978; Figure S4D).

Immature sarcomere structure is often associated with low contractility. To determine whether sarcomere maturation was accompanied by mechanical maturation, we investigated spontaneous contractile activity of MTs (Sala et al., 2018; Figures 4E–4H). Representative contraction recordings of MTs are shown in Figure 4E. The beat-to-beat intervals were similar in CMECFs and CMEC ACFs and lower than in MTs without ECs or CFs or with SFs (Figure 4F). Contraction duration normalized to beating rate was prolonged (Figure 4G), in agreement with the prolonged APD_{90}, suggesting that both ECs and CFs were necessary to enhance contractility. Contraction amplitude in CMECs, which correlates with force of contraction (Sala et al., 2018), was similar to CMEC SFs and CMFs but lower than in MTs with both CFs and cardiac ECs (Figure 4H). To further analyze contractility, we quantified contraction and relaxation in paced MTs using vector flow analysis. Both parameters were significantly faster in CMECFs than in CMECs, as indicated by the maximum contraction velocity and acceleration measured at increasing pacing frequencies (Figure S4E; Video S1); this suggested greater functional contractility in CMECFs, in line with their improved sarcomere organization. In addition, line integral analysis of the MTs allowed the direction of propagation waves to be depicted (Hayakawa et al., 2012). CMECFs and CMEC ACFs displayed higher contraction velocities and greater coordination of the line integral patterns within each tissue compared to the other MT types (Video S2).

To determine whether improved mechanical performance was accompanied by improved Ca^{2+} handling, we examined Ca^{2+} transients using a Ca^{2+}-sensitive dye in paced MTs (Figures 4I and 4J). MTs with CFs showed different transient profiles (Figure 4I), with increased time to peak and faster decay than CMECs and CMEC SFs (Figure 4J). To investigate sarcoplasmic reticulum (SR) Ca^{2+} content, we examined Ca^{2+} transients induced by caffeine puffs in CMECs and CMECFs. The amplitude of caffeine-induced Ca^{2+} transients was greater in CMECFs than CMECs (Figures S4F and S4G), indicating higher SR Ca^{2+} storage, likely linked to higher expression of key Ca^{2+}-handling protein genes like *CASQ2*, *CALM2*, *PLN*, and *TRDN* (Figures 3G and 3H).

We then determined whether CMECFs could capture negative and positive inotropic responses to known pharmacological agents, verapamil and Bay K-8644, respectively (Figures S4H–S4J). Contraction amplitude decreased upon verapamil treatment in a concentration-dependent manner (Figure S4H), as expected from the block of the L-type calcium channel; this was paralleled by decreased velocity and acceleration of both contraction and relaxation (Figure S4J). By contrast, prolonged relaxation duration was observed upon treatment with the L-type calcium channel agonist Bay K-8644 (Figure S4I).

We conclude that (adult- and hiPSC-derived) CFs promote electrical and mechanical maturation of hiPSC-CMs in 3D MTs, with high reproducibility across lines, batches, and samples

(B) Volcano plot and heatmaps displaying sorted log2 fold-change (FC) and adjusted p values showing expression of selected genes for hiPSC-CMs and CMECFs based on their scRNA-seq profiles. $\log_2 FC > 0$ indicates upregulated genes in the CM cluster of CMECFs versus hiPSC-CMs, whereas $\log_2 FC < 0$ indicates upregulated genes in the CM cluster of hiPSC-CMs versus CMECFs.
(C) Spearman's correlation heatmap of hiPSC-CMs, CMECs, CMECFs, CMEC ACFs, and CMEC SFs based on bulk RNA-seq.
(D) Heatmap showing gene expression in eight gene clusters from the consensus matrix across CMECs, CMECFs, CMEC ACFs, and CMEC SFs.
(E) GO Biological Process terms enriched in gene clusters from consensus matrix ($p_{adj} < 0.05$).
(F) KEGG pathways enriched in gene clusters from consensus matrix ($p_{adj} < 0.05$).
(G–I) Heatmaps showing expression of genes selected from GO: heart contraction (G); GO: regulation of ion transmembrane transport (H); and KEGG: adrenergic signaling in cardiomyocytes (I).

CellPress
OPEN ACCESS

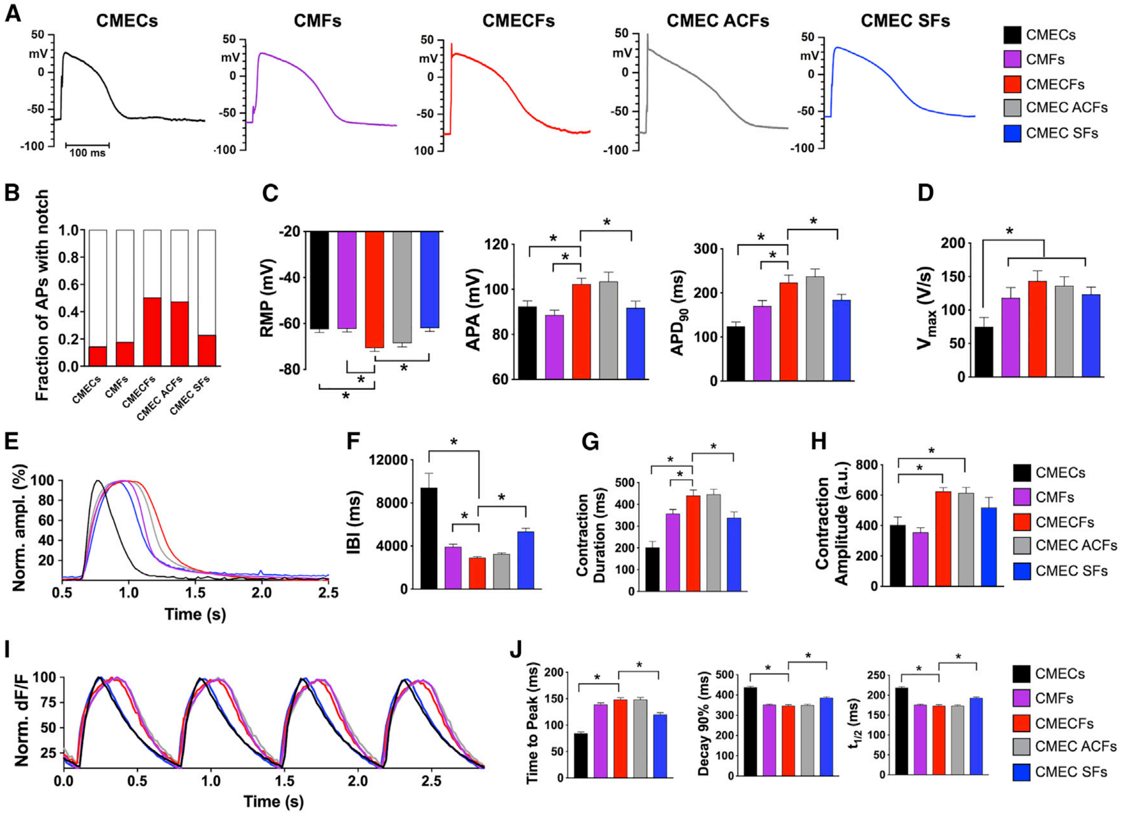

Figure 4. Cardiac Fibroblasts Promote Electrical Maturation and Enhance Mechanical Contraction of hiPSC-CMs in Microtissues

(A) Representative action potential (AP) traces recorded from single hiPSC-CMs dissociated from MT groups indicated, stimulated at 1 Hz.

(B) Bar graph showing the fraction of APs with the I_{to} "notch" (red).

(C and D) APs recorded in single hiPSC-CMs from different MT groups (see A). (C) RMP, resting membrane potential; APA, amplitude; APD_{90}, action potential duration at 90% of repolarization; (D) V_{max}, maximum upstroke velocity in APs measured with dynamic clamp ($n > 18$; single CMs dissociated from 2–5 independent MT batches per group; $^*p < 0.05$).

(E) Representative contraction traces in spontaneously beating MTs. For graphical visualization, amplitude was normalized to each respective maximum amplitude.

(F and G) Inter-beat interval (IBI) (F) and normalized contraction duration (G) in spontaneously beating MTs. $n > 26$; MTs from 3 independent batches per group; $^*p < 0.0001$.

(H) Contraction amplitude in spontaneously beating MTs. a.u., arbitrary units. $n > 7$; MTs; $^*p < 0.05$. One-way ANOVA with Fisher's least significant difference (LSD) test is shown.

(I) Representative Ca^{2+} transients in MTs stimulated at 1.5 Hz.

(J) Ca^{2+} transient parameters (time to peak, peak to 90% decay time, and peak to half decay time) of MTs stimulated at 1.5 Hz. $n > 15$; MTs from 3 independent batches per group. $^*p < 0.0001$. One-way ANOVA with Dunnett's multiple comparisons test is shown. Data in bar graphs are mean ± SEM.

(Figure S4K). Furthermore, tri-cellular crosstalk and the presence of both cardiac ECs and CFs are essential to induce these effects. Finally, CMECFs show drug responses similar to those in EHTs (Mannhardt et al., 2016).

Metabolic Maturation of hiPSC-CMs in Microtissues

To determine whether structural, electrical, and contractile maturation in MTs was accompanied by changes in metabolism, we first examined metabolic gene signatures using scRNA-seq analysis of CMs in CMECFs versus 2D culture. scRNA-seq showed reduction in glycolysis- and increase in beta-oxidation- and tricarboxylic acid cycle (TCA)-associated genes in CMs in CMECFs (summarized schematically in Figure 5A and Table S4). Mitochondrial respiration, glycolytic activity, and the concentration of intracellular metabolites in MTs were then analyzed by Seahorse XF-96 and nuclear magnetic resonance (NMR) spectroscopy (Figures 5B, 5C, S5A, and S5B). CMECFs and CMEC ACFs showed comparable mitochondrial respiration and glycolytic activity that was significantly higher compared to CMECs (Figures 5B and 5C). Intracellular levels of several metabolites in CMECFs and CMEC ACFs were comparable but different from CMECs, such as higher ATP and lower lactate, as well as high uptake of glutamine from the medium, indicating higher mitochondrial respiration (Figures S5A and S5B). By contrast, CMECs were less metabolically active and had a greater preference for glycolysis over mitochondria respiration, as shown by higher intracellular lactate and lower intracellular ATP, as well as lower glucose uptake and low net release of lactate and glutamine (Figure S5A). In addition, a small intracellular pool of lactate (Figure S5A) and high glycolytic activity in

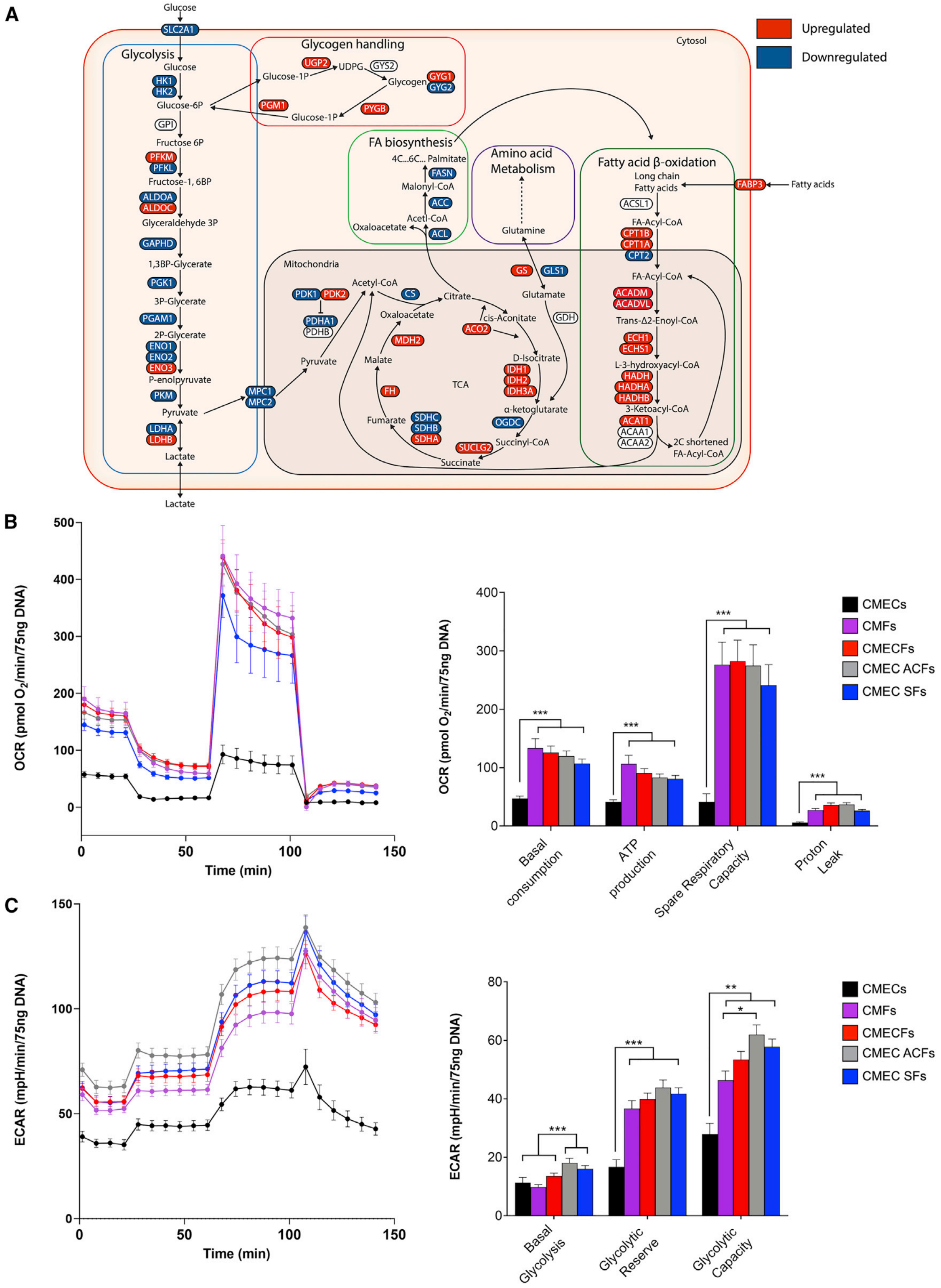

Figure 5. Metabolic Maturation of hiPSC-CMs in Microtissues

(A) Schematic showing metabolic pathways with significantly upregulated (in red) and downregulated (in blue) genes (log2FC; p.adj < 0.05) in the CM cluster of CMECFs versus hiPSC-CMs based on their scRNA-seq profiles. When applicable, heart and muscle isoforms were selected, although other organ isoforms were excluded.

(legend continued on next page)

CMECFs and CMEC ACFs (Figure 5C) suggested that the lactate produced by the ECs and the CFs was shuttled to the CMs for further oxidation. In line with NMR data, scRNA-seq showed that *LDHA* and *LDHB* genes were down- and upregulated, respectively, in CMs in CMECFs (Figure 5A).

The enhanced mitochondrial respiratory capacity in CMECFs and CMCF ACFs indicated that tri-cellular crosstalk between cardiac-specific cells is needed to enhance metabolic maturation in MTs.

Mechanisms Underlying hiPSC-CM Maturation in Microtissues with CFs and ECs Involve CX43 Gap Junctions and cAMP

Because MTs with CFs and ECs most effectively promoted hiPSC-CM maturation, we investigated underlying mechanisms in more detail in CMECs with CFs. Bulk RNA-seq revealed that fibroblast-containing MTs showed common molecular signatures characterized by genes upregulated in cluster 3 and cluster 6 (Figure 3D).

KEGG analysis showed that the cAMP signaling pathway was enriched in cluster 3 and, as mentioned above, adrenergic signaling in CMs , which is linked to cAMP signaling, was enriched specifically in cluster 5 (upregulated in CMECFs and CMEC ACFs; Figure 3F). scRNA-seq also revealed enrichment of the KEGG term for the pathway "adrenergic signaling in CMs" among genes that were upregulated in CMs in CMECFs versus 2D CMs (Table S2). Among these, the CM-specific adenylyl cyclase isoform *ACDY5* was significantly upregulated in CMs from CMECFs versus 2D CMs (Log2FC = 1.78; $P_{FDR} < 0.05$; Table S2). This prompted us to examine whether the upstream regulators of *ADCY5* were also elevated in CMs in MTs. CMs in MTs showed higher expression of the endothelin-1 (*EDN1*) receptor *ENDRA*, which is responsible for induction of *ATF3* expression that in turn would induce expression of *EGR1* (Giraldo et al., 2012), a core transcription factor involved in upregulation of adrenergic receptors (*ADRB1* and *ADRB2*; Iwaki et al., 1990) and *ADCY5* itself (Table S2). Besides adenylyl cyclase, we also found that the soluble guanylyl cyclase isoform *GUCY1A3* was upregulated in CMs and CFs in MTs and highly expressed in CFs compared to CMs in MTs (Log2FC = 2.37; $P_{FDR} < 0.05$; Table S5). *GUCY1A3* is specifically expressed in CFs and not SFs based on published datasets (Furtado et al., 2014). We therefore hypothesized the mechanism illustrated in Figure 6A, proposing that CFs act as a source of cyclic guanosine monophosphate (cGMP) that is shuttled to the CMs via gap junctions and inhibits activity of the phosphodiesterases (PDEs) that convert cAMP to AMP. This might indirectly regulate cAMP levels in CMs. In line with this, MTs showed higher levels of cAMP in CMECFs and CMEC SFs compared to CMECs (Figure S6A). Our scRNA-seq data also supported evidence for the importance of ECs in CM maturation in our MT model, as ECs are the major source of *EDN1* and nitric oxide (NO) (generated by EC-specific *NOS3*), which could activate *ADNRA* and *GUCY1A3* in CMs and CFs (Figure 6B). Importantly, MTs without ECs do not express *EDN1* and *NOS3* (Figure S6B). Bulk RNA-seq also suggested that CMECFs had higher expression of *ADCY5* than CMEC SFs and *GUCY1A3* was significantly higher in CMECFs than CMEC SFs (Figure 6C).

In clusters 3 and 6, GO analysis showed enrichment for the terms "extracellular matrix organization" and "cell junction assembly" (Figure 3E). Importantly, expression of CX43 (*GJA1*) was highly upregulated in scRNA-seq in CMs from CMECFs versus 2D CMs (Log2FC = 1.9; $P_{FDR} < 0.05$; Table S2). CFs dissociated from CMECFs had well-formed CX43 gap junctions with CMs although, by contrast, SFs even in close contact with CMs did not interact via gap junctions (Figure 6D). We therefore hypothesized that CFs coupling to CMs via CX43-mediated gap junctions could promote CM maturation, possibly also via cGMP-cAMP pathways, as described above, further enhancing gap junctions in CMs (Figure 6A).

To test the effect of cAMP levels in CMs, we added its soluble form, dibutyryl cAMP (dbcAMP), to hiPSC-CMs. The resting membrane potential became more negative, the AP amplitude and upstroke velocity higher, and contraction velocity and acceleration faster (Figure 6E), suggesting that persistent activation of the cAMP pathway could contribute to hiPSC-CM maturation.

We investigated this further as follows: (1) we tested whether CMEC SFs could be "rescued" by adding dbcAMP to elevate cAMP levels. This showed hiPSC-CMs in CMEC SFs had better organized and longer sarcomeres, much like those in CMECFs, but there was no further structural maturation in CMECFs with dbcAMP (Figures 6F, 6H, 6I, S6F, and S6G); (2) we tested whether maturation in CMEC SFs could be rescued by ectopic overexpression of CX43 in SFs prior to incorporation in MTs with control hiPSC-CMs and hiPSC-ECs (CMEC SFs CX43 LV; Figures 6G and S6C). Although CX43 was upregulated (Figures S6E and S6H), sarcomeres in CMEC SFs CX43 LV were less organized and shorter than either CMECFs or CMEC SFs with dbcAMP (Figures 6H, 6I, S6F, and S6G). Of note, although CX43 was mainly localized at the cell-cell contacts in CMECFs, it was largely confined to the cytoplasm in CMEC SFs and CMEC SFs CX43 LV. This suggests that proper CX43 localization is important to enhance sarcomere organization (Figures 6F, 6G, S6D, and S6E). Furthermore, overexpression of CX43 in SFs did not rescue contraction in CMEC SFs (Figure S6I); (3) we silenced CX43 in hiPSC-CFs using short hairpin RNA (shRNA) (Figures S6J and S6K) and incorporated these into MTs with control hiPSC-CMs and hiPSC-ECs (siCX43-CMECFs; Figure 6J). Sarcomeres in siCX43-CMECFs were less organized and shorter than CMECFs based on IF (Figures 6L and 6M) and TEM (Figures 6K–6N). Contraction duration was also reduced in siCX43-CMECFs compared to CMECFs, although inter-beat intervals were unaltered (Figure 6O).

Taken together, this demonstrated roles for the cAMP pathway and CX43 in the tri-cellular interactions inducing CM maturation in MTs. These experiments also suggested that there may also be additional mechanistic components missing in SFs, including, for example, GUCY1A3; despite CX43

(B) Traces (left) and bar graphs (right) for oxidative phosphorylation (oxygen consumption rate, OCR) from Seahorse measurements in MTs. n > 52; ***p < 0.001.
(C) Traces (left) and bar graphs (right) for glycolytic acidification (extracellular acidification rate, ECAR) from Seahorse measurements in MTs. n > 44; *p < 0.05; **p < 0.01; ***p < 0.001. All data are shown as mean ± SEM. N indicates MTs from 3–5 independent batches per group. One-way ANOVA with Games-Howell multiple comparison test is shown.

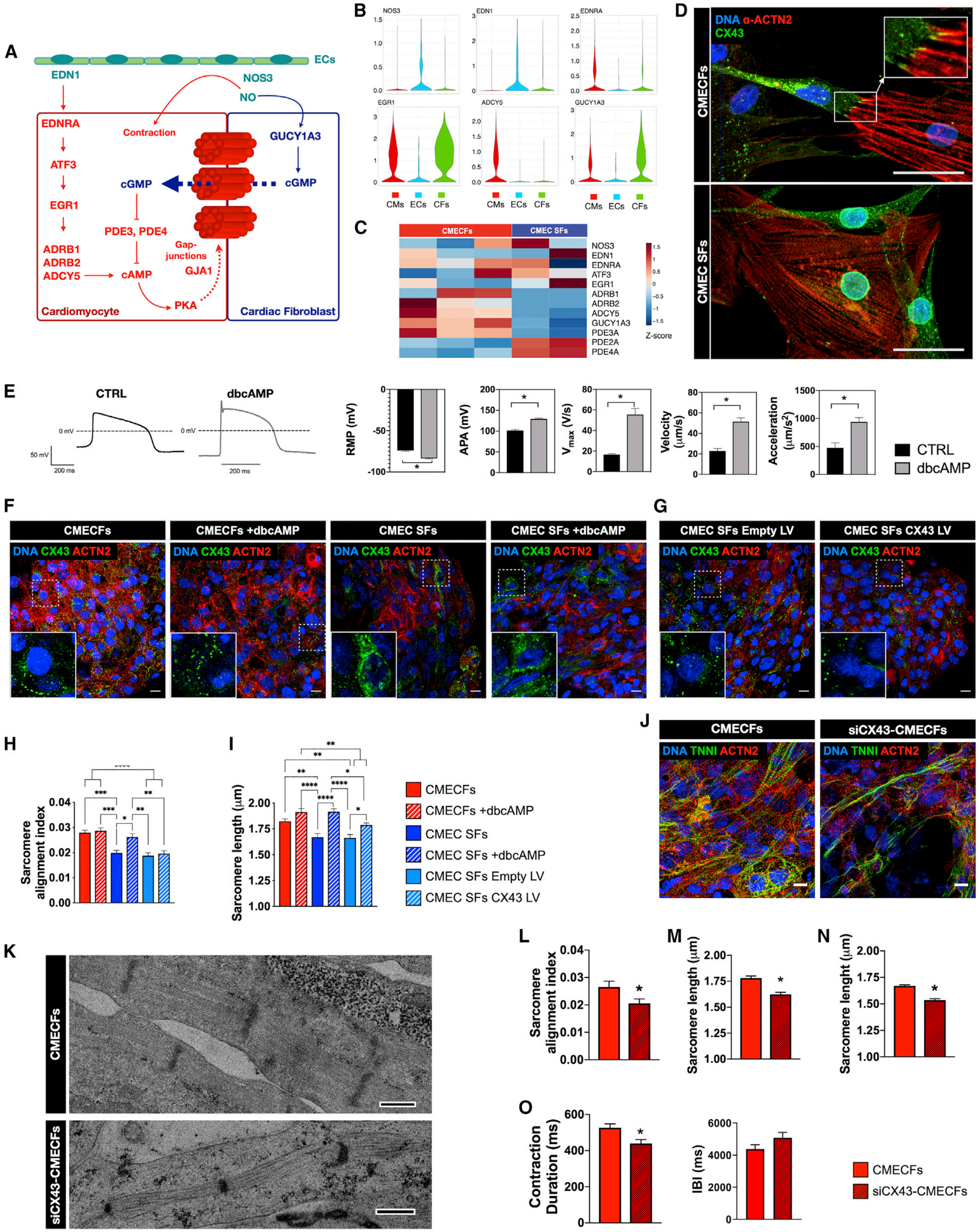

Figure 6. Mechanisms Underlying hiPSC-CM Maturation in Microtissues with CFs and ECs

(A) Proposed mechanism underlying hiPSC-CM maturation in MTs with CFs and ECs: ECs (green) secrete EDN1 that activates β-adrenergic signaling and adenylyl cyclase in CMs (red), increasing intracellular cAMP levels, which can enhance CX43 gap junction formation. ECs also secrete NO that activates cGMP pathway in CFs (blue). cGMP can shuttle to CMs via gap junctions (dotted blue arrow), sustaining cAMP in CMs.

(legend continued on next page)

overexpression, SFs are inferior to CFs as an integral functional component of MTs.

Microtissues Enable Multilineage Cardiac Disease Modeling

CMs are the only affected cells in channelopathies, but other inherited cardiac disorders may not be CM autonomous, with non-myocyte cells in the heart playing active etiological roles in the disease (Blazeski et al., 2019; Sommariva et al., 2017). Arrhythmogenic cardiomyopathy (ACM) is one such rare genetic disorder, predominantly associated with mutations in desmosomal genes and characterized by arrhythmias and fibro-fatty replacement of the myocardium (Lazzarini et al., 2015; Sommariva et al., 2017). A role for CFs in ACM pathophysiology has been recently indicated using primary cells from patients (Sommariva et al., 2016). Here, we used ACM hiPSCs from a patient carrying the heterozygous c.2013delC *PKP2* mutation, which results in a premature stop codon (Figure 7A). We generated CTRL- and ACM-MTs by combining CTRL hiPSC-CMs and ECs with either CTRL hiPSC-CFs or SFs or with ACM hiPSC-CFs or SFs (Figure 7B). Although no differences in morphology (Figure 7C) or epicardial (ZO-1, WT1, and TBX18) and fibroblast (COL1A1) marker expression were detected in hiPSC-ACM-EPIs and ACM-CFs compared with controls (Figures S7A–S7C), ACM-EPIs showed lower PKP2 levels by western blot (Figure 7D) and reduced PKP2 at cell junctions by IF (Figures S7D and S7E), likely due to the *PKP2* mutation. Interestingly, this was paralleled by reduced junctional localization of CX43 in ACM-EPIs (Figures S7D and S7E), in agreement with a role of PKP2 in regulating CX43 trafficking (Agullo-Pascual et al., 2013; Oxford et al., 2007; Sato et al., 2011; Zhang and Shaw, 2014). PKP2 protein expression in hiPSC-CFs was much lower than in hiPSC-EPIs, in agreement with absence of desmosomes in CFs and differences between ACM- and CTRL-CFs were less clearly detected (Figure 7D). Nevertheless, in MTs, we found that CTRL CFs, but not ACM CFs, sustained CX43 expression throughout the microtissue (Figures 7E, 7F, and S7J), although sarcomere organization was not affected in CMs (Figure S7K). Furthermore, MTs with CTRL or ACM SFs did not show any differences in CX43 expression (Figures 7E–7G).

We next characterized the electrical properties of MTs by stimulating them at increasing pacing frequencies. Inclusion of ACM hiPSC-CFs significantly reduced the ability of MTs to respond to high stimulation frequencies ($\geq$2 Hz), which resulted in arrhythmic behavior (Figures 7H and 7I). Arrhythmic behavior can be linked to failure of CMs to properly couple, possibly because of reduced CX43 expression. Of note, this arrhythmic ACM phenotype was not captured in MTs with SFs.

Finally, we noted a higher proportion of cells positive for alpha-smooth muscle actin (SMA) in ACM-EPIs (Figures S7F and S7G) and CFs compared to controls (Figures S7H and S7I). This suggested that (1) ACM EPIs had a higher propensity to undergo epithelial-to-mesenchymal transition (EMT), supporting the concept that epicardial cells are a source of fibro-fatty substitution in the hearts of ACM patients with PKP2 mutations (Lombardi et al., 2009; Matthes et al., 2011) and (2) some ACM-CFs display a myo-fibroblast-like phenotype, which may impact conduction of CMs in MTs (Thompson et al., 2011).

Taken together, these findings provide evidence of non-myocyte contributions to ACM pathogenesis and demonstrate the utility of MTs that are completely hiPSC derived in modeling diseases not autonomous to CMs.

DISCUSSION

In this study, we described a 3D MT system composed of CMs , cardiac ECs , and CFs, the three major cell types of the heart, derived entirely from hiPSCs. MTs were formed from just 5,000 cells by self-aggregation in controlled ratios that remained constant over time. Tri-cellular crosstalk promoted hiPSC-CM maturation specific to CFs. This required close cellular contacts and CX43 gap junctions. In contrast to other 3D systems, such as EHTs (Lemoine et al., 2017; Mannhardt et al., 2016; Ronaldson-Bouchard et al., 2018; Tiburcy et al., 2017), MTs enhanced CM maturation without requiring specialized devices and analysis tools, technical tissue engineering expertise, mechanical load, scaffolds, or complex substrates. The three cardiac cell types for MTs can be derived isogenically from the same hiPSC-derived cardiac mesoderm, in a highly reproducible way

(B) Violin plots showing (log-transformed) expression of *NOS3*, *EDN1*, *EDNRA*, *EGR1*, *ADCY5*, and *GUCY1A3* in CMECFs based on scRNA-seq.
(C) Heatmap showing selected gene expression from bulk RNA-seq of CMECFs and CMEC SFs.
(D) Immunofluorescence analysis of CX43 (green) and ACTN2 (red) in hiPSC-CMs and fibroblasts dissociated from CMECFs and CMEC SFs MTs. White arrows indicate coupling between hiPSC-CFs and hiPSC-CMs. SFs do not couple with hiPSC-CMs. Nuclei are stained with DAPI (blue). Scale bar: 50 μm.
(E) Representative AP traces of untreated (CTRL, black) and 72-h-dbcAMP-treated (dbcAMP, gray) CTRL1 hiPSC-CMs, with quantification of RMP, APA, V_{max}, contraction velocity, and acceleration. n > 10; dissociated cells per group; *$p < 0.001$. Data are mean ± SEM. Student’s t test is shown.
(F and G) Representative immunofluorescence images of CX43 (green) and ACTN2 (red) in MTs from CTRL1 hiPSCs, either untreated (CMECFs, CMEC SFs) or treated for 7 days with dbcAMP (CMECFs +dbcAMP, CMEC SFs +dbcAMP; F) and MTs from CTRL1 hiPSC containing either SFs transduced with control lentivirus (LV) (CMEC SFs empty LV) or lentivirus containing CX43 LV (CMEC SFs CX43 LV; G). Nuclei are stained with DAPI (blue). Scale bar: 10 μm. Insets are magnifications of framed areas to show CX43 distribution.
(H and I) Sarcomere organization (sarcomere alignment index; H) and sarcomere length (I) from immunofluorescence analysis of MTs from CTRL1 hiPSCs. n = 30; areas from 3 MTs per group; **$p < 0.05$; ***$p < 0.005$; ****$p < 0.0001$. Data are mean ± SEM. One-way ANOVA with Tukey’s multiple comparisons test is shown.
(J) Representative immunofluorescence images of cardiac sarcomeric proteins ACTN2 (red) and TNNI (green) in CMECFs generated from CTRL1 CMECFs containing either untreated hiPSC-CFs (CMECFs) or hiPSC-CFs transduced with CX43-shRNA (siCX43-CMECFs). Nuclei are stained with DAPI (blue). Scale bar: 10 μm.
(K) TEM showing sarcomeres in CMECFs and siCX43-CMECFs. Scale bar: 0.5 μm.
(L–N) Quantification of sarcomere organization (sarcomere alignment index; L) and sarcomere length (M) from immunofluorescence analysis in MTs (n = 60; areas from 4 MTs per group; *$p < 0.05$) and of sarcomere length in MTs from TEM (-n; n > 117; areas from 3 independent stitches per group; *$p < 0.0001$). Data are shown as mean ± SEM. Student’s t test is shown.
(O) Contraction duration (left) and IBI (right) measured in spontaneously beating MTs. n > 40; MTs from 3 independent batches per group; *$p < 0.05$. Student’s t test is shown. Data are shown as mean ± SEM.

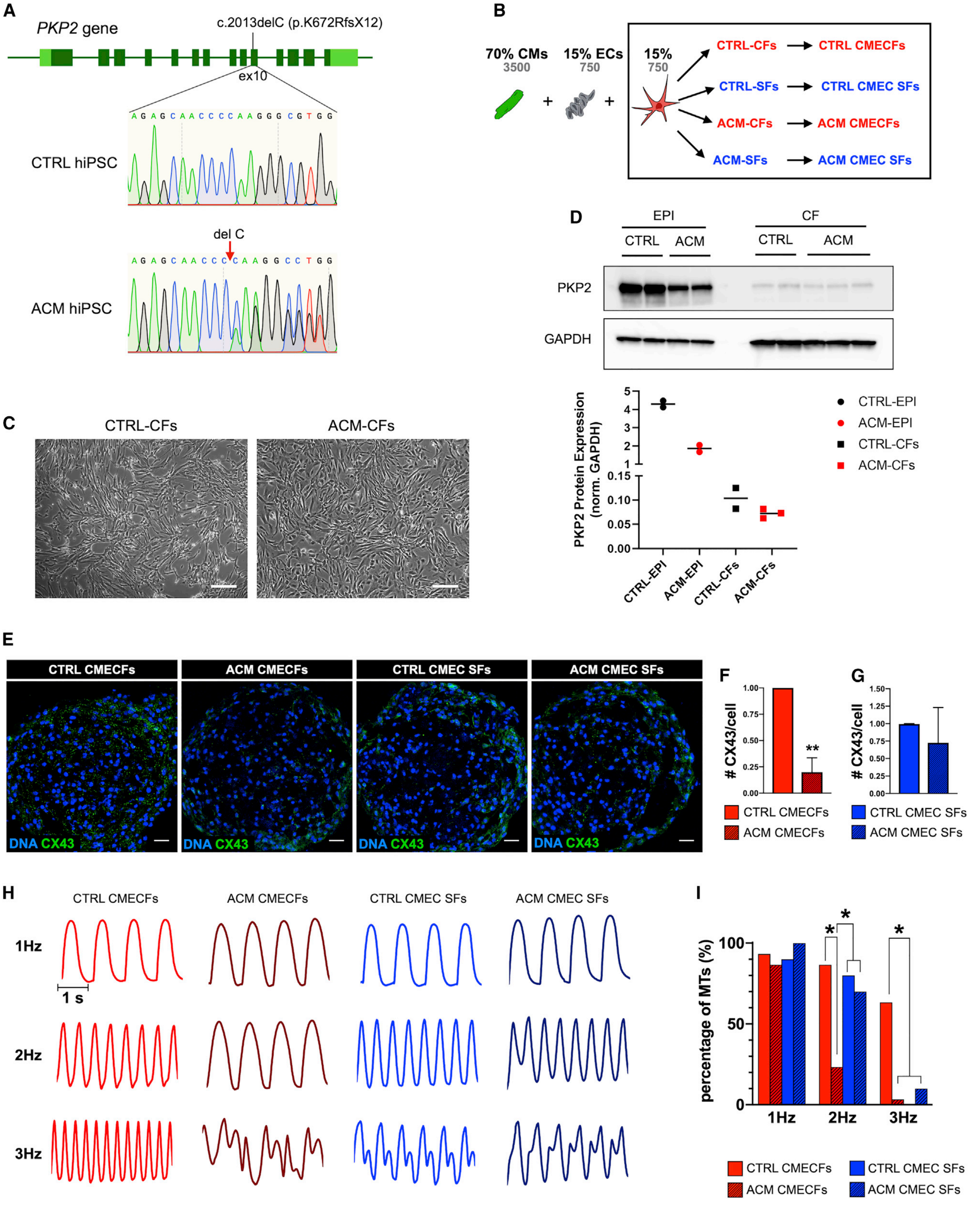

Figure 7. Microtissues as a Model of Arrhythmogenic Cardiomyopathy (ACM)

(A) Sequencing of *PKP2* gene showing heterozygous c.2013delC (p.K672RfsX12) mutation in exon 10 in ACM hiPSCs. *PKP2* sequence of CTRL1 hiPSCs is shown as reference.

(legend continued on next page)

among different hiPSC lines, and can be stock frozen without altering their properties. MT construction was robust and highly reproducible with low sample-to-sample, batch-to-batch, and line-to-line variability across multiple parameters. The results demonstrated our *in vitro* cardiac tissue model is low cost (±0.22€ per MT); amenable to high-throughput production for structural, functional, and electrophysiological analysis; and illustrated the utility of MTs in disease modeling.

Postnatal CMs have longer and more organized sarcomeric structures than fetal and hiPSC-CMs, and these ensure proper force generation. After birth, T-tubules mediate rapid excitation-contraction coupling, electrophysiological properties change due to expression of distinct ion channels, conduction velocities are higher due to subcellular redistribution of CX43 gap junction protein, there are greater intracellular Ca^{2+} stores, ryanodine receptors mediate Ca^{2+} release, and metabolism is dependent on fatty acid oxidation rather than glycolysis (Yang et al., 2014). Here, we showed that many of these features were evident hiPSC-CMs in tri-cellular MTs and CFs outperformed primary SFs. Specifically CMECFs showed improvements in (1) structure: sarcomere length and organization were increased, with ultrastructure characteristics of mature CMs (H zones, I-bands, M-lines, T-tubule-like structures, and elongated mitochondria adjacent to sarcomeres); (2) function: mechanical contraction (increased duration and amplitude) and Ca^{2+} handling were improved and electrophysiology showed more mature AP profiles (hyperpolarized RMP and higher upstroke velocity); and (3) metabolism: mitochondrial respiration capacity was increased although dependence on glycolysis was decreased. Importantly, this broad spectrum of maturation features was only achieved in MTs containing both hiPSC-ECs and CFs (either adult or hiPSC derived), indicating that the tri-cellular crosstalk is essential.

RNA-seq analysis showed that cAMP/β-adrenergic and cell junction assembly pathways were specifically upregulated in CMECFs, but not in MTs with non-cardiac fibroblasts, suggesting their involvement in enhancing maturation. Notably, cAMP levels were higher in CMECFs and dbcAMP increased electrical maturation in hiPSC-CMs even in 2D. Based on our results and published data on signaling in the heart, we propose that one mechanism underlying enhanced maturation in MTs (Figure 6A) involves both ECs and CFs, with increased cAMP levels in CMs positively affecting the assembly of CX43 gap junctions. This notion is supported by our data showing sustained cAMP signaling through exogenous dbcAMP also improved sarcomeric organization in CMEC SFs to the level of CMECFs. Involvement of gap junctions was demonstrated by silencing CX43 in CMECFs using shRNA, which reduced structural organization of sarcomeres. Lack of CX43 in CMECFs also reduced contraction duration but did not affect the beating rate, suggesting that the faster beating rate in CMECFs was not necessary or sufficient for structural maturation but also that CX43 was not required for maintaining the beating rate. CX43 expression as such did not appear to be the sole mechanism for maturation, however, because CX43 overexpression in CMEC SFs only partially rescued structural organization compared to CMECFs or dbcAMP treatment. Of note, CX43 overexpression in SFs resulted mostly in cytoplasmic rather than cell junction CX43 localization. Thus, we identified some key mechanisms in the tri-cellular interactions that enhance CM maturation, but other mechanisms, such as cell-extracellular matrix interactions or paracrine effects, may also play roles. These will likely all be necessary ultimately to obtain fully mature hiPSC-CMs.

One important advantage of our tri-cellular MT system based entirely on hiPSC-derived cells is the opportunity to create patient-specific models of disease that may have multicell-type causes. This was illustrated by our use of hiPSC-CFs derived from an ACM patient carrying a PKP2 mutation. When incorporated into MTs as the only diseased cellular component, ACM-CFs induced arrhythmic behavior in wild-type (WT) CMs. Of note, ACM-CFs were characterized by a higher tendency to assume a myofibroblast-like identity and ACM-MTs showed reduced CX43 expression. Both features could impact electrical conduction of CMs. Arrhythmia is one of the earliest events in ACM patients, preceding fibro-fatty deposition (Gomes et al., 2012); thus, our data showed another role of CFs in ACM pathogenesis. This experiment demonstrates the utility of the MT system in modeling multicellular cardiac disease, as it enabled investigation of interactions among cell types, specifically identification of cellular "culprits" versus "victims" in diseases non-autonomous to CMs.

In conclusion, 3D models of the heart based on hiPSCs are already excellent resources to study differentiation of human heart cells in development and the consequences of heart disease or drugs *in vitro*. The incorporation of multiple cardiac cell types as here serves as an exemplar for MTs of other organs for which either biopsies are not feasible or there is a stromal component to the disease not captured by only including one cell type in a bioassay *in vitro*. We have illustrated the power of being able to create cells from patient-specific lines. As large-scale studies on genetics and corresponding

(B) Generation of CTRL and ACM MTs using CTRL hiPSC-CMs and CTRL hiPSC-ECs combined with either CTRL or ACM hiPSC-CFs or primary CTRL or ACM SFs. Cell percentages (black) and numbers (gray) are indicated at the top.

(C) Representative bright-field images of CTRL- and ACM-CFs. Scale bar: 100 μm.

(D) Western blot for PKP2 in CTRL and ACM hiPSC-EPIs and CFs. CTRL-EPIs were differentiated from two hiPSC lines (CTRL1 and CTRL2), although ACM EPI samples are two independent differentiations from ACM hiPSCs. CTRL and ACM CF samples are two and three independent differentiations from CTRL1 and ACM hiPSCs, respectively. GAPDH was used as loading control. Densitometric analysis is shown in the lower panel.

(E) Immunofluorescence analysis of CX43 (green) in CTRL CMECFs, ACM CMECFs, CTRL CMEC SFs, and ACM CMEC SFs MTs. Nuclei are stained with DAPI (blue). Scale bar: 25 μm.

(F and G) Quantification of CX43 per cell in CTRL and ACM CMECFs (F) and in CTRL and ACM CMEC SFs (G; n = 3; independent MT batches per group; $^{**}p < 0.005$). Data are shown as mean ± SEM, normalized to the respective CTRL. Student's t test is shown.

(H) Representative contraction traces from CTRL and ACM CMECFs and CTRL and ACM CMEC SFs stimulated at 1 Hz, 2 Hz, and 3 Hz.

(I) Percentages of MTs that could be paced at different stimulation frequencies in different MT groups (see legend). n > 10; MTs per group; $^{*}p < 0.05$. Data are shown as mean ± SEM. Chi-square test is shown. All data shown were from CTRL1 and/or CTRL2 hiPSC as a source of hiPSC-CMs, CFs, and primary SFs.

hiPSCs become available (e.g. NIBSC, UK; EBiSC, EU; CIRM, US; and CiRA, Japan), this will yield more "variants of unknown significance," many of which will not only be expressed by CMs. Environmental diseases, such as fibrosis following myocardial infarction and microvascular disease leading to heart failure with preserved ejection fraction, are also multicellular.

Controlled formation of hiPSC-derived MTs with the major cell types of the heart thus represents a valuable platform that regulatory authorities, pharmaceutical companies, and academia can use to understand multicellular heart conditions, identify therapeutic targets, and predict drug efficacy in humans.

STAR★METHODS

Detailed methods are provided in the online version of this paper and include the following:

- KEY RESOURCES TABLE
- RESOURCE AVAILABILITY
  - Lead Contact
  - Materials Availability
  - Data and Code Availability
- EXPERIMENTAL MODEL AND SUBJECT DETAILS
  - Ethics statement
  - hiPSC lines culture
  - Clinical history and genetic phenotype of ACM patient
  - Primary fibroblasts culture
  - Human fetal heart ECs and fibroblasts
- METHOD DETAILS
  - Differentiation of hiPSCs into CMs, cardiac ECs and EPI
  - Differentiation of hiPSC-EPI into CFs
  - Lentiviral transduction of hiPSC-CFs using shRNA-CX43
  - Lentiviral transduction for CX43 overexpression in primary SFs
  - 3D cardiac microtissue (MT) culture
  - OCT cryosections of 3D microtissues
  - Immunofluorescence analysis
  - Transmission Electron Microscopy (TEM)
  - Contraction analysis
  - Sharp Electrode Electrophysiology
  - Patch clamp electrophysiology
  - Calcium analysis
  - Drug preparation
  - Measurement of cAMP in microtissues
  - Oxidative respiration and glycolytic acidification
  - Nuclear Magnetic Resonance spectroscopy (NMR)
  - Gene expression (qPCR)
  - Gene expression (bulk RNA-sequencing)
  - Gene expression (single-cell RNA-sequencing)
  - Western blot
  - Bright field images
- QUANTIFICATION AND STATISTICAL ANALYSIS
  - Quantitative sarcomere analysis by immunostaining
  - Quantitative CX43 analysis by immunostaining
  - Computational framework for quantitative analysis
  - Statistics

SUPPLEMENTAL INFORMATION

Supplemental Information can be found online at https://doi.org/10.1016/j.stem.2020.05.004.

ACKNOWLEDGMENTS

This project was funded by the following grants: European Research Council (ERCAdG 323182 STEMCARDIOVASC); European Community's Seventh Framework Programme (FP7/2007-2013) under grant agreement no. 602423; European Union's Horizon 2020 Research and Innovation Programme under grant agreement no. 668724; Netherlands Organ-on-Chip Initiative, an NWO Gravitation project funded by the Ministry of Education, Culture and Science of the government of the Netherlands (024.003.001); Transnational Research Project on Cardiovascular Diseases (JTC2016_FP-40-021 ACM-HF); the Netherlands Organisation for Health Research and Development ZonMW (MKMD project no. 114022504); Health~Holland TKI-LSH PPP-allowance (LSHM17013-H007); and European Union's Horizon 2020 Research and Innovation Programme under the Marie Sklodowska Curie grant agreement no. 707404. We thank the following LUMC colleagues: F.E. van den Hill for performing shRNA-mediated silencing and overexpression experiments of CX43 in hiPSC-cardiac and skin fibroblasts and technical assistance; D. Ward-van Oostwaard for technical assistance; L. Windt for help with immunofluorescence staining and imaging; O. Halaidych for help with calcium experiments; S. Gerhardt for MT cryosectioning and immunostaining; M.J.W.E. Rabelink for help with the production of shCX43 Lentivirus stocks; S.L. Kloet and E. de Meijer (Leiden Genome Technology Center) for help with 10X Genomics experiments (cell encapsulation, library preparation, single-cell sequencing, primary data mapping, and quality control); and LUMC hiPSC core facility for providing primary human dermal fibroblasts. The graphical abstract was created with https://BioRender.com.

AUTHOR CONTRIBUTIONS

Conceptualization, V.V.O., M.B., and C.L.M.; Methodology, E.G., V.M., G.C., V.V.O., M.B., and C.L.M.; Software, M.L.-T., D.G.M., and L.G.J.T.; Formal Analysis, E.G., V.M., G.C., A.C., X.C., R.W.J.v.H., R.C.S., M.M., S.S., B.J.v.M., L.S., H.M., and L.G.J.T.; Investigation, E.G., V.M., G.C., A.C., X.C., R.W.J.v.H., A.K.G., S.K., M.G., D.C.F.S., B.J.v.M., C.R.J., A.A.M., A.J.K., and L.G.J.T.; Resources, E.S., G.P., R.P.D., and A.A.F.d.V.; Writing – Original Draft, E.G., V.V.O., M.B., and C.L.M.; Writing – Review & Editing, E.G., V.M., G.C., V.V.O., M.B., and C.L.M.; Supervision, V.V.O., M.B., and C.L.M.; Project Administration, V.V.O., M.B., and C.L.M.; Funding Acquisition, V.V.O., M.B., and C.L.M.

DECLARATION OF INTERESTS

C.L.M. is co-founder of Ncardia bv.

Received: February 3, 2020
Revised: April 5, 2020
Accepted: May 1, 2020
Published: May 26, 2020

REFERENCES

Agullo-Pascual, E., Reid, D.A., Keegan, S., Sidhu, M., Fenyö, D., Rothenberg, E., and Delmar, M. (2013). Super-resolution fluorescence microscopy of the cardiac connexome reveals plakophilin-2 inside the connexin43 plaque. Cardiovasc. Res. *100*, 231–240.

Anders, S., Pyl, P.T., and Huber, W. (2015). HTSeq–a Python framework to work with high-throughput sequencing data. Bioinformatics *31*, 166–169.

Blazeski, A., Lowenthal, J., Wang, Y., Teuben, R., Zhu, R., Gerecht, S., Tomaselli, G., and Tung, L. (2019). Engineered heart slice model of arrhythmogenic cardiomyopathy using plakophilin-2 mutant myocytes. Tissue Eng. Part A *25*, 725–735.

CellPress
OPEN ACCESS

Bray, M.-A., Vokes, M.S., and Carpenter, A.E. (2015). Using CellProfiler for automatic identification and measurement of biological objects in images. Curr. Protoc. Mol. Biol. *109*, 14.17.1–14.17.13.

Brette, F., and Orchard, C. (2007). Resurgence of cardiac t-tubule research. Physiology (Bethesda) *22*, 167–173.

Carpenter, A.E., Jones, T.R., Lamprecht, M.R., Clarke, C., Kang, I.H., Friman, O., Guertin, D.A., Chang, J.H., Lindquist, R.A., Moffat, J., et al. (2006). CellProfiler: image analysis software for identifying and quantifying cell phenotypes. Genome Biol. *7*, R100.

Carvajal-Vergara, X., Sevilla, A., D'Souza, S.L., Ang, Y.-S., Schaniel, C., Lee, D.-F., Yang, L., Kaplan, A.D., Adler, E.D., Rozov, R., et al. (2010). Patient-specific induced pluripotent stem-cell-derived models of LEOPARD syndrome. Nature *465*, 808–812.

Caspi, O., Huber, I., Gepstein, A., Arbel, G., Maizels, L., Boulos, M., and Gepstein, L. (2013). Modeling of arrhythmogenic right ventricular cardiomyopathy with human induced pluripotent stem cells. Circ. Cardiovasc. Genet. *6*, 557–568.

Cross, M.J., Berridge, B.R., Clements, P.J.M., Cove-Smith, L., Force, T.L., Hoffmann, P., Holbrook, M., Lyon, A.R., Mellor, H.R., Norris, A.A., et al. (2015). Physiological, pharmacological and toxicological considerations of drug-induced structural cardiac injury. Br. J. Pharmacol. *172*, 957–974.

Dell'Era, P., Benzoni, P., Crescini, E., Valle, M., Xia, E., Consiglio, A., and Memo, M. (2015). Cardiac disease modeling using induced pluripotent stem cell-derived human cardiomyocytes. World J. Stem Cells *7*, 329–342.

Denning, C., Borgdorff, V., Crutchley, J., Firth, K.S.A., George, V., Kalra, S., Kondrashov, A., Hoang, M.D., Mosqueira, D., Patel, A., et al. (2016). Cardiomyocytes from human pluripotent stem cells: From laboratory curiosity to industrial biomedical platform. Biochim. Biophys. Acta *1863* (7 Pt B), 1728–1748.

Dobin, A., Davis, C.A., Schlesinger, F., Drenkow, J., Zaleski, C., Jha, S., Batut, P., Chaisson, M., and Gingeras, T.R. (2013). STAR: ultrafast universal RNA-seq aligner. Bioinformatics *29*, 15–21.

Dostal, D., Glaser, S., and Baudino, T.A. (2015). Cardiac fibroblast physiology and pathology. Compr. Physiol. *5*, 887–909.

Dudek, J., Cheng, I.-F., Balleininger, M., Vaz, F.M., Streckfuss-Bömeke, K., Hübscher, D., Vukotic, M., Wanders, R.J.A., Rehling, P., and Guan, K. (2013). Cardiolipin deficiency affects respiratory chain function and organization in an induced pluripotent stem cell model of Barth syndrome. Stem Cell Res. (Amst.) *11*, 806–819.

Eisner, D.A., Caldwell, J.L., Kistamás, K., and Trafford, A.W. (2017). Calcium and excitation-contraction coupling in the heart. Circ. Res. *121*, 181–195.

Faas, F.G.A., Avramut, M.C., van den Berg, B.M., Mommaas, A.M., Koster, A.J., and Ravelli, R.B.G. (2012). Virtual nanoscopy: generation of ultra-large high resolution electron microscopy maps. J. Cell Biol. *198*, 457–469.

Furtado, M.B., Nim, H.T., Gould, J.A., Costa, M.W., Rosenthal, N.A., and Boyd, S.E. (2014). Microarray profiling to analyse adult cardiac fibroblast identity. Genom. Data *2*, 345–350.

Furtado, M.B., Nim, H.T., Boyd, S.E., and Rosenthal, N.A. (2016). View from the heart: cardiac fibroblasts in development, scarring and regeneration. Development *143*, 387–397.

Gaujoux, R., and Seoighe, C. (2010). A flexible R package for nonnegative matrix factorization. BMC Bioinformatics *11*, 367–369.

Giacomelli, E., Bellin, M., Orlova, V.V., and Mummery, C.L. (2017a). Co-differentiation of human pluripotent stem cells-derived cardiomyocytes and endothelial cells from cardiac mesoderm provides a three-dimensional model of cardiac microtissue. Curr. Protoc. Hum. Genet. *95*, 21.9.1–21.9.22.

Giacomelli, E., Bellin, M., Sala, L., van Meer, B.J., Tertoolen, L.G.J., Orlova, V.V., and Mummery, C.L. (2017b). Three-dimensional cardiac microtissues composed of cardiomyocytes and endothelial cells co-differentiated from human pluripotent stem cells. Development *144*, 1008–1017.

Giacomelli, E., Mummery, C.L., and Bellin, M. (2017c). Human heart disease: lessons from human pluripotent stem cell-derived cardiomyocytes. Cell. Mol. Life Sci. *74*, 3711–3739.

Giraldo, A., Barrett, O.P.T., Tindall, M.J., Fuller, S.J., Amirak, E., Bhattacharya, B.S., Sugden, P.H., and Clerk, A. (2012). Feedback regulation by Atf3 in the endothelin-1-responsive transcriptome of cardiomyocytes: Egr1 is a principal Atf3 target. Biochem. J. *444*, 343–355.

Gomes, J., Finlay, M., Ahmed, A.K., Ciaccio, E.J., Asimaki, A., Saffitz, J.E., Quarta, G., Nobles, M., Syrris, P., Chaubey, S., et al. (2012). Electrophysiological abnormalities precede overt structural changes in arrhythmogenic right ventricular cardiomyopathy due to mutations in desmoplakin-A combined murine and human study. Eur. Heart J. *33*, 1942–1953.

Guadix, J.A., Orlova, V.V., Giacomelli, E., Bellin, M., Ribeiro, M.C., Mummery, C.L., Pérez-Pomares, J.M., and Passier, R. (2017). Human pluripotent stem cell differentiation into functional epicardial progenitor cells. Stem Cell Reports *9*, 1754–1764.

Gupta, M.K., Illich, D.J., Gaarz, A., Matzkies, M., Nguemo, F., Pfannkuche, K., Liang, H., Classen, S., Reppel, M., Schultze, J.L., et al. (2010). Global transcriptional profiles of beating clusters derived from human induced pluripotent stem cells and embryonic stem cells are highly similar. BMC Dev. Biol. *10*, 98.

Haghverdi, L., Lun, A.T.L., Morgan, M.D., and Marioni, J.C. (2018). Batch effects in single-cell RNA-sequencing data are corrected by matching mutual nearest neighbors. Nat. Biotechnol. *36*, 421–427.

Hayakawa, T., Kunihiro, T., Dowaki, S., Uno, H., Matsui, E., Uchida, M., Kobayashi, S., Yasuda, A., Shimizu, T., and Okano, T. (2012). Noninvasive evaluation of contractile behavior of cardiomyocyte monolayers based on motion vector analysis. Tissue Eng. Part C Methods *18*, 21–32.

Ibrahim, M., Siedlecka, U., Buyandelger, B., Harada, M., Rao, C., Moshkov, A., Bhargava, A., Schneider, M., Yacoub, M.H., Gorelik, J., et al. (2013). A critical role for Telethonin in regulating t-tubule structure and function in the mammalian heart. Hum. Mol. Genet. *22*, 372–383.

Iwaki, K., Sukhatme, V.P., Shubeita, H.E., and Chien, K.R. (1990). Alpha- and beta-adrenergic stimulation induces distinct patterns of immediate early gene expression in neonatal rat myocardial cells. fos/jun expression is associated with sarcomere assembly; Egr-1 induction is primarily an alpha 1-mediated response. J. Biol. Chem. *265*, 13809–13817.

Joshi, N.A., and Fass, J.N. (2011). Sickle: a sliding-window, adaptive, quality-based trimming tool for FastQ files. https://github.com/najoshi/sickle.

Kehat, I., Kenyagin-Karsenti, D., Snir, M., Segev, H., Amit, M., Gepstein, A., Livne, E., Binah, O., Itskovitz-Eldor, J., and Gepstein, L. (2001). Human embryonic stem cells can differentiate into myocytes with structural and functional properties of cardiomyocytes. J. Clin. Invest. *108*, 407–414.

Klesen, A., Jakob, D., Emig, R., Kohl, P., Ravens, U., and Peyronnet, R. (2018). Cardiac fibroblasts : Active players in (atrial) electrophysiology? Herzschrittmacherther. Elektrophysiol. *29*, 62–69.

Kofron, C.M., Kim, T.Y., King, M.E., Xie, A., Feng, F., Park, E., Qu, Z., Choi, B.R., and Mende, U. (2017). G_q-activated fibroblasts induce cardiomyocyte action potential prolongation and automaticity in a three-dimensional microtissue environment. Am. J. Physiol. Heart Circ. Physiol. *313*, H810–H827.

Kostidis, S., Addie, R.D., Morreau, H., Mayboroda, O.A., and Giera, M. (2017). Quantitative NMR analysis of intra- and extracellular metabolism of mammalian cells: a tutorial. Anal. Chim. Acta *980*, 1–24.

Kuppusamy, K.T., Jones, D.C., Sperber, H., Madan, A., Fischer, K.A., Rodriguez, M.L., Pabon, L., Zhu, W.-Z., Tulloch, N.L., Yang, X., et al. (2015). Let-7 family of microRNA is required for maturation and adult-like metabolism in stem cell-derived cardiomyocytes. Proc. Natl. Acad. Sci. USA *112*, E2785–E2794.

Lazzarini, E., Jongbloed, J.D.H., Pilichou, K., Thiene, G., Basso, C., Bikker, H., Charbon, B., Swertz, M., van Tintelen, J.P., and van der Zwaag, P.A. (2015). The ARVD/C genetic variants database: 2014 update. Hum. Mutat. *36*, 403–410.

Legland, D., Arganda-Carreras, I., and Andrey, P. (2016). MorphoLibJ: integrated library and plugins for mathematical morphology with ImageJ. Bioinformatics *32*, 3532–3534.

Lemoine, M.D., Mannhardt, I., Breckwoldt, K., Prondzynski, M., Flenner, F., Ulmer, B., Hirt, M.N., Neuber, C., Horváth, A., Kloth, B., et al. (2017). Human

iPSC-derived cardiomyocytes cultured in 3D engineered heart tissue show physiological upstroke velocity and sodium current density. Sci. Rep. *7*, 5464.

Liu, J., Volkers, L., Jangsangthong, W., Bart, C.I., Engels, M.C., Zhou, G., Schalij, M.J., Ypey, D.L., Pijnappels, D.A., and de Vries, A.A.F. (2018). Generation and primary characterization of iAM-1, a versatile new line of conditionally immortalized atrial myocytes with preserved cardiomyogenic differentiation capacity. Cardiovasc. Res. *114*, 1848–1859.

Lombardi, R., Dong, J., Rodriguez, G., Bell, A., Leung, T.K., Schwartz, R.J., Willerson, J.T., Brugada, R., and Marian, A.J. (2009). Genetic fate mapping identifies second heart field progenitor cells as a source of adipocytes in arrhythmogenic right ventricular cardiomyopathy. Circ. Res. *104*, 1076–1084.

Lun, A.T.L., Bach, K., and Marioni, J.C. (2016). Pooling across cells to normalize single-cell RNA sequencing data with many zero counts. Genome Biol. *17*, 75.

Mahoney, V.M., Mezzano, V., Mirams, G.R., Maass, K., Li, Z., Cerrone, M., Vasquez, C., Bapat, A., Delmar, M., and Morley, G.E. (2016). Connexin43 contributes to electrotonic conduction across scar tissue in the intact heart. Sci. Rep. *6*, 26744.

Mannhardt, I., Breckwoldt, K., Letuffe-Brenière, D., Schaaf, S., Schulz, H., Neuber, C., Benzin, A., Werner, T., Eder, A., Schulze, T., et al. (2016). Human engineered heart tissue: analysis of contractile force. Stem Cell Rep. *7*, 29–42.

Martin, M. (2011). Cutadapt removes adapter sequences from high-throughput sequencing reads. EMBnet. J. *17*, 10–12.

Mathur, A., Loskill, P., Shao, K., Huebsch, N., Hong, S., Marcus, S.G., Marks, N., Mandegar, M., Conklin, B.R., Lee, L.P., and Healy, K.E. (2015). Human iPSC-based cardiac microphysiological system for drug screening applications. Sci. Rep. *5*, 8883.

Matthes, S.A., Taffet, S., and Delmar, M. (2011). Plakophilin-2 and the migration, differentiation and transformation of cells derived from the epicardium of neonatal rat hearts. Cell Commun. Adhes. *18*, 73–84.

McCarthy, D.J., Chen, Y., and Smyth, G.K. (2012). Differential expression analysis of multifactor RNA-seq experiments with respect to biological variation. Nucleic Acids Res. *40*, 4288–4297.

Moretti, A., Bellin, M., Welling, A., Jung, C.B., Lam, J.T., Bott-Flügel, L., Dorn, T., Goedel, A., Höhnke, C., Hofmann, F., et al. (2010). Patient-specific induced pluripotent stem-cell models for long-QT syndrome. N. Engl. J. Med. *363*, 1397–1409.

Mummery, C., Ward-van Oostwaard, D., Doevendans, P., Spijker, R., van den Brink, S., Hassink, R., van der Heyden, M., Opthof, T., Pera, M., de la Riviere, A.B., et al. (2003). Differentiation of human embryonic stem cells to cardiomyocytes: role of coculture with visceral endoderm-like cells. Circulation *107*, 2733–2740.

Ng, E.S., Davis, R., Stanley, E.G., and Elefanty, A.G. (2008). A protocol describing the use of a recombinant protein-based, animal product-free medium (APEL) for human embryonic stem cell differentiation as spin embryoid bodies. Nat. Protoc. *3*, 768–776.

Ongstad, E., and Kohl, P. (2016). Fibroblast-myocyte coupling in the heart: potential relevance for therapeutic interventions. J. Mol. Cell. Cardiol. *91*, 238–246.

Oxford, E.M., Musa, H., Maass, K., Coombs, W., Taffet, S.M., and Delmar, M. (2007). Connexin43 remodeling caused by inhibition of plakophilin-2 expression in cardiac cells. Circ. Res. *101*, 703–711.

Pellman, J., Zhang, J., and Sheikh, F. (2016). Myocyte-fibroblast communication in cardiac fibrosis and arrhythmias: mechanisms and model systems. J. Mol. Cell. Cardiol. *94*, 22–31.

Pinto, A.R., Ilinykh, A., Ivey, M.J., Kuwabara, J.T., D'Antoni, M.L., Debuque, R., Chandran, A., Wang, L., Arora, K., Rosenthal, N.A., and Tallquist, M.D. (2016). Revisiting cardiac cellular composition. Circ. Res. *118*, 400–409.

Pioner, J.M., Racca, A.W., Klaiman, J.M., Yang, K.-C., Guan, X., Pabon, L., Muskheli, V., Zaunbrecher, R., Macadangdang, J., Jeong, M.Y., et al. (2016). Isolation and mechanical measurements of myofibrils from human induced pluripotent stem cell-derived cardiomyocytes. Stem Cell Reports *6*, 885–896.

Pointon, A., Pilling, J., Dorval, T., Wang, Y., Archer, C., and Pollard, C. (2017). From the cover: high-throughput imaging of cardiac microtissues for the assessment of cardiac contraction during drug discovery. Toxicol. Sci. *155*, 444–457.

Te Riele, A.S., Agullo-Pascual, E., James, C.A., Leo-Macias, A., Cerrone, M., Zhang, M., Lin, X., Lin, B., Sobreira, N.L., Amat-Alarcon, N., et al. (2017). Multilevel analyses of SCN5A mutations in arrhythmogenic right ventricular dysplasia/cardiomyopathy suggest non-canonical mechanisms for disease pathogenesis. Cardiovasc. Res. *113*, 102–111.

Risebro, C.A., Vieira, J.M., Klotz, L., and Riley, P.R. (2015). Characterisation of the human embryonic and foetal epicardium during heart development. Development *142*, 3630–3636.

Robinson, M.D., McCarthy, D.J., and Smyth, G.K. (2010). edgeR: a Bioconductor package for differential expression analysis of digital gene expression data. Bioinformatics *26*, 139–140.

Rog-Zielinska, E.A., Norris, R.A., Kohl, P., and Markwald, R. (2016). The living scar–cardiac fibroblasts and the injured heart. Trends Mol. Med. *22*, 99–114.

Ronaldson-Bouchard, K., Ma, S.P., Yeager, K., Chen, T., Song, L., Sirabella, D., Morikawa, K., Teles, D., Yazawa, M., and Vunjak-Novakovic, G. (2018). Advanced maturation of human cardiac tissue grown from pluripotent stem cells. Nature *556*, 239–243.

Sala, L., Bellin, M., and Mummery, C.L. (2017). Integrating cardiomyocytes from human pluripotent stem cells in safety pharmacology: has the time come? Br. J. Pharmacol. *174*, 3749–3765.

Sala, L., van Meer, B.J., Tertoolen, L.G.J., Bakkers, J., Bellin, M., Davis, R.P., Denning, C., Dieben, M.A.E., Eschenhagen, T., Giacomelli, E., et al. (2018). MUSCLEMOTION: a versatile open software tool to quantify cardiomyocyte and cardiac muscle contraction in vitro and in vivo. Circ. Res. *122*, e5–e16.

Sato, P.Y., Coombs, W., Lin, X., Nekrasova, O., Green, K.J., Isom, L.L., Taffet, S.M., and Delmar, M. (2011). Interactions between ankyrin-G, Plakophilin-2, and Connexin43 at the cardiac intercalated disc. Circ. Res. *109*, 193–201.

Schindelin, J., Arganda-Carreras, I., Frise, E., Kaynig, V., Longair, M., Pietzsch, T., Preibisch, S., Rueden, C., Saalfeld, S., Schmid, B., et al. (2012). Fiji: an open-source platform for biological-image analysis. Nat. Methods *9*, 676–682.

Siu, C.-W., Lee, Y.-K., Ho, J.C.-Y., Lai, W.-H., Chan, Y.-C., Ng, K.-M., Wong, L.-Y., Au, K.-W., Lau, Y.-M., Zhang, J., et al. (2012). Modeling of lamin A/C mutation premature cardiac aging using patient-specific induced pluripotent stem cells. Aging (Albany NY) *4*, 803–822.

Sommariva, E., Brambilla, S., Carbucicchio, C., Gambini, E., Meraviglia, V., Dello Russo, A., Farina, F.M., Casella, M., Catto, V., Pontone, G., et al. (2016). Cardiac mesenchymal stromal cells are a source of adipocytes in arrhythmogenic cardiomyopathy. Eur. Heart J. *37*, 1835–1846.

Sommariva, E., Stadiotti, I., Perrucci, G.L., Tondo, C., and Pompilio, G. (2017). Cell models of arrhythmogenic cardiomyopathy: advances and opportunities. Dis. Model. Mech. *10*, 823–835.

Sreedhar, K., and Panlal, B. (2012). Enhancement of images using morphological transformation. Int. J. Comput. Sci. Info. Technol. *4*, 33–50.

Stewart, W.W. (1978). Functional connections between cells as revealed by dye-coupling with a highly fluorescent naphthalimide tracer. Cell *14*, 741–759.

Takahashi, A., Camacho, P., Lechleiter, J.D., and Herman, B. (1999). Measurement of intracellular calcium. Physiol. Rev. *79*, 1089–1125.

Takahashi, K., Tanabe, K., Ohnuki, M., Narita, M., Ichisaka, T., Tomoda, K., and Yamanaka, S. (2007). Induction of pluripotent stem cells from adult human fibroblasts by defined factors. Cell *131*, 861–872.

Tallquist, M.D., and Molkentin, J.D. (2017). Redefining the identity of cardiac fibroblasts. Nat. Rev. Cardiol. *14*, 484–491.

Thompson, S.A., Copeland, C.R., Reich, D.H., and Tung, L. (2011). Mechanical coupling between myofibroblasts and cardiomyocytes slows electric conduction in fibrotic cell monolayers. Circulation *123*, 2083–2093.

Tiburcy, M., Hudson, J.E., Balfanz, P., Schlick, S., Meyer, T., Chang Liao, M.-L., Levent, E., Raad, F., Zeidler, S., Wingender, E., et al. (2017). Defined engineered human myocardium with advanced maturation for applications in heart failure modeling and repair. Circulation *135*, 1832–1847.

Valle, G., Faulkner, G., De Antoni, A., Pacchioni, B., Pallavicini, A., Pandolfo, D., Tiso, N., Toppo, S., Trevisan, S., and Lanfranchi, G. (1997). Telethonin, a novel sarcomeric protein of heart and skeletal muscle. FEBS Lett. *415*, 163–168.

van den Berg, C.W., Okawa, S., Chuva de Sousa Lopes, S.M., van Iperen, L., Passier, R., Braam, S.R., Tertoolen, L.G., del Sol, A., Davis, R.P., and Mummery, C.L. (2015). Transcriptome of human foetal heart compared with cardiomyocytes from pluripotent stem cells. Development *142*, 3231–3238.

van den Berg, C.W., Elliott, D.A., Braam, S.R., Mummery, C.L., and Davis, R.P. (2016). Differentiation of human pluripotent stem cells to cardiomyocytes under defined conditions. Methods Mol. Biol. *1353*, 163–180.

van den Brink, S.C., Sage, F., Vértesy, Á., Spanjaard, B., Peterson-Maduro, J., Baron, C.S., Robin, C., and van Oudenaarden, A. (2017). Single-cell sequencing reveals dissociation-induced gene expression in tissue subpopulations. Nat. Methods *14*, 935–936.

van Meer, B.J., Krotenberg, A., Sala, L., Davis, R.P., Eschenhagen, T., Denning, C., Tertoolen, L.G.J., and Mummery, C.L. (2019). Simultaneous measurement of excitation- contraction coupling parameters identifies mechanisms underlying contractile responses of hiPSC-derived cardiomyocytes. Nature Communications. https://doi.org/10.1038/s41467-019-12354-8.

Verkerk, A.O., Veerman, C.C., Zegers, J.G., Mengarelli, I., Bezzina, C.R., and Wilders, R. (2017). Patch-clamp recording from human induced pluripotent stem cell-derived cardiomyocytes: improving action potential characteristics through dynamic clamp. Int. J. Mol. Sci. *18*, 1873.

Wagner, F., Yan, Y., and Yanai, I. (2018). K-nearest neighbor smoothing for high-throughput single-cell RNA-seq data. bioRxiv. https://doi.org/10.1101/217737.

Wang, G., McCain, M.L., Yang, L., He, A., Pasqualini, F.S., Agarwal, A., Yuan, H., Jiang, D., Zhang, D., Zangi, L., et al. (2014). Modeling the mitochondrial cardiomyopathy of Barth syndrome with induced pluripotent stem cell and heart-on-chip technologies. Nat. Med. *20*, 616–623.

Wu, T.D., and Nacu, S. (2010). Fast and SNP-tolerant detection of complex variants and splicing in short reads. Bioinformatics *26*, 873–881.

Xu, X.Q., Soo, S.Y., Sun, W., and Zweigerdt, R. (2009). Global expression profile of highly enriched cardiomyocytes derived from human embryonic stem cells. Stem Cells *27*, 2163–2174.

Xu, T., Le, T.D., Liu, L., Su, N., Wang, R., Sun, B., Colaprico, A., Bontempi, G., and Li, J. (2017). CancerSubtypes: an R/Bioconductor package for molecular cancer subtype identification, validation and visualization. Bioinformatics *33*, 3131–3133.

Yang, X., Pabon, L., and Murry, C.E. (2014). Engineering adolescence: maturation of human pluripotent stem cell-derived cardiomyocytes. Circ. Res. *114*, 511–523.

Yu, G., Wang, L.-G., Han, Y., and He, Q.-Y. (2012). clusterProfiler: an R package for comparing biological themes among gene clusters. OMICS *16*, 284–287.

Zhang, S.-S., and Shaw, R.M. (2014). Trafficking highways to the intercalated disc: new insights unlocking the specificity of connexin 43 localization. Cell Commun. Adhes. *21*, 43–54.

Zhang, M., D'Aniello, C., Verkerk, A.O., Wrobel, E., Frank, S., Ward-van Oostwaard, D., Piccini, I., Freund, C., Rao, J., Seebohm, G., et al. (2014). Recessive cardiac phenotypes in induced pluripotent stem cell models of Jervell and Lange-Nielsen syndrome: disease mechanisms and pharmacological rescue. Proc. Natl. Acad. Sci. USA *111*, E5383–E5392.

Zhao, J., Cao, H., Tian, L., Huo, W., Zhai, K., Wang, P., Ji, G., and Ma, Y. (2017). Efficient differentiation of TBX18$^+$/WT1$^+$ epicardial-like cells from human pluripotent stem cells using small molecular compounds. Stem Cells Dev. *26*, 528–540.

STAR★METHODS

KEY RESOURCES TABLE

REAGENT or RESOURCE	SOURCE	IDENTIFIER
Antibodies		
Anti-Troponin I antibody (H-170) (TNNI) for immunofluorescence (1:500)	Santa Cruz Biotechnology	Cat# sc-15368; RRID:AB_793465
Anti-alpha-Actinin (Sarcomeric) antibody (ACTN2) for immunofluorescence (1:800)	Sigma–Aldrich	Cat# A7811; RRID:AB_476766
Anti-CD31/PECAM-1 antibody for immunofluorescence (1:200)	R&D Systems	Cat# AF806; RRID:AB_355617
Anti-Collagen Type I, clone 5D8-G9 antibody (COL1A1) for immunofluorescence (1:200)	Millipore	Cat# MAB3391; RRID:AB_94839
Anti-Wilm's Tumor Protein antibody (WT1) for immunofluorescence (1:200)	Millipore	Cat# CA1026-50UL; RRID:AB_437848
Anti-TBX18 antibody (TBX18(for immunofluorescence (1:200)	Sigma-Aldrich	Cat# HPA029014; RRID:AB_10601597
Anti-ZO-1 antibody (ZO-1) for immunofluorescence (1:200)	Thermo Fisher Scientific	Cat# 61-7300; RRID:AB_2533938
Anti-Ki67 Antibody (Ki67) for immunofluorescence (1:200)	Abcam	Cat# ab833; RRID:AB_306483
Anti-Connexin 43/GJA1 antibody (CX43) for immunofluorescence (1:200)	Abcam	Cat# ab11370; RRID:AB_297976
Anti-alpha-Smooth Muscle Actin, Clone 1A4 antibody (αSMA) for immunofluorescence (1:200)	Sigma-Aldrich	Cat# A2547; RRID:AB_476701
Anti-Plakophilin 2, clone 518 antibody (PKP2) for immunofluorescence (1:25)	Progen	Cat# 651157
Cy3-AffiniPure Donkey Anti-Mouse IgG (H+L) antibody for immunofluorescence (1:100)	Jackson ImmunoResearch Labs	Cat# 715-165-150; RRID:AB_2340813
Donkey anti-Rabbit IgG (H+L) Highly Cross-Adsorbed Secondary Antibody, Alexa Fluor 488 for immunofluorescence (1:200)	Thermo Fisher Scientific	Cat# A-21206; RRID:AB_2535792
Donkey anti-Mouse IgG (H+L) Highly Cross-Adsorbed Secondary Antibody, Alexa Fluor 594 for immunofluorescence (1:200)	Thermo Fisher Scientific	Cat# A-21203; RRID:AB_2535789
Donkey Anti-Sheep IgG (H+L) Antibody, Alexa Fluor 647 for immunofluorescence (1:200)	Thermo Fisher Scientific	Cat# A21448; RRID:AB_10374882
Anti-Plakophilin 2, Clone 28 antibody (PKP2) for western blot (1:1000)	BD Biosciences	Cat# 610788; RRID:AB_398109
Anti-Glyceraldehyde-3-PDH antibody (GAPDH) for western blot (1:2000)	Millipore	Cat# MAB374; RRID:AB_2107445
Anti-mouse IgG, HRP-linked antibody for western blot (1:2000)	Cell Signaling Technology	Cat# 7076; RRID:AB_330924
Bacterial and Virus Strains		
Lentivirus expressing short hairpin RNA (shRNA) targeting Cx43	Sigma Aldrich	TRCN0000059773
Lentivirus expressing Cx43 (LV.hCMV-IE.HsGJA1.IRES.PurR.hHBVPRE)	Liu et al., 2018 PMID: 29917042	N/A
Empty vector (LV.hCMV-IE.IRES.PurR.hHBVPRE)	Liu et al., 2018 PMID: 29917042	N/A
Chemicals, Peptides, and Recombinant Proteins		
Essential 8 Medium	Thermo Fisher Scientific	A1517001
Matrigel hESC-Qualified Matrix	Corning	354277

(Continued on next page)

Continued

REAGENT or RESOURCE	SOURCE	IDENTIFIER
Vitronectin, truncated recombinant human (VTN-N)	Thermo Fisher Scientific	A14700
Fibronectin bovine plasma	Sigma Aldrich	F1141
RevitaCell Supplement (100X)	Thermo Fisher Scientific	A2644501
UltraPure 0.5 M EDTA, pH 8.0	Thermo Fisher Scientific	15575020
TrypLE Select, 10x	Thermo Fisher Scientific	A1217701
Recombinant Human BMP-4 Protein	R&D Systems	314-BP
Human Activin A, premium grade	Miltenyi Biotec	130-115-010
CHIR 99021	Axon Medchem	Axon1386
XAV939	Tocris	3748/10
Human VEGF, premium grade	Miltenyi Biotec	130-109-386
Retinoic Acid	Sigma Aldrich	R2625
TGFβ inhibitor, SB431542	Tocris	1614/10
Human FGF-2, premium grade	Miltenyi Biotec	130-093-842
Fibroblast Growth Medium 3	PromoCell	C-23025
CryoStor CS10 medium	Stem Cell Technologies	07930
Puromycin dihydrochloride	Sigma Aldrich	P7255
Polybrene	Sigma Aldrich	H9268
Collagenase type II	Worthington	LS004176
Fluo-4, AM, cell permeant	Thermo Fisher Scientific	F14201
Tissue-Tek® OCT	Sakura® Finetek	4583
Verapamil hydrochloride	Tocris	0654
Bay-K8644	Tocris	1544
Caffeine	Sigma Aldrich	C0750
Dibutyryl cyclic-AMP (dbcAMP)	Sigma Aldrich	D0627
Oligomycin	Sigma Aldrich	O4876
Carbonyl cyanide 4-(trifluoromethoxy) phenylhydrazone (FCCP)	Sigma Aldrich	C2920
Antimycin A	Sigma Aldrich	A8674
Rotenone	Sigma Aldrich	R8875
2-Deoxy-D-glucose (2-DG)	Sigma Aldrich	D8375
Critical Commercial Assays		
EasySep Human CD34 Positive Selection Kit II	Stem Cell Technologies	17856
Multi Tissue Dissociation Kit 3	Miltenyi Biotec	130-110-204
Quant-iT PicoGreen dsDNA Assay Kit	Thermo Fisher Scientific	P7589
Direct cAMP ELISA kit	Enzo Life Sciences	ADI-900-066
NucleoSpin RNA Kit	Macherey- Nagel	740955
iScript-cDNA Synthesis kit	Bio-Rad	170-8889
iTaq Universal SYBR® Green Supermix	Bio-Rad	1725124
BCA Protein Assay Kit	Thermo Fisher Scientific	23227
SupersignalWest Dura Extended Duration Substrate	Thermo Fisher Scientific	37071
Deposited Data		
Gene expression (bulk RNA-sequencing)	This paper	GEO: GSE116464
Gene expression (single-cell RNA-sequencing)	This paper	GEO: GSE147694
Experimental Models: Cell Lines		
CTRL1 hiPSC line	LUMC hiPSC core facility	LUMC0020iCTRL-06 https://hpscreg.eu/cell-line/LUMCi028-A Zhang et al., 2014 PMID: 25453094

(*Continued on next page*)

Continued

REAGENT or RESOURCE	SOURCE	IDENTIFIER
CTRL2 hiPSC line	LUMC hiPSC core facility	LUMC0099iCTRL04 https://hpscreg.eu/cell-line/LUMCi004-A
CTRL3 hiPSC line	LUMC hiPSC core facility	LUMC0059iCTRL03
CTRL hiPSC line NCRM1	NIH Center for Regenerative Medicine NIH CRM, obtained from RUDCR Infinite Biologics at Rutgers University	(Guadix et al., 2017) PMID: 29173898
LQT1 hiPSC line	Zhang et al., 2014 PMID: 25453094	LUMC0021iKCNQ-30 Zhang et al., 2014 PMID: 25453094
ACM hiPSC line	This study	LUMC0153iPKP
CTRL1 SFs	LUMC hiPSC core facility	WK12220
CTRL2 SFs	LUMC hiPSC core facility	WK12022
ACM SFs	Monzino Hospital, Milan	WK13262
Human adult cardiac fibroblasts	PromoCell	C-12375
Software and Algorithms		
Fiji-ImageJ	Schindelin et al., 2012 PMID: 22743772	https://imagej.net/Fiji/Downloads
CellProfiler	Carpenter et al., 2006 PMID: 17076895	https://cellprofiler.org
IMARIS	Oxfornd Instruments	N/A
MATLAB	Mathworks Inc	N/A
Aperio ImageScope	Leica	N/A
GraphPad Prism 8.2.0	GraphPad	N/A
MUSCLEMOTION	Sala et al., 2018 PMID: 29282212	https://www.ahajournals.org/doi/suppl/10.1161/CIRCRESAHA.117.312067
RStudio	RStudio	https://rstudio.com/products/rstudio
Origin 2016	OriginLab	N/A
Clampex 10.0	Molecular Devices Axon Instruments	N/A
LabVIEW	National Instruments	N/A
LUMC BIOPET Gentrap	LUMC Sequencing Analysis Support Core	https://github.com/biopet/biopet
Python	Python Software Foundation	https://www.python.org
Image Lab	Bio-Rad	N/A

RESOURCE AVAILABILITY

Lead Contact

Further information and requests for resources and reagents should be directed to and will be fulfilled by the Lead Contact, Prof. Christine L. Mummery (C.L.Mummery@lumc.nl).

Materials Availability

hiPSC lines are available upon MTA.

Data and Code Availability

The accession numbers for the bulk and single cell RNA sequencing datasets reported in this paper are https://www.ncbi.nlm.nih.gov/geo/ GEO: GSE116464 (bulk) and GEO: GSE147694 (single cell). Software used to analyze the data are either freely or commercially available. Motion Flow analysis software can be requested by contacting L.G.J.Tertoolen@lumc.nl.

EXPERIMENTAL MODEL AND SUBJECT DETAILS

Ethics statement

Protocols for research involving human subjects and stem cell research were approved by the medical ethical committee at Leiden University Medical Center, the Netherlands and Centro Cardiologico Monzino, Milan, Italy. Written informed consent was received from participants prior to inclusion in the study.

hiPSC lines culture

CTRL1 hiPSC line LUMC0020iCTRL-06 (female, Giacomelli et al., 2017a, 2017b; Zhang et al., 2014), CTRL2 hiPSC line LUMC0099iCTRL04 (female), CTRL3 hiPSC line LUMC0059iCTRL03 (female), LQT1 hiPSC line LUMC0021iKCNQ-30 (female, Zhang et al., 2014) and ACM hiPSC line LUMC0153iPKP (female, see below for mutation) were generated from primary skin fibroblasts using Sendai virus by the LUMC hiPSC core facility. CTRL hiPSC line NCRM1 (NIH Center for Regenerative Medicine NIH CRM) was obtained from RUDCR Infinite Biologics at Rutgers University. All the hiPSC lines used in this study were assessed for pluripotency and routinely tested for mycoplasma and genomic integrity by karyotyping. All hiPSC lines were seeded on vitronectin recombinant human protein and cultured in E8 medium as described previously (Giacomelli et al., 2017a, 2017b). Cells were passaged twice a week using PBS containing EDTA 0.5 mM. RevitaCell Supplement (1:200) was added during passaging (all from Thermo Fisher Scientific).

Clinical history and genetic phenotype of ACM patient

A 37 years old woman presented with inverted T waves V1-V2 on the ECG but was without dysfunctional and structural alterations at echocardiography and magnetic resonance imaging (MRI). Family history indicated sudden cardiac death in the family (mother and maternal uncle died suddenly in their thirties) and a brother was clinically affected by ACM. Sequencing of the *PKP2* gene (GenBank: NM_004572.3) identified the heterozygous c.2013delC mutation in exon 10, which causes a frameshift (p.Lys672fs) and premature termination in exon 10 (Figure 7A) in the brother and the patient. Two years later, the patient developed frequent premature ventricular contraction and was hospitalized for further medical assessment and possibly loop recorder implant. ECG showed repolarization (inverted T waves V1-V2), depolarization, and conduction (ε wave) abnormalities and MRI documented mild right ventricle enlargement. Endomyocardial biopsy at this stage showed a limited area of fibro-fatty substitution and inflammatory cells, and another area mostly composed of adipose tissue (not shown).

Primary fibroblasts culture

Human adult cardiac fibroblasts (ACFs; PromoCell) were cultured in FGM3 (PromoCell) medium following the manufacturer's instructions; human adult skin fibroblasts (CTRL1 SFs WK12220, CTRL2 SFs WK12022 from Leiden University Medical Center hiPSC core facility, ACM SFs WK13262, from Monzino Hospital, Milan) were cultured in DMEM/F12 Glutamax medium supplemented with 10% FBS, 1% Non-essential amino acids (NEAA), 1% penicillin/streptomycin (pen/strep) and 0.18% 2-mercaptoethanol (all from Thermo Fisher Scientific). Cell dissociation was carried out using TrypLE 1X for 5 min at 37°C, 5% CO_2, followed by centrifugation for 3 min at 1100 rpm and resuspension either in FGM3 medium (ACFs) or DMEM/F12 supplemented medium (SFs). Fibroblasts (10cm^2 per vial) were cryopreserved in CryoStor CS10 medium (0.5ml/vial; Stem Cell Technologies).

Human fetal heart ECs and fibroblasts

RNA from human fetal heart ECs and fibroblasts was isolated at passage 1 (P1) after collection from fetal heart samples at gestation ages Week(W)12.5, W15 and W21. Human fetal tissue samples were obtained from elective abortion material (vacuum aspiration) without medical indication through approval to Dr Chuva de Sousa Lopes by the Medical Ethical Committee of Leiden Medical University Center (P08.087). Informed consent was obtained and the study was conducted in accordance with the Declaration of Helsinki by the World Medical Association.

METHOD DETAILS

Differentiation of hiPSCs into CMs, cardiac ECs and EPI

CM differentiation was induced in monolayer as described previously (Giacomelli et al., 2017a, 2017b; van den Berg et al., 2016). Briefly, 25 × 10^3 cells per cm^2 (CTRL1, CTRL2, CTRL3) were seeded on plates coated with 75 μg/ml growth factor-reduced Matrigel (Corning) the day before differentiation (day −1). On day 0, cardiac mesoderm was induced by changing E8 to BPEL medium (Bovine Serum Albumin [BSA] Polyvinyl alcohol Essential Lipids (Ng et al., 2008), supplemented with a mixture of cytokines (20 ng/ml BMP4, R&D Systems; 20 ng/ml ACTIVIN A, Miltenyi Biotec; 1.5 μM GSK3 inhibitor CHIR99021, Axon Medchem). After 3 days, cytokines were removed and the Wnt inhibitor XAV939 (5 μM, Tocris) was added for 3 days. BPEL medium was refreshed every 3 days. Cardiac EC differentiation was induced in monolayer as described previously (Giacomelli et al., 2017a, 2017b). Briefly, 15 × 10^3 cells per cm^2 (CTRL1, CTRL2) were seeded on Matrigel at day −1. On day 0, cardiac mesoderm was induced as described above. On day 3, cytokines were removed and VEGF (50 ng/ml, R&D Systems) added with XAV939 (5 μM). Cardiac ECs were isolated as described previously (Giacomelli et al., 2017a, 2017b) using a Human cord blood CD34 Positive selection kit II (StemCell Technologies) following the manufacturer's instructions. For cardiac ECs culture, 15 × 10^3 cells per cm^2 cells were seeded on fibronectin-coated plates and cultured in BPEL medium supplemented with VEGF (50ng/ml). After 3-4 days, cells were confluent and cells (30cm^2 per vial) were cryopreserved in CryoStor® CS10 medium (0.5ml per vial; StemCell Technologies). EPI differentiation was induced in monolayer as described previously (Guadix et al., 2017). Briefly, 25 × 10^3 cells per cm^2 (CTRL1, CTRL2, CTRL4) and 30 × 10^3 per cm^2 (LQT1, ACM) were seeded on Matrigel at day −1. On day 0, cardiac mesoderm was induced as described above. After 3 days, cytokines were removed and the WNT inhibitor XAV939 (5 μM) added for 3 days with BMP4 (30 ng/ml) and Retinoic Acid (RA; 1 μM; Sigma Aldrich). On day 6, BPEL medium supplemented with BMP4 (30 ng/ml) and RA (1 μM) was refreshed. On day 9, 15 × 10^3 per cm^2 (CTRL1, CTRL2, CTRL4) and 20 × 10^3 per cm^2 (LQT1, ACM) were seeded on plates coated with 2-5 μg/ml of fibronectin from bovine plasma (fibronectin; Sigma Aldrich) in BPEL medium supplemented with the TGFβ inhibitor SB431542 (10 μM; Tocris).

By day 12, EPI were confluent and ready for passage or analysis. EPI cells (30 cm^2 per vial) were cryopreserved in CryoStor CS10 medium (0.5 ml/vial; Stem Cell Technologies).

Differentiation of hiPSC-EPI into CFs

CF differentiation was induced in monolayer, similarly to Zhao et al. (2017). Briefly, 25 × 10^3 EPI (CTRL1, CTRL2, CTRL4 LQT1, ACM) were seeded per cm^2 on tissue culture plates coated with vitronectin in BPEL medium supplemented with FGF2 (10 ng/ml; R&D Systems) on day 12. On day 13 and every 2 days thereafter, medium was refreshed with BPEL supplemented with FGF2 (10 ng/ml). After 6 days (on day 19), CFs were expanded by changing BPEL to Fibroblast Growth Medium 3 (FGM3; PromoCell). FGM3 was refreshed every 2 days for approximately 10 days in total. After 10 days (on day 29), CFs were confluent and ready to be passaged at 1:2 ratio. FGM3 was refreshed the day after passaging and every 2 days thereafter. CFs (10cm^2 per vial) were cryopreserved in CryoStor CS10 medium (0.5ml/vial; Stem Cell Technologies).

Lentiviral transduction of hiPSC-CFs using shRNA-CX43

Lentivirus expressing short hairpin RNA (shRNA) targeting Cx43 (Sigma, TRCN0000059773) was used to downregulate Cx43 in CTRL1 hiPSC-CFs (Cx43-sh-RNA CFs). Briefly, one day after seeding 60000 cells/12-well, hiPSC-CFs were transduced with viral particles at MOI 1 in fresh FGM3 with 8 μg/mL polybrene overnight. GFP expressing virus (pLV-CMV-GFP) was used as a control (scramble shRNA). 72 h post-transduction, when control cells expressed GFP, infected cells were selected with 1 μg/mL puromycin (Sigma, P7255). After 3 days, remaining cells were fixed for immunofluorescence staining, collected for RNA extraction and dissociated to prepare MTs.

Lentiviral transduction for CX43 overexpression in primary SFs

Lentivirus expressing Cx43 (LV.hCMV-IE.HsGJA1.IRES.PurR.hHBVPRE) (Liu et al., 2018) was used to overexpress Cx43 in CTRL2 SFs WK12022. Briefly, one day after seeding 40000 cells/12-well, SFs were transduced with 2,5 μl viral particles (5*10^8 TU) in fresh medium with 8 μg/mL polybrene overnight. Empty lentiviral vector (LV.hCMV-IE.IRES.PurR.hHBVPRE) was used as a control. 72 h post-transduction, infected cells were selected with 1 μg/mL puromycin (Sigma, P7255). After 3 days, remaining cells were expanded (1:3 ratio), then dissociated and replated for immunofluorescence staining and used to prepare MTs.

3D cardiac microtissue (MT) culture

Prior to MT formation, hiPSC-ECs (CTRL1, CTRL2) and hiPSC-CFs (CTRL1, CTRL2, ACM) were prepared as follows: 2-3 days before MT formation, a vial of cryopreserved hiPSC-ECs and a vial of cryopreserved hiPSC-CFs were thawed and cultured either in BPEL medium supplemented with VEGF on plates coated with fibronectin (hiPSC-ECs), or in FGM3 on uncoated plates (hiPSC-CFs). On the day of MT formation (day 0), hiPSC-ECs, hiPSC-CFs, SFs, ACFs, Cx43-sh-RNA CFs, SFs Empty LV and SFs CX43 LV were detached using TrypLE 1X for 5 min at 37°C, 5% CO_2, centrifuged for 3 min at 1100 rpm, resuspended in BPEL medium and counted. hiPSC-CMs at day 14-21 (CTRL1, CTRL2) that showed > 80% purity, measured as the percentage of troponin positive cells by FACS, were dissociated using the Multi Tissue Dissociation Kit 3 (Miltenyi Biotec) following the manufacturer's instructions, resuspended in BPEL medium and counted. For CMECs: cell suspensions were combined to a total of 5000 cells (85% cardiomyocytes and 15% endothelial cells) per 50 μL BPEL medium supplemented with VEGF (50 ng/ml). For CMFs: cell suspensions were combined to a total of 5000 cells (85% cardiomyocytes and 15% cardiac fibroblasts) per 50 μL BPEL medium supplemented with FGF2 (5 ng/ml). For CMECFs: cell suspensions were combined to a total of 5000 cells (70% cardiomyocytes, 15% endothelial cells and 15% cardiac fibroblasts) per 50 μL BPEL medium supplemented with VEGF (50 ng/ml) and FGF2 (5 ng/ml). MTs composed of primary fibroblasts (ACFs, CTRL1 SFs, CTRL2 SFs, ACM SFs) were prepared as described for CMECFs. For all MTs, cell suspensions were seeded on V-bottomed 96-well microplates (Greiner bio-one) and centrifuged for 10 min at 1100 rpm. MTs were incubated at 37°C, 5% CO_2 for 21 days with media refreshed every 3-4 days. Dibutyryl cAMP (dbcAMP) treatment was performed on CMECFs and CMEC SFs from CTRL1 and CTRL2 in BPEL medium supplemented with 0.5 mM dbcAMP for 7 days (from day 14 to day 21). Analysis of all MTs was performed after 21 days, unless otherwise indicated in Figure legends. For experiments that required cell dissociation, MTs were incubated in 290 U/mg of pre-warmed collagenase type II (Worthington) in HBSS solution (Sigma Aldrich). Single cells were used for either scRNaseq or seeded on 24-well Matrigel-coated plates on top of glass coverslips (10 mm diameter) for immunofluorescence and electrophysiology.

OCT cryosections of 3D microtissues

Microtissues were fixed in 4% PFA at RT for 20 min and were embedded in Tissue-Tek® OCT compound (Sakura® Finetek) for further analysis. Thick frozen serial cross sections (8 μm) were processed for immunofluorescence staining.

Immunofluorescence analysis

For immunofluorescence staining, primary fibroblasts were dissociated using TrypLE 1X for 5 min at 37°C, 5% CO_2 and 20 × 10^3 cells/well were seeded on a 96-well/plate (plastic, 96-well Black/Clear tissue culture treated plate; Falcon). hiPSC-derived cells were dissociated as above and seeded either on Matrigel- (CMs: 40 × 10^3) or fibronectin- (EPI: 20 × 10^3) or vitronectin- (CFs: 20 × 10^3) coated plates, on a 96-well/plate (plastic, 96-well Black/Clear tissue culture treated plate). MTs were dissociated as above and 50 and 100 × 10^3 single cells were seeded on 24-well Matrigel-coated plates, on top of glass coverslips (10 mm diameter).

CellPress
OPEN ACCESS

Medium was refreshed the following day. Cells were fixed either after 7-10 days (CMs and MTs, to allow recovery) or after 2-3 days (all other cell types) for 20 min in 4% paraformaldehyde, permeabilized for 10 min with PBS (Calcium, Magnesium, Thermo Fisher Scientific) containing 0.1% Triton X-100 (Sigma Aldrich) and blocked for at least 1 h with PBS (Calcium, Magnesium) containing 10% FCS. Primary antibodies were added overnight at 4°C on a shaker. The following day, cells were washed 3 times with PBS (Calcium, Magnesium) at room temperature, each time incubated for 10/15 min. Secondary antibodies were added for 1h at 37°C and protected from light. Cells were washed three times with PBS (Calcium, Magnesium), each time incubated for 10 min and stained with DAPI (Thermo Fisher Scientific) for 10 min at room temperature. Images were captured with an EVOS FL AUTO2 microscope, using a 10x and 20x magnification objective. For dissociated MTs, images were captured with a Leica SP8WLL confocal laser-scanning microscope, using a 63x magnification objective and Z stack acquisition. Whole mount immunofluorescence staining of 3D cardiac MTs was performed as described previously (Giacomelli et al., 2017a, 2017b). Briefly, MTs were washed in PBS (Calcium, Magnesium) on day 21 and fixed for 1h at 4°C with 4% paraformaldehyde, washed 3 times in PBS (Calcium, Magnesium) and stored at 4°C until processing. MTs were permeabilized for 30 min with PBS (Calcium, Magnesium) containing 0.2% Triton X-100 and blocked for 2h in PBS (Calcium, Magnesium) containing 10% FCS. All incubations were at room temperature. Primary antibodies were added overnight at 4°C. The following day, MTs were washed 3 times with PBS (Calcium, Magnesium) at room temperature, each time incubated for 10 min. Secondary antibodies were added overnight at 4°C. The following day, MTs were washed 3 times with PBS (Calcium, Magnesium) at room temperature, each time incubated for 20-30 min and then stained with DAPI (1:500) for 1h at room temperature. MTs were mounted with ProLong Gold antifade Mountant (Thermo Fisher Scientific) onto microscope slides on top of 12 mm round, glass coverslips. Images were captured with a Leica SP8 WLL confocal laser-scanning microscope, using a 25x, 40x, or 100x objectives and Z stack acquisition or using Andor Dragonfly spinning disk confocal microscope with a 100x objective.

Transmission Electron Microscopy (TEM)

MTs were fixed for 1hr in 1.5% glutaraldehyde in 0.1 M cacodylate buffer (pH 7.4) by adding an equal volume of double concentrated (3%) fixative to the culture medium. After rinsing the MTs three times in 0.1M cacodylate buffer, they were postfixed in 1% OsO_4/0,1M cacodylate solution for 1h on ice. Next, MTs were dehydrated in 70% EtOH overnight. The next day, MTs were dehydrated in 80 and 90% EtOH for 10 mins, and twice in 100% EtOH for 30 min. MTs were then embedded in a mixture of propylene oxide and EPON (2:1, 1:1 and 1:2, for 30 min each followed by pure EPON for 60 min. MTs were transferred in an embedding capsule and EPON was added. EPON was allowed to harden for two days at 70°C. Samples were sliced using a elementsix ultramicrotome knife (Drukker) in a Reichert Ultracut S microtome (Leica) into 80 nm ultrathin slices and mounted on copper grids before being stained with saturated uranyl acetate (20 μl) in the dark at room temperature for 10 min. MTs were then washed ten times in milliQ and twice in NaOH (0.01 M). Next, MTs were stained for 5 min with lead citrate (20 μl) and washed 10 times in 0.01% NaOH/MilliQ solution and ten times in milliQ before being air-dried and imaged with a Twin electron microscope (Tecnai T12Twin, Fei, Eindhoven). Samples were imaged with the Gatan camera (One View) by stitching several photographs together (Faas et al., 2012). Stitches and sarcomere length were analyzed using Aperio ImageScope (version 12.3.2.8013, Leica). Quantitative analysis of sarcomere length was performed on TEM images by calculating the distance between consecutive Z-lines (distance from the middle of one Z-line to the middle of the next) using Aperio ImageScope. Statistical analysis was performed using GraphPad Prism 8.2.0.

Contraction analysis

MTs were seeded on 24-well Matrigel-coated plates on top of plastic 13 mm coverslips (Sarstedt). Movies of spontaneous or paced MTs were acquired for at least 10 s at 37°C either with a ThorLabs DCC3240M camera at 100 frames/sec and a 10 x objective phase contrast objective (Leica Inverted microscope IBDE), using the ThorLabs uc480 software (v 4.20), either with a Leica Microsystems LAS AF6000 microscope at 37°C and 5% CO_2. For verapamil and Bay-K8644 experiments, videos of MTs were acquired under perfusion after 5 min of incubation in baseline condition (Tyrode's) or drug. During the last min of incubation, MTs were stimulated at 1.5 Hz, with 12V/cm strength and 3 ms long stimulation pulse. For ACM and CX43 overexpression data, movies were recorded from MTs paced at 1, 2 or 3 Hz and kept in their V-bottomed culture plate, at 37°C and 5%CO_2. Contraction data were obtained by analyzing movies either with the MUSCLEMOTION ImageJ macro (ImageJ v. 2.0.0-rc-49) as described previously (Sala et al., 2018), or with Vector Flow analysis. Contraction duration of spontaneously beating MTs was normalized to their spontaneous beating rate: Normalized contraction duration = contraction duration / sqrt (IBI).

For ACM data, MT pacing experiments were analyzed using an R script calculating the contraction rate and the coefficient of variation (CV) of contraction amplitudes. MTs were classified as correctly following the applied stimulation frequency when the rate was equals to the pacing rate ± 15% and when the contraction amplitude CV was lower than a chosen cutoff (28%, which accounts for random amplitude variations).

The Horn-Schunck Vector Flow analysis method was used to detect changes in pixel displacements during contraction of the micro tissues in general according to ref. (Hayakawa et al., 2012). The analysis package was developed with LabVIEW Motion and Vision (National instruments U.S.A). Images were collected at 100 frames/sec with a Thor Labs camera DCC3260M (Thorlabs GmbH 85221 Munich, Germany) and a 10X objective phase contrast objective (Leica Inverted microscope IBDE). Cells were perfused with a Tyrode solution at 37°C and paced at 1 Hz. Tyrode's contained (in mM): 140 NaCl, 5.4 KCl, 1.8 CaCl2, 1.0 MgCl2, 5.5 glucose, 5.0 HEPES; pH 7.4 (NaOH). The μm/pixel was calibrated and used to compute the horizontal and vertical velocity and the resultant velocity. Binning of the vectors was carried out over 15 × 15 μm^2 at an average 300x300 μm selection. Of each image, the maximum velocity bin of all bins was selected to compute the resulting contraction/relaxation profiles. The maximum velocity was selected

either on an automated or manually chosen position within the selected area of interest. Standard deviations were computed for all bins and plotted at the contraction/relaxation trajectories in order to quantify the deviations from the peak values. The direction of the contraction was calculated computing the angular direction of the vertical and horizontal components (arc tangent). Experiments were analyzed over at least 5 repetitive contractions per experimental condition. From the contraction/relaxation profile, five parameters were analyzed. The velocity of the upstroke (μm/sec) the acceleration of the upstroke (μm/sec^2), the velocity of relaxation (μm/sec), the acceleration of relaxation (μm/sec^2) and the beat duration. For all individual data points presented, data from at least 3 separate MTs were pooled (SD) or individual measurements were averaged to obtain the number of observations (SEM). From the computed vectors, line integrals were computed with the Advanced Plotting Toolkit (National Instruments).

Sharp Electrode Electrophysiology

MTs were seeded on 24-well Matrigel- coated plates, on top of plastic 13 mm coverslips (Sarstedt). CMECFs were manually cut as necessary into smaller pieces before measurement. Sharp electrodes were fabricated with a Sutter Flaming Brown puller 97 (Sutter Instruments, CA, USA). Electrodes were filled with 3M KCl having a final resistance of 80 – 100 MOhm (estimated tip diameter of 0.2 μm). A Dagan intracellular IX1 amplifier with a Buzz unit (Dagan Minneapolis MN USA) was used. MTs were perfused with Tyrode's solution at 37°C, gently touched by the electrodes and penetrated using the zap function of the amplifier Buzz unit using an MP-200 micromanipulator (Sutter Instruments, CA, USA). APs were stored with an Axon Digidata 1322A and pClamp 10 software at 10 kHz (Axon Molecular Devices USA).

Patch clamp electrophysiology

For dibutyryl cAMP (dbcAMP) experiments in 2D culture, CTRL1 hiPSC-CMs were dissociated with TrypLE 1X for 5 min at 37°C, 5% CO_2, centrifuged for 3 min at 1100, plated on glass coverslips (10 mm diameter) coated with Matrigel at a density of 40.000 cells/well and cultured for 72 hr either in Pluricyte medium (NCardia) (CTRL) or in medium containing 0.5 mM dbcAMP before analysis. Patch clamp measurements were performed on small groups of cells (10-20) with Axopatch 200B patch clamp amplifier at 5 kHz in the fast current-clamp mode. Recordings only with seal resistances of 2.5 GOhm or higher were selected for further analysis. Data were digitized with a DigiData 1322A at 10 kHz under control of Clampex 10.0 software (Molecular Devices Axon Instruments U.S.A.). Pipets used had a resistance of ~2.4 MOhm. Pipet buffer composition: K-gluconate 125 mM; KCl 20 mM; NaCl 5 mM; HEPES 10 mM adjusted to pH 7.3 with KOH. Amphotericin-B (Sigma-Aldrich U.S.A) was added to the pipet buffer before start of the measurements at a concentration of 3 μg/ml from a DMSO stock of 0.6 mg/ml (light protected). Cells were continuously perfused in a perfusion chamber at 37°C (Cell MicroControls Norfolk VA, U.S.A.) with Tyrode's solution. For MT experiments: single cells were dissociated from all groups of MTs in parallel on day 21. Cells were re-plated on top of glass coverslips (10 mm diameter) at low density to obtain isolated single cells. After 1 week, coverslips were placed onto the stage of an inverted microscope for patch clamp recordings. Cells were kept at 36 ± 1°C and perfused with Tyrode's physiological solution containing (mmol/L): 140 NaCl, 5.4 KCl, 1.8 $CaCl_2$, 1 $MgCl_2$, 5.5 D-glucose, 5 HEPES-NaOH, pH 7.4. Cardiomyocytes were selected based on morphology and spontaneous contraction. APs were recorded with Axopatch 200B amplifier, digidata 1440A and pClamp10.7 software (Molecular Devices) using patch clamp in whole cell perforated patch configuration and current clamp mode. The pipette solution contained (mmol/L): 125 K-gluconate, 20 KCl, 10 NaCl, 10 HEPES, 0.5 amphotericin B (Sigma), pH 7.2. APs were recorded by stimulating for 2 ms at 1 Hz both without and with dynamic clamp technique for computed I_{K1} injection, as previously shown (Verkerk et al., 2017). AP duration, amplitude and membrane resting potential were analyzed from recordings without I_{K1} dynamic injection, whereas V_{max} was analyzed on dynamic clamp recordings to reduce variability due to different resting membrane potentials. The percentage of APs with a "notch" was obtained using R script by calculating the minimum derivative value of AP traces in the peak region. Notches were identified for derivative values < -3, a cut-off set to account for trace random oscillations. Analysis was performed using RStudio, OriginPro (Origin Lab) 2016 and GraphPad Prism 8.2.0.

Calcium analysis

MTs were seeded on a Matrigel-coated 96-well/plates (plastic, 96-well Black/Clear tissue culture treated plate), and loaded with 5 μM Fluo-4 AM (Thermo Fisher Scientific) for 30 min at 37°C. Medium was changed (BPEL) and left for 30 min at 37°C before recordings. For recordings, MTs were stimulated at 1.5 Hz on a Leica Microsystems LAS AF6000 microscope and recorded for at least 10 s. All calcium experiments were performed at 37°C and 5% CO_2. Analysis was performed using a custom-made ImageJ macro to implement the calculation of dF/F as described by Takahashi et al. (1999). Briefly, the background of the recording was selected outside the MT. The maximum projection of the recording was determined to automatically select the area of the MT by thresholding the result at the overall mean intensity plus standard deviation. Finally, a custom-made Labview program was used to detect the kinetics and calcium transients. For caffeine experiments, MTs were plated and loaded as described above. MTs were stimulated at their averaged spontaneous beating frequency. After 4 calcium transients, stimulation was stopped and 50 mM caffeine added immediately and directly to the culture medium. Analysis was performed as described above. To calculate the caffeine induced amplitude increase, the amplitude of the first peak after caffeine addition was divided by the average of the four peaks before caffeine.

Drug preparation

Verapamil hydrochloride (Tocris), Bay-K8644 (Tocris), caffeine (Sigma-Aldrich) and dbcAMP (Sigma-Aldrich) were dissolved in DMSO following the manufacturer's instructions. Stock solutions were freshly prepared before experiments.

Measurement of cAMP in microtissues

Direct cAMP Enzyme-linked Immunosorbent Assay (ELISA) was performed using a direct cAMP ELISA kit (Enzo Life Sciences) following the manufacturer's instructions. Prior to assay procedure, 240 MTs/condition were pooled and lysed in 0.1M HCL and stored at −20°C before experiment.

Oxidative respiration and glycolytic acidification

Oxidative respiration and glycolytic acidification were measured with Seahorse XF-96 Analyzer (Agilent). MTs were collected into a 15 mL Falcon tube and washed three times in assay medium + 0.2% BSA. 4h before measurement, four MTs (or five for the CMEC MT group) per well were plated on 96-well Matrigel-coated (0.167 mg/ml) plates. Measurements were made in minimal DMEM (Sigma) supplemented with 15 mM glucose (Sigma-Aldrich), 2 mM pyruvate (Thermo Fisher Scientific) and 1 mM L-glutamine (Thermo Fisher Scientific) (assay medium). For Mitochondrial stress test, the following drugs were diluted in assay medium: 3.5 μM Oligomycin, 4 μM FCCP, 2 μM Antimycin A and 2 μM Rotenone (all from Sigma-Aldrich). For the glycolytic stress test, MTs were plated in medium without glucose and the injections contained 15 mM glucose, 3.5 μM Oligomycin, and 100 mM 2-DG (Sigma-Aldrich). Normalization was performed by lysing the MTs with a chloride-based lysis buffer (10 mM Tris, 1 mM EDTA, 50 mM KCl, 2 mM $MgCl_2$) supplemented with 200 μg/ml Prot K, for 2 h at 60°C. DNA content was measured using a Picogreen assay (Thermo Fisher Scientific). Analysis was performed using R and R-studio.

Nuclear Magnetic Resonance spectroscopy (NMR)

Two aliquots of culture medium were collected from each sample, first immediately after refreshing the culture medium 24h before harvesting and then right before harvesting. All aliquots were immediately mixed with 2 volumes of cold methanol (−70°C) and stored at −80°C. For analysis of intracellular metabolites, 60 MTs/condition were pooled together and spun down for 5 s, culture medium was aspirated and pellets were washed with ice-cold PBS. After 5 s of mild centrifugation, PBS was aspirated and MTs were immersed in liquid nitrogen to completely quench metabolic activity. Intracellular metabolites were extracted by addition of 0.6 mL methanol/chloroform/water, 6.75:0.75:2.5 (v/v/v) and repeating cycles of 10 s of vortexing, 1 min sonication and 1 min resting on ice, for a total of 15 min. Subsequently, all samples (culture medium and MT extracts) were centrifuged for 20 min at 18000 × *g* at −4°C and collected supernatants were dried under a gentle flow of nitrogen. NMR samples were prepared by dissolving the dried material with 0.22 mL of 0.15 M phosphate buffer (pH 7.4) in deuterated water containing 0.05 mM trimethylsilyl propionic-d_4-sodium salt (TSP-d_4) as internal standard for NMR referencing and quantification. An 1D ^{1}H-NMR spectrum was collected for each sample on a 14.1 T (600 MHz for ^{1}H) Bruker Avance II NMR, using the 1D-NOESY experiment with pre-saturation as implemented in the spectrometer library (pulse sequence: *noesygppr1d*). All spectra were processed to correct the phase and baseline and imported in Chenomx NMR suite 8.4 for quantification of metabolites. All concentrations were normalized to the total protein mass of each sample. Details of the experimental procedure and NMR analysis can be found in Kostidis et al. (2017).

Gene expression (qPCR)

For qPCR, total RNA was purified using the NucleoSpin RNA Kit (Macherey- Nagel) according to the manufacturer's protocol. 1 μg of RNA was reverse transcribed by using the iScript-cDNA Synthesis kit (Bio-Rad). Expression profiles of genes of interest were determined by qPCR using either 8ng/μl of cDNA (MTs experiments) or 6 ng/μL of cDNA (all other experiments), and the iTaq Universal SYBR Green Supermixes (Bio-Rad). Gene expression was assessed by a Bio-Rad CFX384 real time system. Gene expression levels were normalized to *RPL37A* and *HARP* housekeeping genes. Results were analyzed by using the ΔCt method. For qPCR on MTs, 60 MTs/condition were pooled together. Primer sequences are provided in Table S6.

Gene expression (bulk RNA-sequencing)

Whole-genome transcriptome data were generated at BGI (Shenzhen, China) using the Illumina Hiseq4000 (100bp reads). Raw data were processed using the LUMC BIOPET Gentrap pipeline (https://github.com/biopet/biopet), which consists of FASTQ preprocessing, alignment and read quantification. Sickle (v1.2) was used to trim low-quality read ends (Joshi and Fass, 2011). Cutadapt (v1.1) was used for adapters clipping (Martin, 2011), reads were aligned to the human reference genome GRCh38 using GSNAP (gmap-2014-12-23) (Dobin et al., 2013; Wu and Nacu, 2010) and gene read quantification with htseq-count (v0.6.1p1) against the Ensembl v87 annotation (Anders et al., 2015). Gene length and GC content bias were normalized using the R package *cqn* (v1.24.0) (Wu and Nacu, 2010). Median chromosome X and Y expression were used to verify the sex of included samples. Genes were excluded if the number of reads was below 5 in ≥ 90% of the samples. The final dataset comprised gene expression levels of 36 samples and 22,227 genes. Differentially expressed genes were identified using generalized linear models as implemented in edgeR (McCarthy et al., 2012). P values were adjusted using the Benjamini-Hochberg procedure and $P_{FDR} \leq 0.05$ was considered significant. Analyses were performed using R (version 3.4.4). Figures were produced with the R package ggplot2 (v2.2.1). Consensus clustering of selected DEGs was performed with CancerSubtypes R package (Xu et al., 2017). Clustering was iterated 500 times for K clusters in the range 2 to 10. Heatmap of genes in all 8 clusters was generated using a heatmap function of NMF R package (Gaujoux and Seoighe, 2010). GO and KEGG pathway enrichment for each cluster of genes was performed using compareCluster function of clusterProfiler R package (v3.10.1) (Yu et al., 2012) and $q \leq 0.05$ was considered significant.

Gene expression (single-cell RNA-sequencing)

Pre-processing, clustering and tSNE

For clustering, replicates of CMs and CMECFs were pre-processed separately. In both cases, undetected genes (genes with a count of one in less than two cells) and the 50 cells with the lowest number of transcripts were removed from further analysis. This resulted in 6810 cells and 12823 genes for the CMs dataset, and 9497 cells and 13958 genes for the CMECFs dataset. Then, k-nearest neighbors (knn) smoothing was performed with the python package *kNN-smoothing* (Version 2.1, (Wagner et al., 2018)) using k = 15, d = 10 and dither = 0.05. Each replicate was normalized individually with the *scran* package in R (V 1.10.1) using the method described in Lun et al. (2016). The 10% most highly variable genes (HVG) for each replicate were calculated with *scran* after excluding ribosomal genes (obtained from the HGNC website without any filtering for minimum gene expression), stressed genes (van den Brink et al., 2017) and mitochondrial genes. Batch effect correction between replicates was performed with a mutual nearest neighbors (MNN)- based approach (Haghverdi et al., 2018) implemented in the *scran* package. (Here we used log transformed, knn-smoothed and normalized count data of the 10% most HVG that were present in all replicates). The MNN algorithm was run with d = 50. Hierarchical clustering was performed on MNN corrected counts using Pearson correlation as distance measure and Ward linkage. For the CM dataset, the hierarchical clustering tree was cut at height 40 which resulted in 2 clusters. For CMECFs, the hierarchical clustering tree was cut at height 12 which resulted in 4 clusters. Among the clusters thus defined, cardiomyocytes were identified by known marker genes from the literature. Then, the cardiomyocytes from each of the four datasets were combined into one dataset comprising 8405 cells and 11472 genes in total. The raw data from these cells was pre-processed again, as described above using the same parameters, where normalization and calculation of HVG was now performed simultaneously for all cardiomyocytes. No batch effect correction was performed. T-distributed stochastic neighbor embedding (tSNE) was performed using the R package *Rtsne*. We used Freeman-Tuckey transformed and MNN corrected values to calculate the Pearson correlation as a distance measure for the tSNE.

Differential expression analysis

The R package *edgeR* (V 3.24.1, (Robinson et al., 2010)) was used to perform differential expression analysis. We used counts and a negative binomial distribution to fit the generalized linear model. The covariates were comprised of four binary dummy variables that indicate the four cardiomyocyte populations and a variable that corresponds to the total number of counts per cell. Finally, p values for a contrast between CMECFs and CMs (CMECFs – CMs) were obtained and adjusted for multiple hypothesis testing with the *Benjamini-Hochberg* method.

Comparison to bulk RNA-sequencing data

Bulk samples were obtained from dataset GEO: GSE62913 (Kuppusamy et al., 2015). For principal component analysis (PCA) the intersection of the 10% most HVG of the single cell cardiomyocyte dataset and genes expressed in the bulk samples were used. Each of the three datasets (merged cardiomyocytes from the scRNA-seq dataset, bulk samples of CMs, CMECs and CMECFs, bulk samples from Kuppusamy et al., 2015) were Freeman-Tuckey transformed and scaled individually before calculating the principal components.

Western blot

hiPSC-CFs and EPI generated from CTRL1, CTRL2 and ACM were lysed on ice in protein extraction buffer (10 mM Tris-HCl pH 7.4, 150 mM NaCl, 1% Igepal CA630, 1% sodium deoxycholate, 0.1% SDS (Sodium Dodecyl Sulfate) and 1% Glycerol supplemented with protease inhibitor mix. Total lysates were quantified using BCA protein kit (Thermo Fisher Scientific) following manufacturer's instructions. Total proteins (30 μg) were resolved by SDS-PAGE gel (4%–15% Criterion Precast Gel, Bio-Rad) and transferred to PVDF membrane (Bio-Rad). The membrane was blocked by incubation with TBS 0.1% Tween, 5% non-fat dry milk for 1 h at room temperature (RT). The membrane was then incubated overnight at 4°C with primary commercial antibodies against PKP2 and GAPDH. The membrane was washed with TBS 0.1% Tween and incubated 1 h at RT with the appropriate HRP-conjugated secondary antibody (Cell Signaling). Detection was performed using the enhanced chemiluminescence system (SupersignalWest Dura Extended Duration Substrate, Thermo Fisher Scientific) and images were acquired with the ChemiDoc MP Imaging System (Bio-rad). Acquired images were quantified using Image Lab software 5.2.1 (Bio-Rad).

Bright field images

Bright field images were acquired with a Nikon DS- 2MBW camera connected to a Nikon Eclipse Ti-S microscope, controlled by the Nikon NIS-Element BR software. Lens magnification was 4x with a PhL contrast filter.

QUANTIFICATION AND STATISTICAL ANALYSIS

Quantitative sarcomere analysis by immunostaining

Sarcomere length

3D cardiac MTs and dissociated MTs were stained for Z-bands (ACTN2) and sarcomere length was analyzed on images captured by confocal microscope using standard analysis plugin in open-source Fiji Software, ImageJ.

Sarcomere organization

Z-band density and bidimensional organization indicating sarcomere alignment were evaluated on 3D cardiac MTs stained for ACTN2 and acquired by confocal microscope. The analysis of sarcomere alignment was performed using a plugin based on Fast Fourier Transform (FFT) algorithm (Fiji Software, ImageJ) as previously described (Pioner et al., 2016). Briefly, the peak components

of the power spectrum curve reproduced the periodicity of Z-bands. The index of sarcomere alignment was obtained by normalizing the area under the first order peak for the total area of the power spectrum profile.

Quantitative CX43 analysis by immunostaining

Quantification of immunostaining was preformed using the open-source software Cellprofiler (Bray et al., 2015; Carpenter et al., 2006). Objects were identified in the pipeline using otsu thresholding to segment DAPI positive nuclei or CX43 positive spots in the respective channels to provide total number of CX43 per cell. For quantification of CX43 and PKP2 intensity, the positive staining was first outlined using Sobel edge-finding method, then total intensity of the image enclosed by the defined edges was quantified and normalized to the number of nuclei. For quantification of intensity at cell junctions only, an additional step to mask the area inside the cell was applied, so that the only defined areas at the cell junctions were quantified.

To quantify SMA positive cells, non-specific small bright objects were first identified using Otsu's thresholding method and cell size and used to mask non-specific signal in the image. Next, using Otsu's thresholding and cell size, parameters were used to segment SMA positive and DAPI positive cells to obtain the percentage of SMA positive cells per image.

3D quantification of CX43 in MTs was preformed using the spot detection function of the IMARIS software.

Computational framework for quantitative analysis

We developed a computational framework in-house for semi-automated quantification of 3D stack reconstructions from confocal sections, which combines computer graphic algorithms, image processing, segmentation, ellipsoidal fitting and 3D object reconstruction based on area superposition. The framework was developed combining Java scripts in Fiji (Schindelin et al., 2012) and MATLAB (Mathworks Inc.). The sequence of instructions implemented in the framework is the following:

Segmentation of nuclei channel:

1. Definition of Kernel Radius (KR): User defines the radius of the kernel required to perform several of the image processing algorithms used in the framework. This parameter is defined attending the minimal radius of interest objects in the image. To analyze images of MTs, a KR of 5 pixels was used.
2. Lighting Homogenization: To reduce differences in intensity along x and y axis of each confocal section, a gray-scale morphological subtraction using an opening image with large kernel (Sreedhar and Panlal, 2012) (5*KR) was performed.
3. Local Background Subtraction: To remove the background intensity, a local threshold defined by the local median intensity (8x KR x KR) was used.
4. Enhancing Object definition: A sequence of filters was applied to remove noise and increase the definition of the boundaries of each object (Median filter, Gaussian Blur, Maximum Filter).
5. Seed Generation: A binary copy of each image was generated using the median as threshold for each slice. Euclidean Distance Map (EDT) was performed in the binary image to generate the primordium points (seeds) for each object.
6. Identification of objects: Segmentation was completed from seeds using a flood fill algorithm. Objects smaller than KR*KR were not considered. Objects n times larger than a threshold defined by the user were divided into n independent objects.

3D reconstruction of nuclei:

After identification of each 2D object in each section of the sample, our framework identified each segmented object in each section of the sample with a 3D object in the stack. An example of MT reconstructed digitally is shown in Figure S1D. This script is used to generate the data for Figure S1B (right panel). This was performed as follows:

1. Ellipsoidal fitting: Each segmented object was fitted to an ellipse, with area and orientation depending on the dimensions and characteristics of the object.
1. Overlapping area: Interactions between segmented objects of adjacent planes were identified based on the amount of overlapping between their fitted ellipses. As a first approximation, an interaction between 2D objects was established if their fitted ellipses overlapped.
2. Initial objects and elongation: If a 2D ellipse did not have an interaction with an object in the previous section, it was identified as the start of a new 3D object. Starting from each initial section, the next section of the object was identified based on the maximal overlap of the ellipses. This reconstruction was performed iteratively until the final section of each 3D object was identified.
3. Final objects: The end of a 3D object was defined by an average size of the object in the z-direction, estimated by the size of the 3D projections of each image (this length in the Z axis was referred as KZ).
4. Parameter analysis: The desired values of each 3D object in the 3D stack were extracted and listed for quantitative analysis (volume, location, orientation, distance to neighbors).

Identification of each cell type:

The information of the marker intensity based on immunostaining was used to determine the identity of each nucleus. This script is used to generate the data for Figure S1C. For the classification of cellular composition of MT in different conditions analyzed, the intensity of the following markers was used: TNN1, COL1A1, CD31.

By assuming that all cells in the tissue had a defined phenotype, and that each cell corresponded to only one of the phenotypes defined, the following tasks were defined:

1. Noise removal: Noise in the form of small pixel-to-pixel variations was removed using a Gaussian blur filter in the sections. Filters were applied with a user defined KR.
2. Intensity normalization: Intensity of all channels containing the identity markers was normalized.
3. Threshold definition: A threshold value for each identity marker was defined in correlation with the others to define the most probable identity of this object. These thresholds were defined by the user based on the characteristics of the staining and the nature of each identity marker.
4. Nuclei classification: Identification of each 3D object was defined as the most frequently occurring identity of all its ellipsoidal 2D sections. If the most frequent staining was TNN1 the cell was identified as cardiomyocyte; COL1A1 corresponded to a fibroblast identity; CD31 to an endothelial phenotype.

Identification of proliferative cells:

For the identification of proliferative and fibroblast cells at day 7 and 21, identities were established based on presence or absence of a staining. This script is used to generate the data for Figure S1E. In case cells could not be assumed as positive for an identity marker, the approach used was:

1. Noise removal: Noise in the form of small pixel-to-pixel variations was removed using a Gaussian blur filter in the sections. Filters were applied with a user defined KR.
2. Morphological operation: Presence of fibers were removed by applying an opening filter using as structuring element a line in 135 degrees respect the horizontal (Legland et al., 2016). Filters were applied with a user defined KR.
3. Lightning Homogenization: To reduce differences in intensity along x and y axis of each confocal section, a gray-scale morphological subtraction using an opening image with large kernel (Sreedhar and Panlal, 2012) (5*KR) was performed
4. Background subtraction: Subtraction of values lower than the result of the sum of the average and standard deviation were performed in each slice.
5. Nuclei classification: Classification of nuclei based on the average intensity value in fitted 2D ellipses was performed for all channels. A cell with an average intensity of KI67 higher than zero was assumed as proliferative. A cell with an average intensity of COL1A1 higher than zero was considered a fibroblast.

Measurement of average distance:

This script was used to generate the data for Figure S1F, by computing the centroid of each 3D object in the stack as follows:

1. Distance between nuclei in the sample and its 12 closest neighbors was computed. The median value was calculated for each nuclei as the most probable distance.
2. Average distance (as well as standard deviation of the average distance) between all cells in the sample was calculated as the average value of all the medians for the whole MT.

Measurement of volume:

This script was used to quantify the average volume of TNN1+ nuclei plotted in Figure S1G as follows:

1. Each 3D object in the stack was fitted to an ellipsoid. The KZ (length in the z axis) in the different samples analyzed was varied based on the average size of 3D objects in the Z-direction: KZ = 18 for images corresponding to day 7 and 21 without fibroblasts; 10 for images corresponding to day 7 and 21 with fibroblasts; 46 for images corresponding to CMECs, whereas 36 for the rest of the images.
2. $TNN1^{+}$ cells were identified using the approach explained above.

The volume of each cell was calculated from the semi-axis distance of each MT.

Statistics

Detailed statistics and statistical significance are indicated in each figure legend. Results with p values $p < 0.05$ were considered statistically significant. Briefly, One-way ANOVA, two-way ANOVA, Student's t test, for paired or unpaired measurements were applied as appropriate to test for differences in means between groups/conditions. Data are expressed and plotted as the Mean ± SEM or Mean ± SD as indicated in figure legends. The sample size used in each experiment is indicated in the figure legends. Statistical analysis was performed using GraphPad Prism 8.2.0 and RStudio.

Cell Stem Cell, Volume *26*

Supplemental Information

Human-iPSC-Derived Cardiac Stromal Cells Enhance Maturation in 3D Cardiac Microtissues and Reveal Non-cardiomyocyte Contributions to Heart Disease

Elisa Giacomelli, Viviana Meraviglia, Giulia Campostrini, Amy Cochrane, Xu Cao, Ruben W.J. van Helden, Ana Krotenberg Garcia, Maria Mircea, Sarantos Kostidis, Richard P. Davis, Berend J. van Meer, Carolina R. Jost, Abraham J. Koster, Hailiang Mei, David G. Míguez, Aat A. Mulder, Mario Ledesma-Terrón, Giulio Pompilio, Luca Sala, Daniela C.F. Salvatori, Roderick C. Slieker, Elena Sommariva, Antoine A.F. de Vries, Martin Giera, Stefan Semrau, Leon G.J. Tertoolen, Valeria V. Orlova, Milena Bellin, and Christine L. Mummery

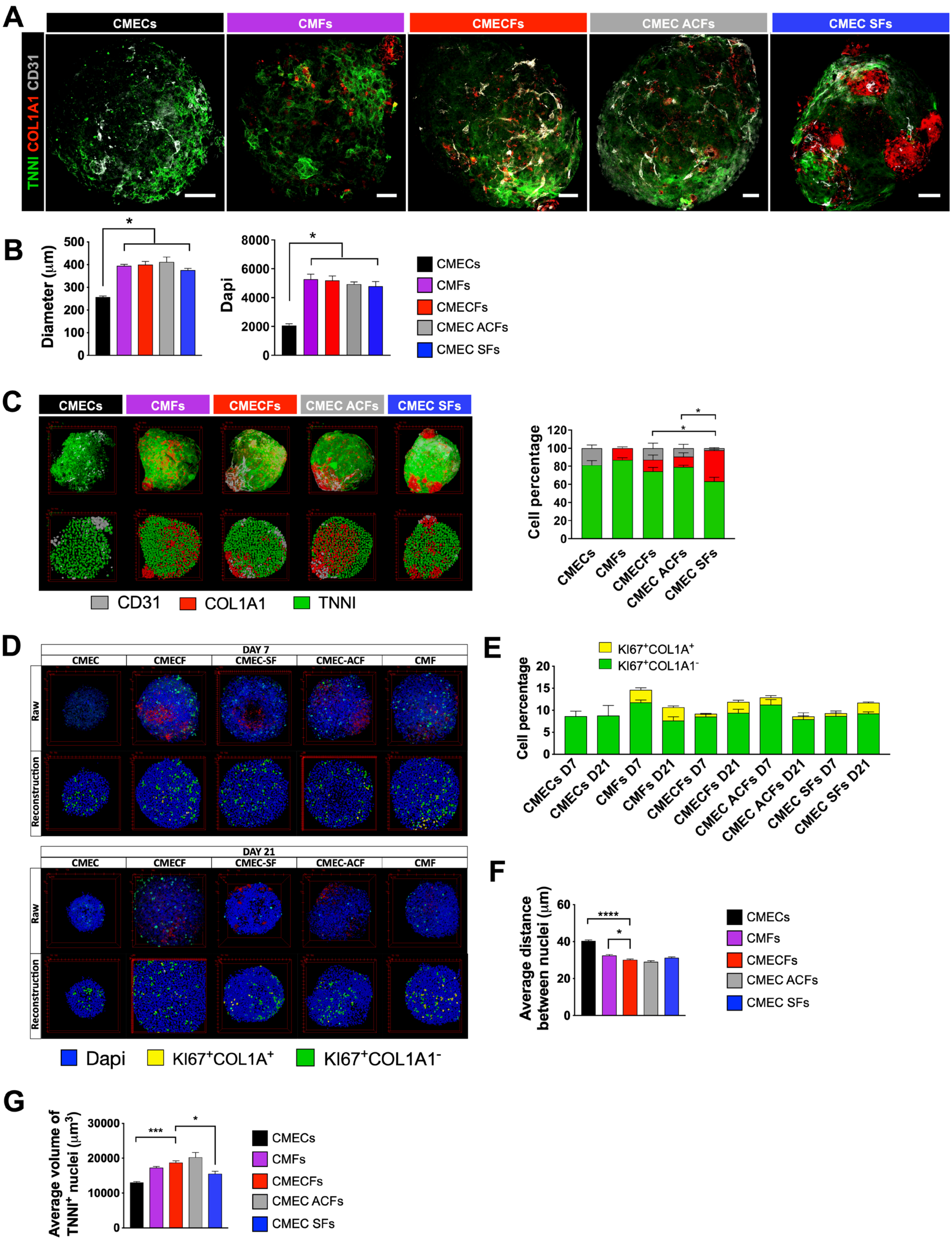
A
CMECs
CMFs
CMECFs
CMEC ACFs
CMEC SFs
TNNI COL1A1 CD31
B
Diameter (µm)
Dapi
CMECs
CMFs
CMECFs
CMEC ACFs
CMEC SFs
C
CD31
COL1A1
TNNI
Cell percentage
D
DAY 7
DAY 21
CMEC
CMECF
CMEC-SF
CMEC-ACF
CMF
Raw
Reconstruction
Dapi
KI67+COL1A+
KI67+COL1A1-
E
Cell percentage
CMECs D7
CMECs D21
CMFs D7
CMFs D21
CMECFs D7
CMECFs D21
CMEC ACFs D7
CMEC ACFs D21
CMEC SFs D7
CMEC SFs D21
F
Average distance between nuclei (µm)
G
Average volume of TNNI+ nuclei (µm3)

Figure S1 (related to Figure 2). Characterization of 3D microtissues. (A) Representative immunofluorescence images of CM- (TNNI, green), EC- (CD31, grey) and fibroblast- (COL1A1, red) markers in MTs, as indicated. Scale bar: 50 µm. (B) Bar graphs showing MT diameter measured in MTs mounted on glass coverslips (left, N>9) and total number of $DAPI^+$ cells measured by 3D semi-automated image processing (right, N=4). *P<0.0001. One-way ANOVA with Dunnett's multiple comparisons test. (C) Representative immunofluorescence images (top) and digital images (bottom) showing $TNN1^+$ (green), $COL1A1^+$ (red), and $CD31^+$ (grey) cells in MTs (left). Bar graph quantification of the same cell populations (right). N=4, *P<0.001 for COL1A1+ cells. Two-way ANOVA with Tukey's multiple comparisons test. (D) Representative immunofluorescence (top, raw) and digital (bottom, reconstruction) images for KI67+$COL1A1^+$ (yellow) and $KI67^+COL1A1^-$ (green) cells. $DAPI^+$ cells in blue. MTs at day 7 (upper panel) and day 21 (lower panel) were analysed. (E) Bar graph showing the percentage of $KI67^+COL1A1^-$ and (green) and $KI67^+COL1A^+$ (yellow) cells in MTs at the indicated time points (D=day). N>3. (F, G) Bar graph showing average distance between nuclei (F) and average volume of $TNNI^+$ nuclei (G) in MTs. N=4 MTs, ****P<0.0001, ***P<0.0005, *P<0.05. One-way ANOVA with Dunnett's multiple comparisons test. All data are shown as mean ± SEM.

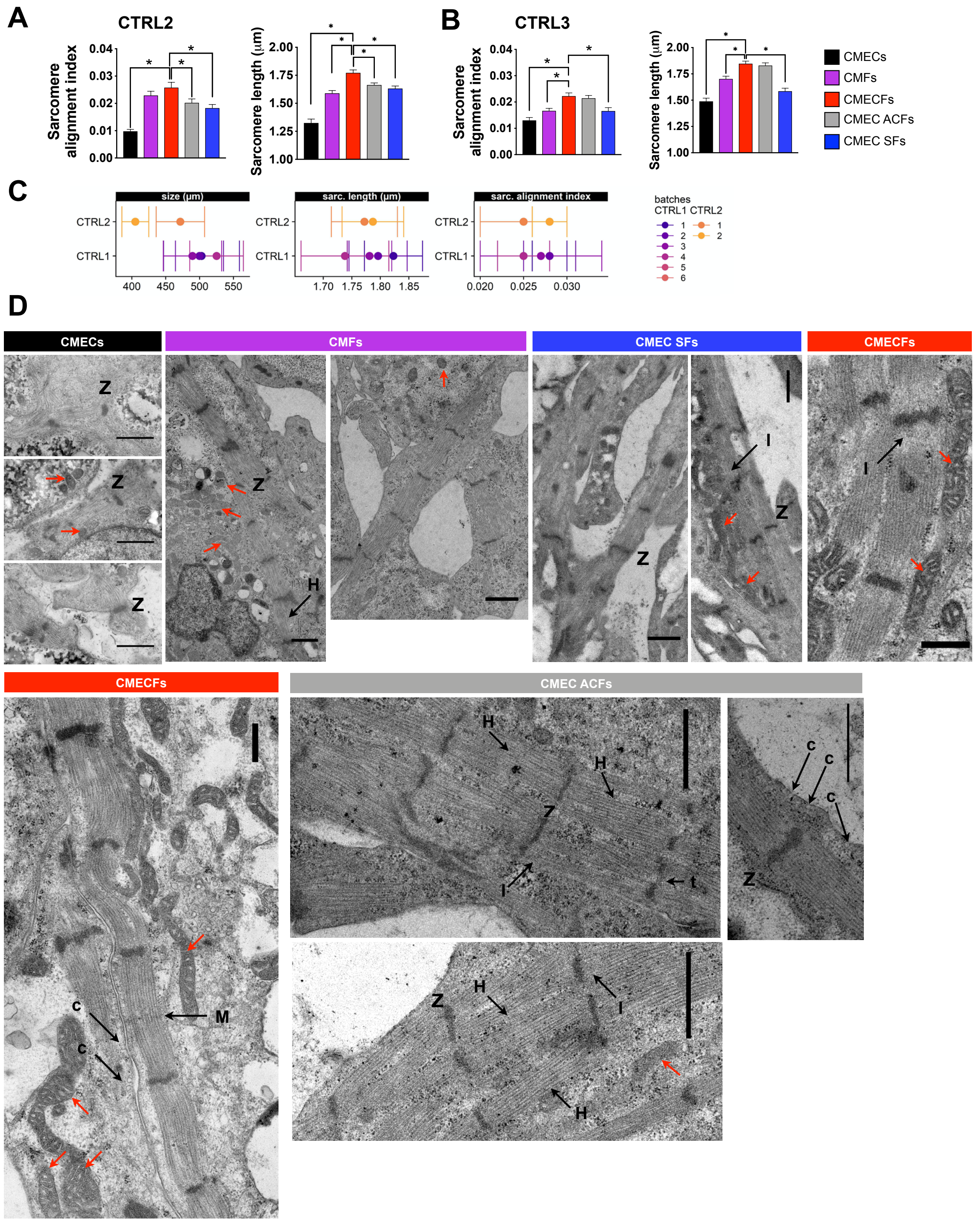

Figure S2 (related to Figure 2). Structural maturation and ultrastructure of 3D microtissues. (A, B) Quantification of sarcomere organization (sarcomere alignment index, left) and sarcomere length (right) in CTRL2 hiPSCs (N>30, *P<0.05) (A) and CTRL3 hiPSCs (N=30, *P<0.005) (B) MTs from immunofluorescence analysis. Data are shown as mean ± SEM. N indicates different areas from 3 independent MTs per group. One-way ANOVA with Dunnett's multiple comparisons test. (C) Graphs showing variability of morphological parameters, i.e. size, sarcomere length, and sarcomere alignment index. Mean values (dots) and SD (error bars) for different MT batches from CTRL1 and CTRL2 hiPSCs are shown. (D) Representative TEM images showing presence or absence of ultrastructural features typical of mature cardiomyocytes in CMECs, CMFs, CMEC SFs, CMECFs and CMEC ACFs: caveolae (c), Z-lines (Z), M-lines (M), I-bands (I), H-zones (H) and mitochondria (red arrows). CMECs, CMFs, CMEC SFs and CMEC ACFs, scale bar: 1 µm. CMECFs, scale bar: 0.5 µm.

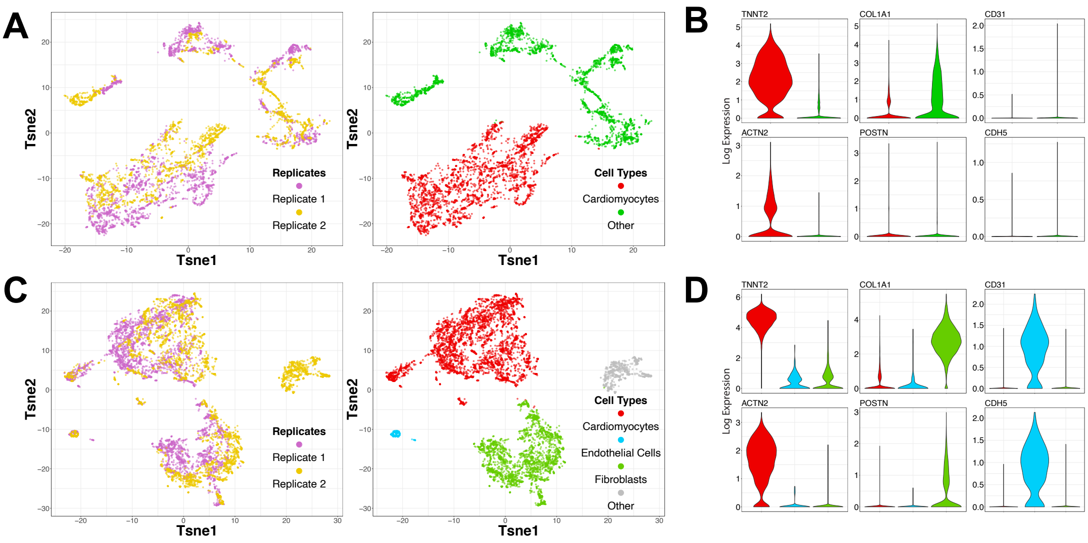

E

Comparison model		Number of upregulated DEGs	
Group1	Group2	Group 1	Group 2
hiPSC-CMs	CMECs	3891	6038
hiPSC-CMs	CMECFs	4488	4600
CMECs	CMECFs	4192	2537
CMECFs	CMEC-SFs	2349	1313
CMECFs	CMEC-ACFs	92	116

F

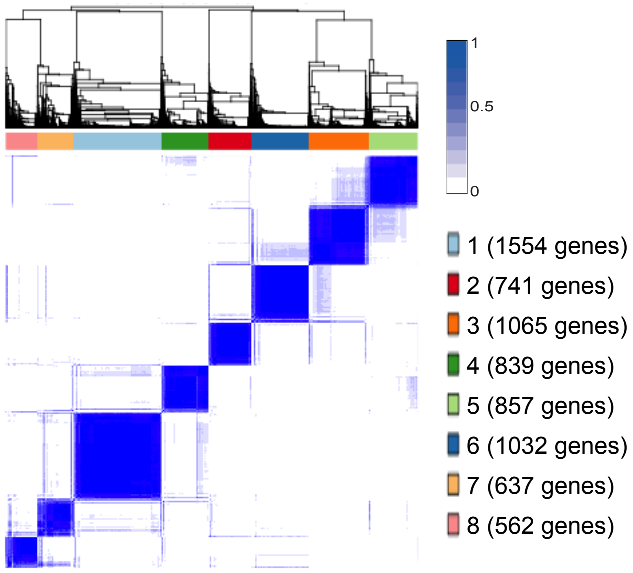

Figure S3 (related to Figure 3). Single-cell and bulk transcriptome profiling of microtissues. (A) Tsne plots of hiPSC-CMs based on scRNA-seq profiles. In the left panel, colors represent different replicate samples (*Replicate 1; Replicate 2*); in the right panel, colors represent different cell type clusters (*Cardiomyocytes; Other*). (B) Violin plots showing expression (log transformed) of selected CM- (*TNNT2*, *ACTN2*), fibroblast- (*COL1A1, POSTN*), and EC- (*CD31, CDH5)* markers in hiPSC-CMs based on their scRNA-seq profiles. (C) Tsne plots of CMECFs based on their scRNA-seq profiles. In the left panel, colors represent different replicate samples (*Replicate 1; Replicate 2*); in the right panel, colors represent different cell type clusters (*Cardiomyocytes; Endothelial cells; Fibroblasts; Other*). (D) Violin plots showing expression (log transformed) of selected CM- (*TNNT2*, *ACTN2*), fibroblast- (*COL1A1, POSTN*), and EC- (*CD31, CDH5)* markers in CMECFs based on their single-cell RNA-seq profile. (E) Total number of DEGs identified across hiPSC-CMs, CMECs, CMECFs, CMEC SFs and CMEC ACFs in bulk RNA-seq analysis. (F) Consensus matrix: DEGs from (E) grouped into eight gene clusters with ConsensusCluster, each row and column represent a unique gene and the color scale represents consensus values, which indicate how often two items occupied the same cluster out of 500 iterations. The color key indicates cluster definitions and the number of genes in each cluster.

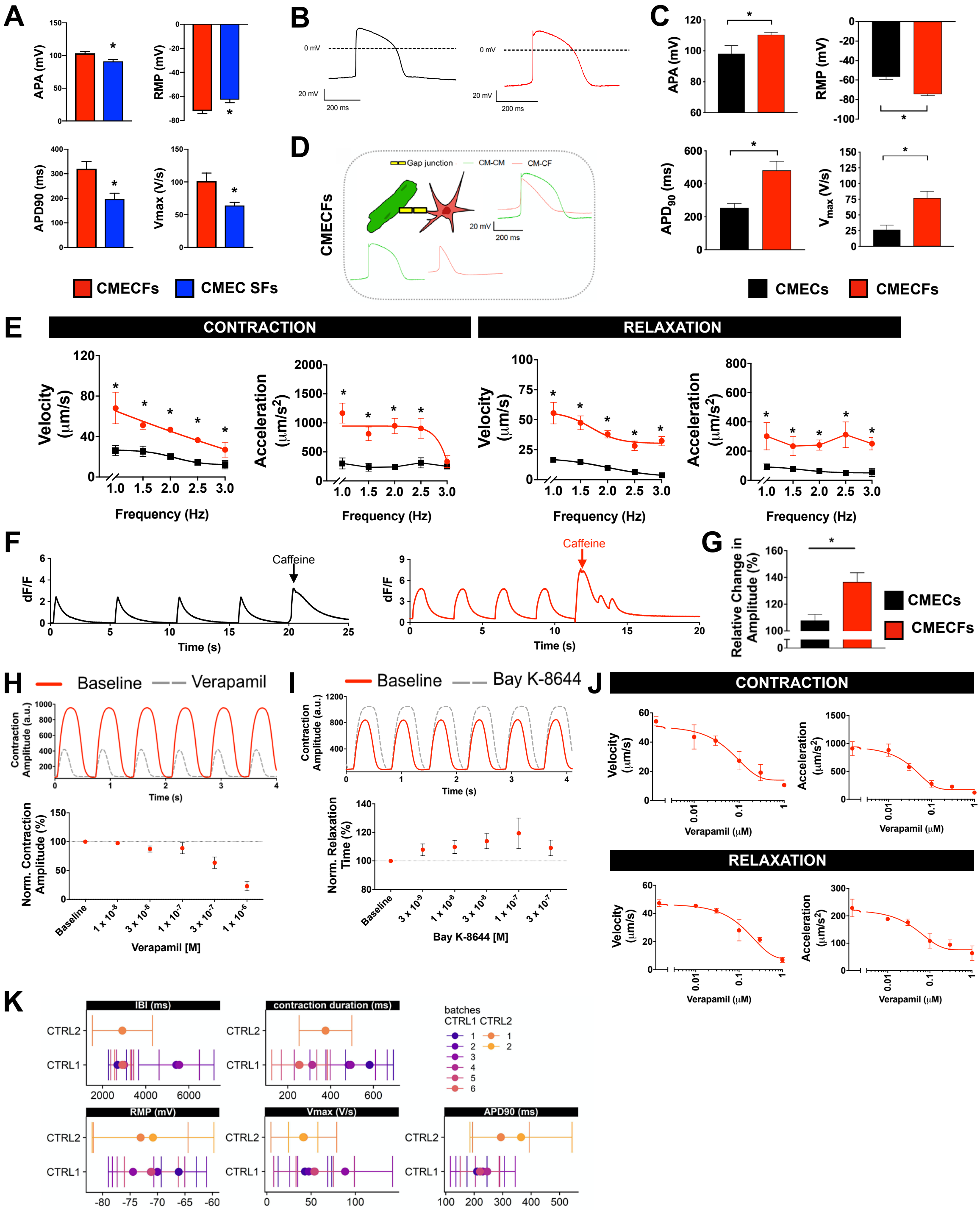

A
B
C
D
E
F
G
H
I
J
K
CMECFs
CMEC SFs
CMECs
CONTRACTION
RELAXATION
Gap junction
CM-CM
CM-CF
Caffeine
Baseline
Verapamil
Bay K-8644
Verapamil (μM)
Verapamil [M]
Bay K-8644 [M]
Frequency (Hz)
Time (s)
Relative Change in Amplitude (%)
IBI (ms)
contraction duration (ms)
RMP (mV)
Vmax (V/s)
APD90 (ms)
batches
CTRL1
CTRL2

Figure S4 (related to Figure 4). Cardiac fibroblasts promote electrical maturation and enhance contractility of hiPSC-CMs in microtissues. (A) Bar graph showing parameters of APs recorded from single hiPSC-CMs (CTRL2) dissociated from CMECFs and CMEC SFs: APA, RMP, APD_{90}; V_{max} (in APs measured with dynamic clamp). N>18, single CMs dissociated from 2 independent MT batches per group, *P>0.05. Student's t-test. Data are shown as mean ± SEM. (B, C) Representative AP traces measured by sharp electrode electrophysiology in CMECs (black) and in CMECFs (red) generated from CTRL1 (B) and AP parameters (C). N>11, MTs per group, *P<0.05. Student's t-test. Data are shown as mean ± SEM. (D) Diagram and representative examples of APs measured in CMECFs by sharp electrode electrophysiology documenting direct coupling between CMs (CM-CM) or CM and CF (CM-CF), together with APs overlay. (E) Rate-dependence analysis for velocity and acceleration of contraction (left panel) together with velocity and acceleration of relaxation (right panel) in CMECs (black) and CMECFs (red). N>22, MTs per group, *P<0.0001. Two-way ANOVA with Sidak's multiple comparison test. Data are shown as mean ± SD. (F) Representative Ca^{2+} transients before and after addition of 50 mM Caffeine in CMECs (black) and CMECFs (red). (G) Change in Ca^{2+} amplitude (percentage, relative to amplitude at baseline condition) measured in CMECs (black) and CMECFs (red). N=8, MTs per group. *P<0.005. Student's t-test. Data are shown as mean ± SEM. (H, I) Representative contraction traces from CMECFs stimulated at 1.5 Hz under baseline condition (red lines) and upon addition of 1 x 10^{-6} M verapamil (H, top) or 1 x 10^{-7} M Bay K-8644 (I, top) (dashed grey lines). Concentration-response curves (contraction amplitude was normalized to baseline) in CMECFs under baseline condition and upon increasing concentrations of Verapamil (H, bottom, N=10) or Bay K-8644 (I, bottom, N=6). Data are shown as mean ± SEM. (J) Concentration-response curves for velocity and acceleration of contraction (top) and relaxation (bottom) of CMECFs under baseline condition and upon increasing concentrations of Verapamil. MTs were stimulated at 1.5 Hz. N>14. Data are shown as mean ± SEM. (K) Graphs showing variability of contraction parameters, i.e. IBI and contraction duration (top panels) and single-cell electrophysiology parameters, i.e. RMP, Vmax, and APD_{90} in CMECFs. Mean values (dots) and SD (error bars) for different MT batches from CTRL1 and CTRL2 hiPSCs are shown.

A

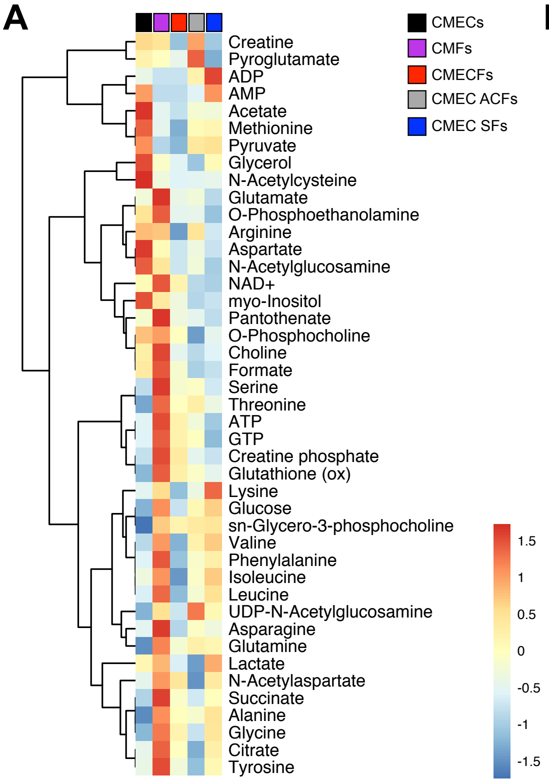

B

	CMECs	CMFs	CMECFs	CMEC ACFs	CMEC SFs
**Lactate**	0.00522	0.01161	0.01235	0.00788	0.01347
**Alanine**	0.00183	0.00182	0.00162	0.00132	0.00249
**Pyro-glutamate**	0.00212	0.00097	0.00090	0.00108	0.00110
**D-glucose**	-0.00038	-0.00898	-0.00803	-0.00764	-0.01375
**Pyruvate**	-0.00094	-0.00080	-0.00056	-0.00121	-0.00090
**Arginine**	-0.00118	-0.00011	-0.00012	0.00000	0.00023
**Glutamine**	0.00142	-0.00004	-0.00020	-0.00030	0.00017
**Proline**	-0.00057	-0.00042	0.00000	-0.00016	-0.00007
**Acetate**	0.00110	-0.00011	0.00027	0.00008	0.00009

Figure S5 (related to Figure 5). Tri-cellular crosstalk between cardiac cell types is essential for metabolic maturation of hiPSC-CMs in microtissues. (A) Heat-map of intracellular metabolites (standardized values) measured in the 5 MT groups. (B) Net release (positive) or uptake (negative) of metabolites to- or from the medium, respectively. Quantities are expressed as µmol, corrected for the total protein mass per sample (µg/ml).

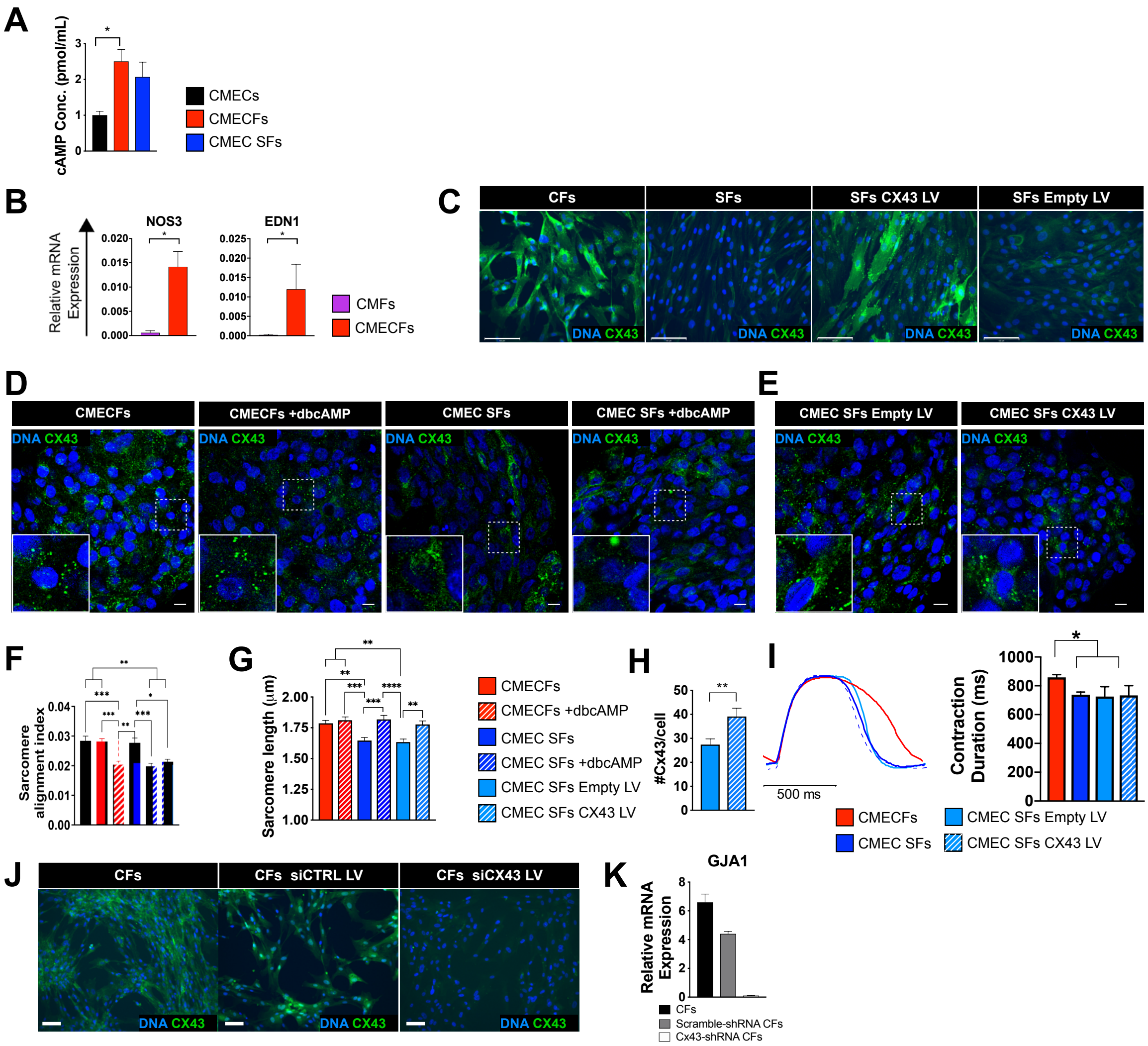

Figure S6 (related to Figure 6). Mechanisms underlying hiPSC-CM maturation in microtissues with cardiac fibroblasts. (A) cAMP concentration (pmol/mL) measured in MTs. N=4, independent MT batches per group, *P<0.05. Friedman test with Dunn's multiple comparisons test. Data are shown as mean ± SEM. (B) qPCR analysis for *NOS3* and *EDN1* genes in CMFs and CMECFs. N>3, independent MT batches; *P<0.05. Student's t-test. Data were normalized to *RPL37A* and are shown as mean ± SEM. (C) Immunofluorescence analysis of CX43 (green) in non-transduced CTRL2 hiPSC-CFs (CFs), CTRL2 primary dermal/skin fibroblasts (SFs), CTRL2 CX43 LV transduced SFs (SFs CX43 LV), CTRL2 Control LV transduced SFs (SFs Empty LV). Nuclei stained with DAPI (blue). Scale bar: 100 µm. (D, E) Representative immunofluorescence images corresponding to those in Figure 6F and 6G) of CX43 (green) alone, to illustrate CX43 distribution in MTs from CTRL1 hiPSCs, either untreated (CMECFs, CMEC SFs) or treated for 7-days with dbcAMP (CMECFs +dbcAMP, CMEC SFs +dbcAMP) (D), and MTs from CTRL1 hiPSCs containing either SFs transduced with control lentivirus (LV) (CMEC SFs Empty LV) or SFs transduced with CX43 lentivirus (LV) (CMEC SFs CX43 LV) (E). Nuclei stained with DAPI (blue). Scale bar: 10 µm. Insets are magnifications of the framed areas and highlight CX43 distribution. (F, G) Quantification of sarcomere organization (sarcomere alignment index) (F) and sarcomere length (G) from immunofluorescence analysis of MTs from CTRL2 hiPSCs (CMECFs and CMEC SFs) and treated for 7-days with dbcAMP (CMECFs +dbcAMP and CMEC SFs +dbcAMP), MTs from CTRL2 hiPSC-CMs and ECs with CTRL2 primary dermal/skin fibroblasts transduced with control lentivirus (LV) (CMEC SFs Empty LV) or CTRL2 primary dermal/skin fibroblasts transduced with CX43 lentivirus (LV) (CMEC SFs CX43 LV). N=30, different areas from 3 MTs per group, *P<0.01, **P<0.005, ***<P=0.0005, ****P<0.0001. One-way ANOVA with Tukey's multiple comparisons test. All data shown as mean ± SEM. (H) Quantification of CX43 per cell in MTs. **P<0.01. Data shown as mean ± SEM. N=15. Student's t-test. (I) Representative contraction traces (left, normalized amplitude is shown) and bar graph of mean contraction duration (right panel) in MTs paced at 1Hz. N=17, MTs per group. *P<0.0001. One-way ANOVA with Fisher's LSD test. Data shown as mean ± SEM. (J) Immunofluorescence analysis of CX43 (green) in non-transduced CTRL1 hiPSC-CFs (CFs), scrambled shRNA CTRL1 hiPSC-CFs and CX43-shRNA CTRL1 hiPSC-CFs. Nuclei stained with DAPI (blue). Scale bar: 100 µm. (K) qPCR analysis for *GJA1* (encoding for CX43) in non-transduced CTRL1 hiPSC-CFs (CFs), scrambled shRNA CTRL1 hiPSC-CFs and CX43-shRNA CTRL1 hiPSC-CFs. Values are normalized to *HARP* and *RPL37A*. N=2, independent transductions. Data shown as mean ± SD.

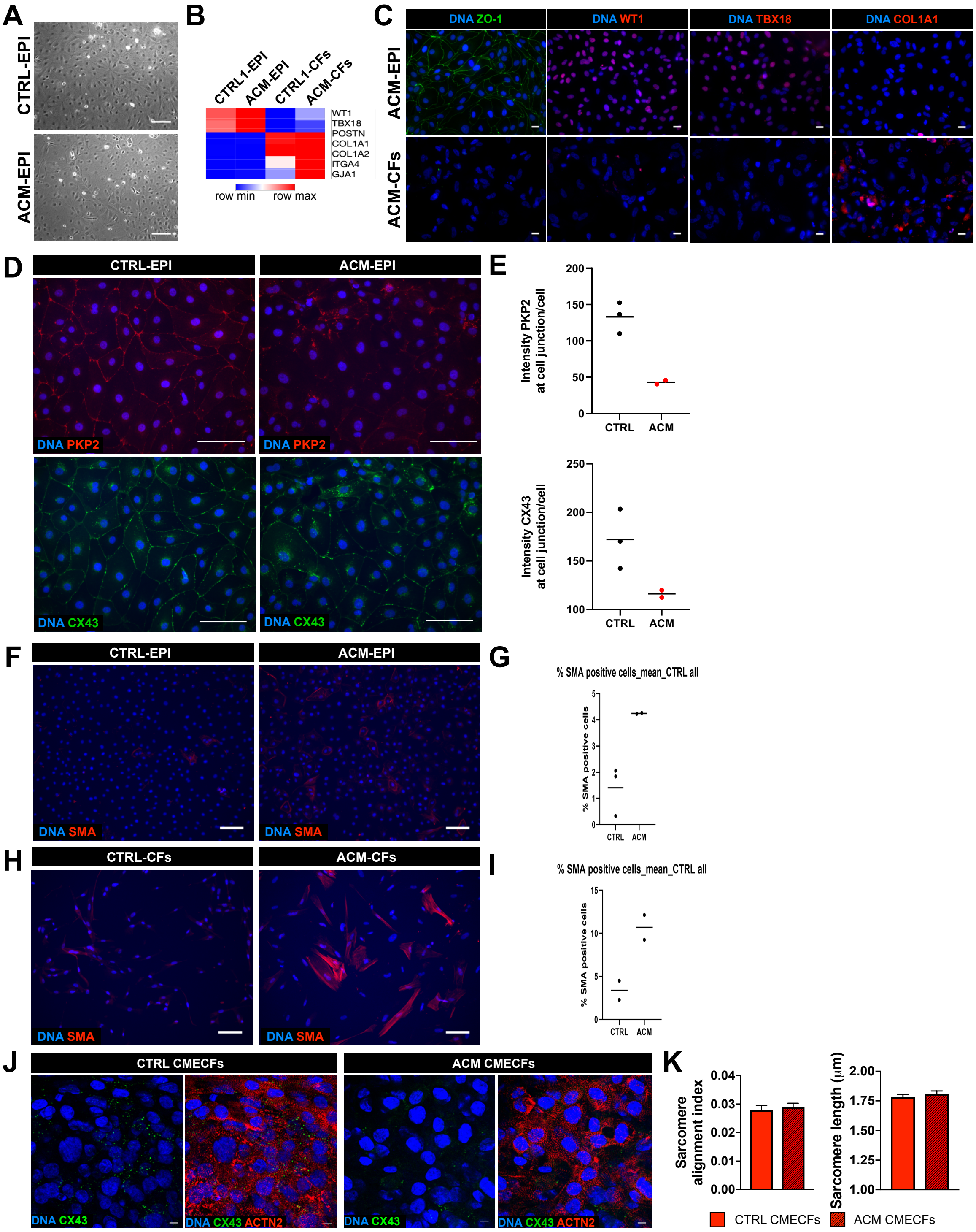
A
CTRL-EPI
ACM-EPI
B
CTRL1-EPI
ACM-EPI
CTRL1-CFs
ACM-CFs
WT1
TBX18
POSTN
COL1A1
COL1A2
ITGA4
GJA1
row min
row max
C
DNA ZO-1
DNA WT1
DNA TBX18
DNA COL1A1
ACM-EPI
ACM-CFs
D
CTRL-EPI
ACM-EPI
DNA PKP2
DNA PKP2
DNA CX43
DNA CX43
E
Intensity PKP2 at cell junction/cell
200
150
100
50
0
CTRL
ACM
Intensity CX43 at cell junction/cell
250
200
150
100
CTRL
ACM
F
CTRL-EPI
ACM-EPI
DNA SMA
DNA SMA
G
% SMA positive cells_mean_CTRL all
% SMA positive cells
5
4
3
2
1
0
CTRL
ACM
H
CTRL-CFs
ACM-CFs
DNA SMA
DNA SMA
I
% SMA positive cells_mean_CTRL all
% SMA positive cells
15
10
5
0
CTRL
ACM
J
CTRL CMECFs
ACM CMECFs
DNA CX43
DNA CX43 ACTN2
DNA CX43
DNA CX43 ACTN2
K
Sarcomere alignment index
0.04
0.03
0.02
0.01
0.00
Sarcomere length (μm)
2.00
1.75
1.50
1.25
1.00
CTRL CMECFs
ACM CMECFs

Figure S7 (related to Figure 7). Microtissues as model of Arrhythmogenic Cardiomyopathy (ACM). (A) Representative bright field images of CTRL- and ACM-EPI. Scale bar: 100 µm. (B) Heat-map showing qPCR expression for fibroblast- (*ITGA4, COL1A2, COL1A1, POSTN*) and EPI- (*WT1, TBX18*) genes in hiPSC-EPI and -CFs from CTRL1 and ACM lines. Values were normalized to *RPL37A*. N>3 independent differentiations. (C) Representative immunofluorescence images for ZO-1 (green) and WT1, TBX18, COL1A1 (red) in ACM hiPSC-EPI and ACM hiPSC-CFs. Nuclei stained with DAPI (blue). Scale bar: 20 µm. (D) Immunofluorescence analysis of PKP2 (red, upper panel) and CX43 (green, lower panel) in hiPSC-EPI from CTRL1 hiPSCs (CTRL-EPI) and ACM hiPSCs (ACM-EPI). Nuclei stained with DAPI (blue). Scale bar: 100 µm. (E) Quantification of PKP2 (upper panel) and CX43 (lower panel) intensity at the cell-cell junctions in CTRL-EPI and ACM-EPI. (F) Immunofluorescence analysis of SMA (red) in CTRL-EPI and ACM-EPI. Nuclei stained with DAPI (blue). Scale bar: 100 µm. (G) Quantification of SMA-positive cells in CTRL- and ACM-EPI. CTRL-EPI, N=3 (two independent differentiations from CTRL1 and one from CTRL2); ACM-EPI, N=2 (two independent differentiations). (H) Immunofluorescence analysis of SMA (red) in hiPSC-CFs from CTRL1 hiPSCs (CTRL-CFs) and ACM hiPSCs (ACM-CFs). Nuclei stained with DAPI (blue). Scale bar: 100 µm. (I) Quantification of SMA positive cells in CTRL- and ACM-CFs. CTRL-CFs, N=2 (two independent differentiations from CTRL1 and CTRL2 hiPSCs); ACM-CFs, N=2 (two independent differentiations from ACM hiPSCs). (J) Immunofluorescence analysis of CX43 (green) and cardiac sarcomeric protein ACTN2 (red) in CTRL CMECFs or ACM CMECFs. Nuclei stained with DAPI (blue). Scale bar: 10 µm. (K) Quantification of sarcomere organization (sarcomere alignment index, left) and sarcomere length (right) in CTRL CMECFs and ACM CMECFs. N=30, different areas from 3 MTs per group.

Table S6. List of oligonucleotides used for qPCR.

Oligonucleotides	SOURCE	IDENTIFIER
**Primer sequences for qPCR**		
RPL37A Forward: GTGGTTCCTGCATGAAGACAGTG	Zhang et al. 2014	N/A
RPL37A Reverse: TTCTGATGGCGGACTTTACCG	Zhang et al. 2014	N/A
HARP Forward: CACCATTGAAATCCTGAGTGATGT	Devalla et al. 2015	N/A
HARP Reverse: TGACCAGCCCAAAGGAGAAG	Devalla et al. 2015	N/A
WT1 Forward: TATTCTGTATTGGGCTCCGC	Guadix et al. 2017	N/A
WT1 Reverse: CAGCTTGAATGCATGACCTG	Guadix et al. 2017	N/A
TBX18 Forward: TTGCTAAAGGCTTCCGAGAC	Guadix et al. 2017	N/A
TBX18 Reverse: AGGTGGAGGAACTTGCATTG	Guadix et al. 2017	N/A
GJA1 Forward: TGGTAAGGTGAAAATGCGAGG	PrimerBank	N/A
GJA1 Reverse: GCACTCAAGCTGAATCCATAGAT	PrimerBank	N/A
ITGA4 Forward: AGCCCTAATGGAGAACCTTGT	PrimerBank	N/A
ITGA4 Reverse: CCAGTGGGGAGCTTATTTTCAT	PrimerBank	N/A
COL1A1 Forward: GAGGGCCAAGACGAAGACATC	PrimerBank	N/A
COL1A1 Reverse: CAGATCACGTCATCGCACAAC	PrimerBank	N/A
COL1A2 Forward: GAGCGGTAACAAGGGTGAGC	PrimerBank	N/A
COL1A2 Reverse: CTTCCCCATTAGGGCCTCTC	PrimerBank	N/A
POSTN Forward: CTCATAGTCGTATCAGGGGTCG	PrimerBank	N/A
POSTN Reverse: ACACAGTCGTTTTCTGTCCAC	PrimerBank	N/A
NOS3 Forward: TGATGGCGAAGCGAGTGAAG	PrimerBank	N/A
NOS3 Reverse: ACTCATCCATACACAGGACCC	PrimerBank	N/A
EDN1 Forward: AGAGTGTGTCTACTTCTGCCA	PrimerBank	N/A
EDN1 Reverse: CTTCCAAGTCCATACGGAACAA	PrimerBank	N/A

PrimerBank: https://pga.mgh.harvard.edu/primerbank/
